# Investigation of the enantioselectivity of acetylcholinesterase and butyrylcholinesterase upon inhibition by tacrine-iminosugar heterodimers

**DOI:** 10.1080/14756366.2022.2150762

**Published:** 2022-12-01

**Authors:** I. Caroline Vaaland, Óscar López, Adrián Puerta, Miguel X. Fernandes, José M. Padrón, José G. Fernández-Bolaños, Magne O. Sydnes, Emil Lindbäck

**Affiliations:** a Department of Chemistry, Bioscience and Environmental Engineering, Faculty of Science and Technology, University of Stavanger, Stavanger, Norway; b Departamento de Química Orgánica, Facultad de Química, Universidad de Sevilla, Seville, Spain; c BioLab, Instituto Universitario de Bio-Orgánica “Antonio González” (IUBO-AG), Universidad de La Laguna, c/Astrofísico Francisco Sánchez, La Laguna, Spain

**Keywords:** Cholinesterases, inhibitors, enantiomers, modelling, Alzheimer’s disease

## Abstract

The copper-catalysed azide-alkyne cycloaddition was applied to prepare three enantiomeric pairs of heterodimers containing a tacrine residue and a 1,4-dideoxy-1,4-imino-D-arabinitol (DAB) or 1,4-dideoxy-1,4-imino-L-arabinitol (LAB) moiety held together *via* linkers of variable lengths containing a 1,2,3-triazole ring and 3, 4, or 7 CH_2_ groups. The heterodimers were tested as inhibitors of butyrylcholinesterase (BuChE) and acetylcholinesterase (AChE). The enantiomeric heterodimers with the longest linkers exhibited the highest inhibition potencies for AChE (IC_50_ = 9.7 nM and 11 nM) and BuChE (IC_50_ = 8.1 nM and 9.1 nM). AChE exhibited the highest enantioselectivity (*ca*. 4-fold). The enantiomeric pairs of the heterodimers were found to be inactive (GI_50_ > 100 µM), or to have weak antiproliferative properties (GI_50_ = 84–97 µM) against a panel of human cancer cells.

## Introduction

**Figure 1.**
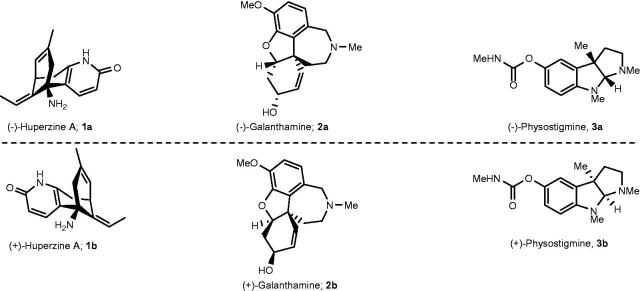
Examples of enantiomeric pairs of ChE inhibitors of which the mirror images display different potencies.

Enzyme inhibition represents an attractive target for drug development^1^. Because enzymes are built up by chiral building blocks, amino acids, it is not surprising if only one member of an enantiomeric pair causes inhibition upon binding. Another alternative is that both enantiomers display various degrees of inhibition, due to different interaction modes^2–4^. One such example is the natural enantiomer **1a** (Figure 1) of huperzine A, which is a 38- to 49-fold more potent AChE inhibitor than its unnatural enantiomer **1b**^5^^,^^6^. In fact, inhibition of cholinesterases (ChEs) is an attractive target for treatment of Alzheimer’s disease (AD) and there are currently three ChE inhibitors on the list of FDA approved AD drugs^7^. (–)-Huperzine (**1a**) is not on the list of FDA approved drugs, but it was approved in China as a symptomatic AD drug^8^^,^^9^.The much stronger AChE inhibition exhibited by **1a** compared to **1b**, was partially rationalised by comparison of the X-ray structures of *Torpedo californica* acetylcholinesterase (*Tc*AChE) complexed with enantiomers **1a** and **1b**, which demonstrated the presence and absence of an interaction between the ethylidene methyl of **1a** and **1b**, respectively, with His440^10^, which is a member of the catalytic triad almost on the bottom of a *ca*. 20 Å deep active gorge of *Tc*AChE^11^.

(-)-Galantamine (**2a**) (Figure 1) is an FDA approved ChE inhibitor drug for the treatment of mild-to-moderate AD^7^. This alkaloid is a reversible AChE inhibitor and exhibits 53-times selectivity for AChE over BuChE^12^. X-ray studies of the *Tc*AChE/(-)-galantamine complex revealed that the inhibitor binds in its acidic form at the base of the active gorge in the region between the acetyl hole and the catalytic anionic site (CAS)^13^. The protonated amine group is quite remote from Trp84 in CAS and thereby is not involved in any cation–π interactions with the Trp84 residue, which is in stark contrast to acetylcholine (ACh), whose quaternary ammonium group establishes a cation–π interaction with Trp84. Instead, the high affinity of (-)-galantamine for AChE was attributed to multiple moderate and weak interactions with the enzyme^13^. The inhibition of AChE by galantamine appears to be enantioselective, at 20 μM inhibitor concentration, as the natural enantiomer **2a** (*ca*. 94% of inhibition) is a much stronger inhibitor than its unnatural antipode **2b** (*ca*. 4% of inhibition)^14^, which indicates that several interactions with the enzyme are eliminated or attenuated when the configuration in all stereogenic centres of **2a** is reversed.

**Figure 2.**
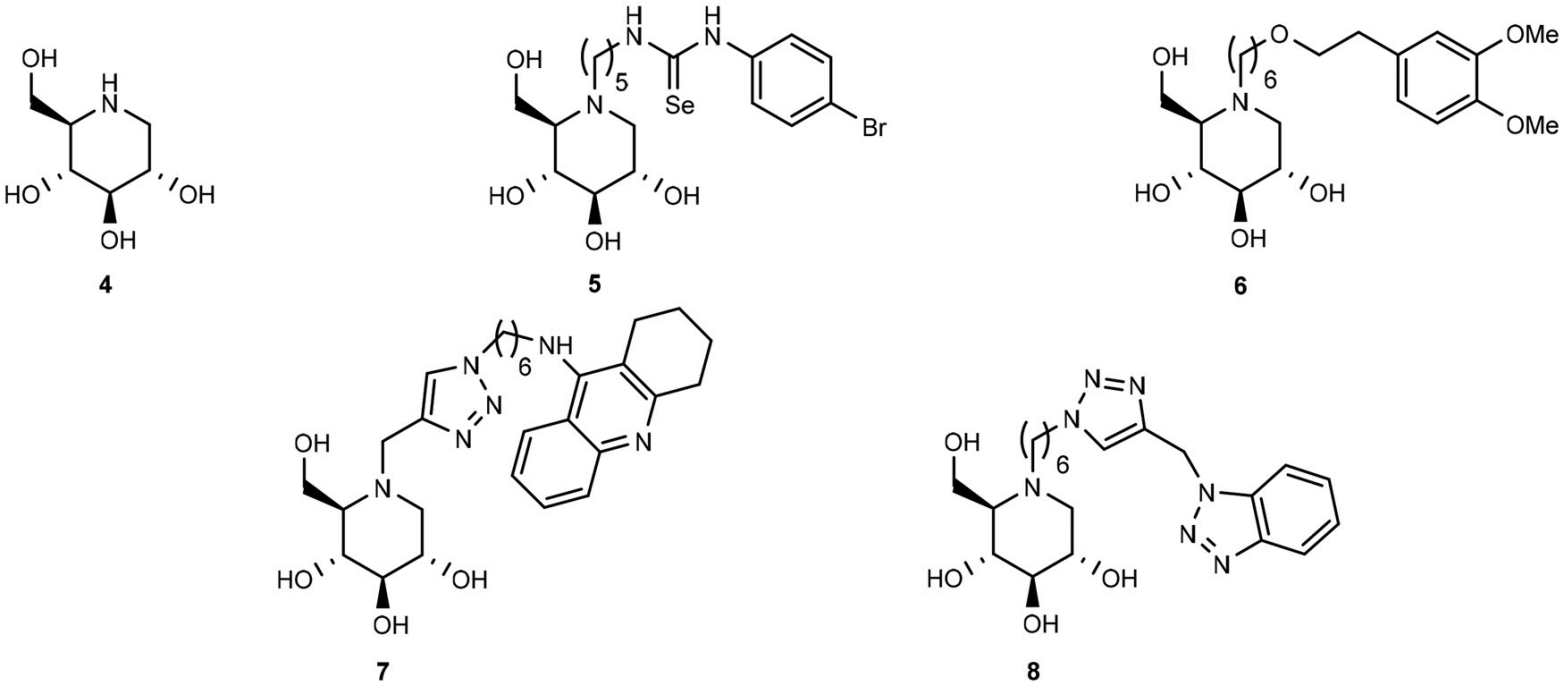
Selected examples of iminosugars that have been investigated as ChE inhibitors.

Significant enantioselectivity has also been observed for the inhibition of AChE by physostigmine; natural (-)-physostigmine (**3a**) (Figure 2) is a *ca*. 25- to 1000-fold stronger inhibitor, depending on the enzyme source, than (+)-physostigmine (**3b**)^15^^,^^16^.

Iminosugars are glycomimetics in which the ring oxygen atom has been replaced by a nitrogen atom^17^. Iminosugars are attractive as pharmaceutical candidates because they inhibit glycosidases without being metabolised by such enzymes^18^. Such properties have made iminosugars attractive as synthetic targets^19^ and lead compounds for the treatment of various diseases such as viral infections, diabetes, type 2 diabetes, and lysosomal disorders^20^. In addition, it has been found that iminosugars are able to inhibit the growth of cancer cells^21^^,^^22^, without affecting the viability and mortality of normal cells^21^. To date, three iminosugars, namely, miglitol^23^, miglustat^24^, and migalastat^25^ have been approved by FDA for the treatment of type 2 diabetes, Gaucher’s disease, and Fabry’s disease, respectively. Miglustat has also been found to reduce the production of amyloid β-peptide (Aβ)^26^, which is a component of senile plaque in AD patients.

The ester group of ACh is held in place for hydrolysis by the catalytic triad in the active gorge of AChE by the aid of cation − π interactions with a Trp residue in CAS^27^. Because many iminosugars are protonated at physiological pH^28^, they were proposed to be capable of inhibiting ChEs^29^. Thereby, a series of iminosugars of various stereochemistry and substitution patterns have been tested as ChE inhibitors, displaying particularly good BuChE inhibition^29^. Following this line, some of us have reported the synthesis and ChE inhibitory testing of bivalent inhibitors in which a 1-deoxynojirimycin (1-DNJ) (**4**) binding unit is connected to a second binding unit, namely, aryl-substituted selenourea (exemplified by **5**)^30^, catechol (exemplified by **6**)^31^, tacrine (exemplified by **7**)^32^, or benzotriazole (exemplified by **8**)^33^ binding unit (Figure 2). Kinetic assays and modelling studies for the binding of **6**, **7**, and **8** to AChE indicated that they behave as dual binding site AChE inhibitors, which implies that they bind simultaneously to the peripheral anionic site (PAS) and CAS. A more surprising observation (given that the quaternary ammonium group of ACh participates in a cation–π interaction with a Trp residue in CAS) from the modelling studies was that when heterodimers **6** and **8** bind to AChE in their protonated states (on the 1-DNJ nitrogen atom), the positive charged nitrogen atom is not necessarily involved in cation − π interactions with the aromatic residues of the enzyme^31^^,^^33^.

**Scheme 1.**
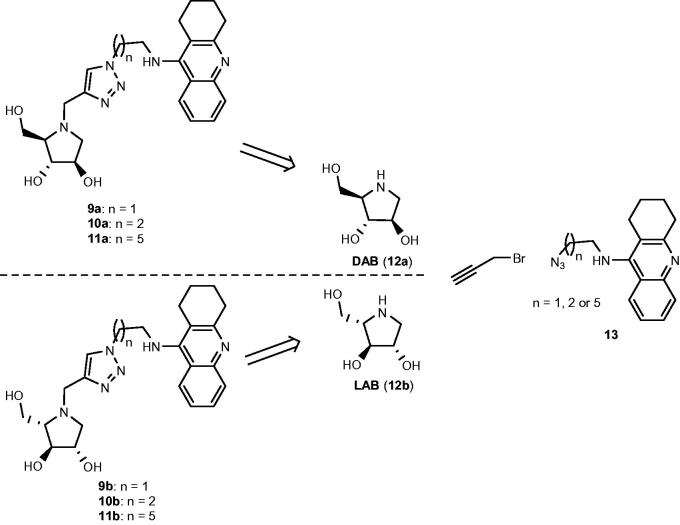
Retrosynthetic pathways to the optically pure pairs of enantiomers **9a** and **9b**, **10a** and **10b**, and **11a** and **11b**.

Thus far, five papers have been published, which demonstrate the potential of iminosugars as ChE inhibitors^29–33^. One entry of ChE inhibition by iminosugars that remains to be studied is whether iminosugars can achieve enantioselective ChE inhibition. Thus, in this paper, we present the synthesis of three pairs of optically pure iminosugar-tacrine heterodimer enantiomers, namely, **9a** and **9b**, **10a** and **10b**, and **11a** and **11b** (Scheme 1) and the evaluation of their performance as ChE inhibitors. The study also includes docking studies of the heterodimers to predict interaction with AChE and BuChE. Naturally occurring 1,4-dideoxy-1,4-imino-D-arabinitol (DAB) (**12a**) constitutes the iminosugar moiety in **9a**, **10a**, and **11a**, whereas non-natural 1,4-dideoxy-1,4-imino-L-arabinitol (LAB) (**12b**) is the iminosugar moiety in **9b**, **10b**, and **11b.** Because both iminosugars^21^^,^^22^, and heterodimers containing a tacrine moiety^34^ have been found to inhibit the growth of cancer cells, we also report the antiproliferative screening of **11a** and **11b** against a panel of six cancer cell lines.

## Materials and methods

### General procedures

Dichloromethane (DCM), methanol (MeOH), acetone, dimethyl sulfoxide (DMSO) and dimethylformamide (DMF) were dried over 4 Å molecular sieves (oven dried). Petroleum ether (PE) from the 40–65 °C fraction was used for silica flash columns. All reactions were carried out under Ar atmosphere if not otherwise specified. Reactions performed at room temperature (rt) refer to the temperature range of 20 to 22 °C. TLC analyses were performed on Merck silica gel 60 F_254_ plates using UV light (λ = 254 nm) for detection. Silica gel NORMASIL 60^®^ 40–63 µm was used for silica flash columns. A Bruker Avance NMR spectrometer was used to record ^1^H-NMR spectra (400.13 MHz) and ^13^C-NMR spectra (100.61 MHz) in CDCl_3_, CD_3_OD, or D_2_O. Chemical shifts (δ) are reported relative to residual DMSO (δ 2.50 ppm, ^1^H; δ 39.52 ppm, ^13^C), residual CHCl_3_ in CDCl_3_ (δ 7.26 ppm, ^1^H; δ 77.16 ppm, ^13^C), residual CD_3_OD (δ 3.31 ppm, ^1^H; δ 49.0 ppm, ^13^C), residual D_2_O (δ 4.79 ppm, ^1^H) and TMS as an internal standard in CDCl_3_. High-resolution mass spectra (HRMS) were recorded on a Qexactive spectrometer in positive electrospray ionisation (ESI) mode.

#### Synthetic protocols

##### General procedure for the preparation of compounds 20a–22a and 20b–22b

A mixture of **19b** (1 equiv., 0.04 M for synthesis of **20b**, **21b**, and **22b**) or **19a** (1 equiv., 0.07 M for synthesis of **20a**, 0.04 M for synthesis of **21a**, and 0.05 M for synthesis of **22a**), azide **13** (0.98 equiv.), and copper(II) sulphate pentahydrate (0.30 equiv.) in anhydrous DMF in an aluminium foil covered round bottom flask was degassed and introduced an argon atmosphere before the addition of sodium ascorbate (0.60 equiv.). After addition, the mixture was kept stirring for 48 h at rt. The solvent was then removed under reduced pressure and the residue obtained was purified by silica gel column chromatography (See Supplementary Material for details).

##### General procedure for the preparation of compounds 9a–11a and 9b–11b

To a mixture of **20a–22a** (0.02 M, 1 equiv.) or **20b***–***22b** (0.02 M, 1 equiv.) in anhydrous CH_2_Cl_2_ under an argon atmosphere at −78 °C was slowly added BCl_3_ (1 M in heptane, 15 equiv.). After addition, the mixture was kept stirring at −78 °C for 2 h and then at 0 °C overnight. The volatiles were then removed under reduced pressure and the concentrate underwent purification by gradient silica gel chromatography (MeCN/H_2_O/NH_4_OH 190:10:1 → 180:20:1) (column 1). The corresponding HCl salt was dissolved in MeOH (2 ml) and NH_4_OH (0.5 ml) and kept stirring for 48 h. The solvent was removed under reduced pressure and the resulting residue was purified by gradient silica gel chromatography (column 2) (the solvent gradient for column 2 for each single experiment is specified in the Supplementary Material).

#### Inhibition assays

Measuring of the inhibition activity of compounds **9a**–**11a** and **9b**–**11b** against cholinesterases (AChE from *Electrophorus electricus* and BuChE from equine serum) was accomplished following minor modifications of the Ellman assay^35^, as reported previously^36^. A Thermo Scintific^TM^ Varioskan^TM^ LUX microplate reader and Greiner F-bottom 96-well plates were used. Cornish-Bowden plots (1/V *vs.* [I] and [S]/V *vs.* [I]) were used for the visualisation of the mode of inhibition. Calculation of the kinetic parameters (*K*_M_, V_max_) was accomplished using a nonlinear regression analysis (least squares fit) implemented in GraphPad Prism 8.01 software; such parameters were in turn used for calculating the inhibition constants of the mixed inhibitors using the following equations:

$$K_{m,\mathrm{app}}=K_{M}\frac{1+\frac{\left[I\right]}{K_{i}}}{1+\frac{\left[I\right]}{\alpha K_{i}}}$$

$$V_{\max,\mathrm{app}}=\frac{V_{\max}}{1+\frac{\left[I\right]}{\alpha K_{i}}}$$

#### General method for docking simulations

**Scheme 2.**
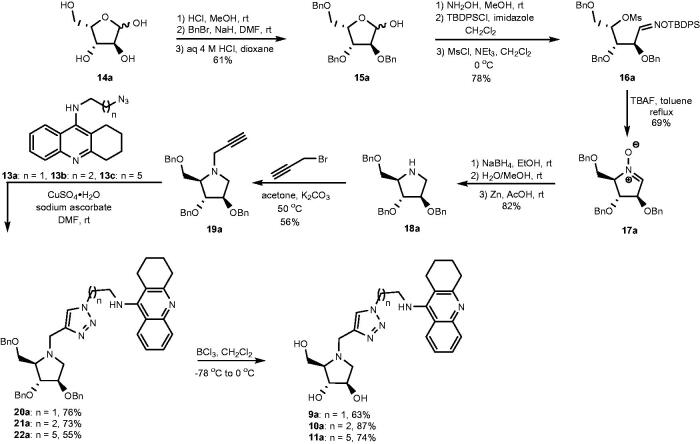
Synthesis of heterodimers **9a**–**11a**.

Interactions of enzymes with compounds were analysed by computational docking using Molecular Operating Environment (MOE) software (Chemical Computing Group ULC, Montreal, Canada). Crystallographic structures of human AChE and human BuChE was obtained from Protein Data Bank (PDB code 4EY6^37^ and 4AQD^38^, respectively). Protein structures were prepared using Amber10 force field with EHT parameters, R-field solvation model, dielectric constant of 1 for the protein interior and 80 for exterior. Ligand structures were drawn in MOE software, and their energies were minimised using Amber10 force field with EHT parameters for small molecules, using as stop criterion an RMS gradient lower than 0.01 kcal/mol/Å. For the docking calculations: in the placement stage we used the Triangle Matcher algorithm with the London dG scoring scheme. In the refinement stage we kept the receptor rigid and used the GBVI/WSA dG scoring scheme. 2D diagrams were obtained from MOE software and 3D illustrations were obtained using Pymol software.

#### Antiproliferative activity assays

For the antiproliferative tests, we applied our implementation of the National Cancer Institute (NCI) screening protocol^39^. As a model of human solid tumour cells, we selected the cell lines A549 (non-small cell lung), HBL-100 (breast), HeLa (cervix), SW1573 (non-small cell lung), T-47D (breast), and WiDr (colon). Cell seeding densities, based on the cell line doubling time, were 2500 (A549, HBL-100, HeLa and SW1573) or 5000 (T-47D and WiDr) cells/well. Compounds were initially dissolved in DMSO at 400 times the desired final maximum test concentration. Control cells were exposed to an equivalent concentration of DMSO (0.25% v/v, negative control). Each compound was tested in triplicate at different dilutions ranging from 1 to 100 μM. Drug treatment began on day 1 after sowing. The drug incubation times were 48 h, after which the cells were precipitated with ice-cold TCA (50% w/v) and fixed for 60 min at 4 °C. Then the SRB test was performed. The optical density (OD) of each well was measured at 530 nm using a microplate absorbance reader (PowerWave XS, BioTek Instruments Inc.). Values were corrected for background OD of wells containing medium only^39^. The results were expressed as GI_50_, i.e. the dose that causes 50% growth inhibition after 48 h of exposure.

## Synthesis

The synthesis of heterodimers **9a**, **10a**, and **11a** commenced from L-xylose (**14a**), which was converted into 2,3,5-tri-*O*-benzyl-L-xylofuranose (**15a**) by following a reported three step procedure (Scheme 2)^40^. The obtained furanose underwent three subsequent chemical modifications including: (1) aldoxime formation, (2) selective *O*-silylation of the oxime oxygen atom, and (3) mesylation to provide compound **16a**^41^ in 78% yield after purification by silica gel chromatography. When **16a** was treated with F^-^ ions it cyclized into nitrone **17a**^42^^,^^43^ upon loss of the *O*-silyl group. Tetra-*O*-benzylated DAB **18a** was obtained in 82% yield when nitrone **17a** was reduced first by sodium borohydride and followed by zinc in acetic acid^44^. In the following step, **18a** underwent *N*-propargylation to form alkyne **19a** when it was treated with propargyl bromide. This alkyne underwent copper-catalysed azide – alkyne cycloaddition^45^ with azides **13a**^46^, **13b**^32^, and **13c**^32^ to form heterodimers **20a**, **21a**, and **22a**, respectively. In the final step, heterodimers **20a**, **21a**, and **22a** underwent BCl_3_ promoted de-*O*-benzylation to generate target compounds **9a**, **10a**, and **11a**, respectively.

**Scheme 3.**
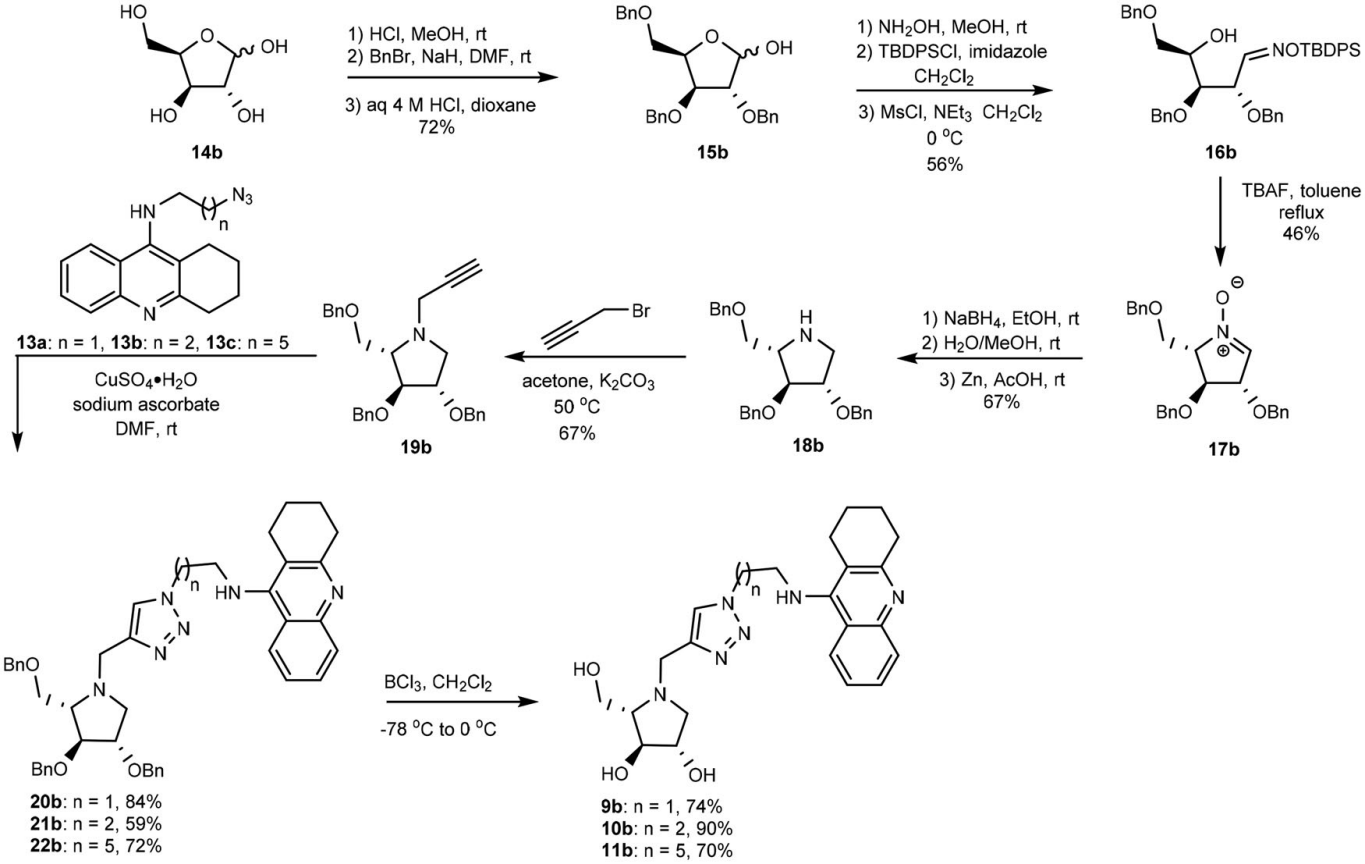
Synthesis of heterodimers **9b**, **10b**, and **11b**.

The synthesis of **9b**, **10b**, and **11b** were performed in the same way as for **9a**, **10a**, and **11a** by replacing L-xylose (**14a**) with D-xylose (**14b**) in the first step (Scheme 3).

## ChE inhibitory testing

The minimum inhibitory concentrations of the enantiomeric pairs **9a** and **9b**, **10a** and **10b**, and **11a** and **11b** required to reach 50% inhibition (IC_50_) of *ee*AChE and eqBuChE are presented in Table 1. A minor modification of the Ellman assay was used in order to measure the IC_50_ values^35^. The test series included (-)-galantamine (**2a**) and tacrine as positive references.

**Table 1.** IC_50_ values for the inhibition of *ee*AcHE and eqBuChE by **9a**, **10a**, and **11a** and with their mirror images **9b**, **10b**, and **11b**. 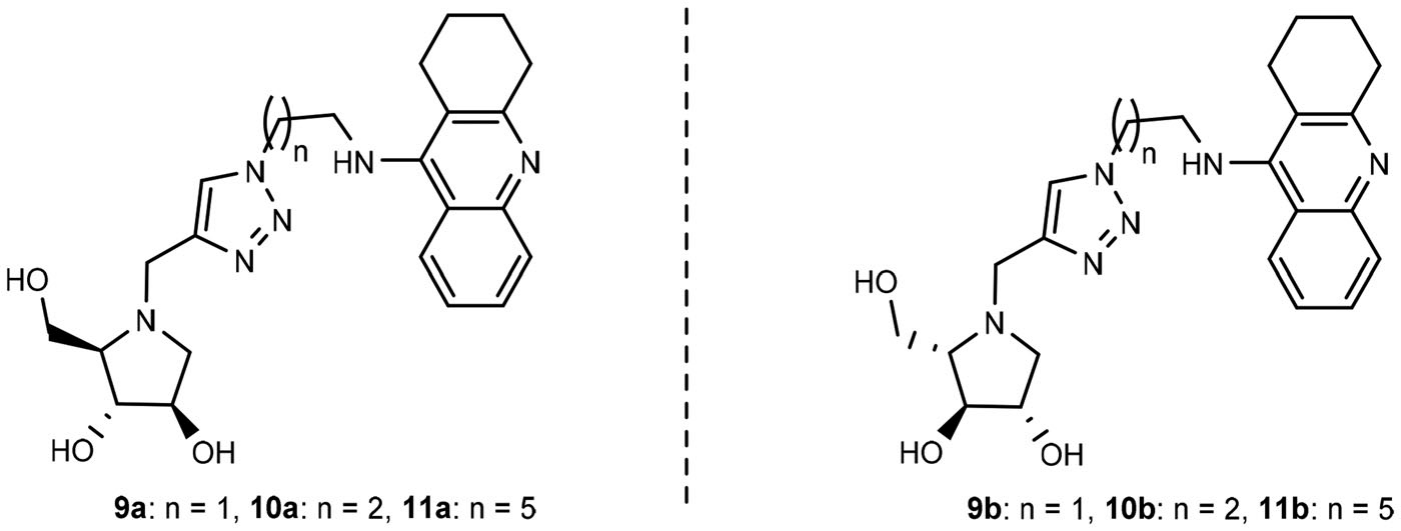

	IC_50_ (nM)^a^			
Compound	*ee*AChE^b^	eqBuChE^c^	Enantioselectivity (*ee*AChE)^d^	Enantioselectivity (eqBuChE)^e^
(-)-Galantamine (**2a**)	1300 ± 100	5500 ± 400	–	–
Tacrine	290 ± 2	2.8 ± 0.2	–	–
**9a**	420 ± 10	96 ± 5	–	–
**9b**	1480 ± 240	179 ± 25	0.28	0.54
**10a**	530 ± 30	184 ± 16	–	–
**10b**	150 ± 38	232 ± 28	3.5	0.79
**11a**	9.7 ± 1	9.1 ± 0.3	–	–
**11b**	10.7 ± 0.3	8.1 ± 0.1	0.91	1.1
	*K*_i_ = 19.0 ± 1.8 nM	*K*_i_ = 10.0 ± 2.7 nM		
	α*K*_i_ = 21.9 ± 7.2 nM	α*K*_i_ = 14.3 ± 3.3 nM		
	(mixed)	(mixed)		

^a^Mean ± SD.

^b^[S] = 121 µM.

^c^[S] = 112 µM.

^d^IC_50_(**Xa**:AChE)/IC_50_(**Xb**:AChE).

^e^IC_50_(**Xa**:BuChE)/IC_50_(**Xb**,BuChE). (*K*_i_: competitive inhibition constant and α*K*_i_: uncompetitive inhibition constant).

Both series of stereoisomers **9a**–**11a **(incorporating a DAB moiety) and **9b**–**11b** (incorporating a LAB moiety) displayed IC_50_ values from the submicromolar concentration range down to the nanomolar concentration range for the inhibition of *ee*AChE and eqBuChE. The only exception was **9b**, which exhibits a IC_50_ value of 1480 nM for the inhibition of *ee*AChE. Thereby, **9b** was the only compound in the testing series that is a less potent AChE inhibitor than (-)-galantamine, which is in in current use against AD^47^.

The length of the linker between the tacrine ring and iminosugar moiety had a significant impact on the inhibition potency of both *ee*AChE and eqBuChE in which a longer linker provided higher inhibition potencies. This was demonstrated by the result that **11a** (*n* = 5, IC_50_ = 9.7 nM against *ee*AChE) is a *ca.* 43-fold more potent *ee*AChE inhibitor than **9a** (*n* = 1, IC_50_ = 420 nM against *ee*AChE) and a 55-fold more potent *ee*AChE inhibitor than **10a** (*n* = 2, IC_50_ = 530 nM against *ee*AChE). A similar trend was observed when the enantiomer of **11a**, namely, **11b** (*n* = 5, 10.7 nM against *ee*AChE) was compared with **9b** (*n* = 1, 1480 nM against *ee*AChE) and **10b** (*n* = 2, 150 nM against *ee*AChE) for the inhibition of the same enzyme as **11b** is a *ca.* 138- and 14-fold stronger inhibitor than **9b** and **10b**, respectively. Six CH_2_-groups between the 1,2,3-triazole and tacrine moiety was also most favourable for the inhibition of eqBuChE as **11a** (*n* = 5, IC_50_ = 9.1 nM against eqBuChE) is a roughly 11-fold stronger inhibitor than **9a** (*n* = 1, IC_50_ = 96 nM against eqBuChE) and a 20-fold stronger inhibitor than **10a** (*n* = 2, IC_50_ = 184 nM against eqBuChE). Likewise, **11b** (*n* = 5, IC_50_ = 8.1 nM against eqBuChE) is a 22- and 29-fold stronger eqBuChE inhibitor than **9b** (*n* = 1, IC_50_ = 179 nM against eqBuChE) and **10b** (*n* = 2, IC_50_ = 232 μM against eqBuChE), respectively.

No obvious enantioselectivity of *ee*AChE and eqBuChE was observed for the three pairs of enantiomeric inhibitors included in this study. In addition, no preferential inhibitory activity trend was found for the enantiomers incorporating a DAB or LAB moiety. For instance, **9a** is a *ca.* 4-fold more potent *ee*AChE inhibitor than its enantiomer **9b**, whereas **10b** is a *ca.* 4-fold more potent *ee*AChE inhibitor than its enantiomer **10a**. For the enantiomeric pair **11a** and **11b**, we observed essentially equal *ee*AChE inhibitory activities. These observations indicate that the impact on the *ee*AChE inhibitory potency of our heterodimers by switching between a DAB and LAB moiety is small compared to the contribution from the tacrine ring.

**Figure 3.**
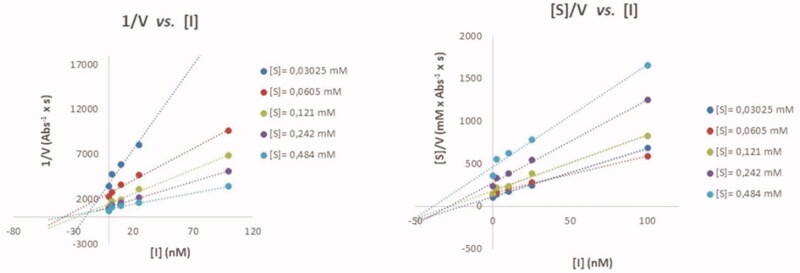
Cornish-Bowden plots for analysing the inhibition mode of *ee*AChE by **11b**.

The inhibition modes of *ee*AChE and eqBuChE by heterodimer **11b** were investigated by using the Cornish-Bowden method, that is, by creating two plots (1/V *vs.* [I] and [S]/V *vs.* [I]) for the inhibition of both enzymes (Figure 3). The two plots for the inhibition of each enzyme included a point of intersection at different [I]-coordinates, which implies that **11b** is a mixed inhibitor of both enzymes^47^. The competitive inhibition constant, *K*_i_, and uncompetitive inhibition constant, α*K*_i_, for *ee*AChE by **11b** is 19.0 ± 1.8 nM and 21.9 ± 7.2 nM, respectively. The inhibition constants of eqBuChE are *K*_i_ =10.0 ± 2.7 nM and α*K*_i_ = 14.3 ± 3.3 nM. The mixed inhibition modes of *ee*AChE and eqBuChE by **11b** were interpreted to indicate that **11b** behaves as a dual binding site inhibitor of both enzymes; it is tempting to think that **11b** binds simultaneously to the active site and PAS of both *ee*AChE and eqBuChE. However, in this context it is worth mentioning that the architecture of PAS in the two enzymes is different as it is richer on aromatic amino acid residues in *ee*AChE^48^^,^^49^, which allow formation of π–π interactions and cation–π interactions with ligands^50^.

## Modelling studies

**Figure 4.**
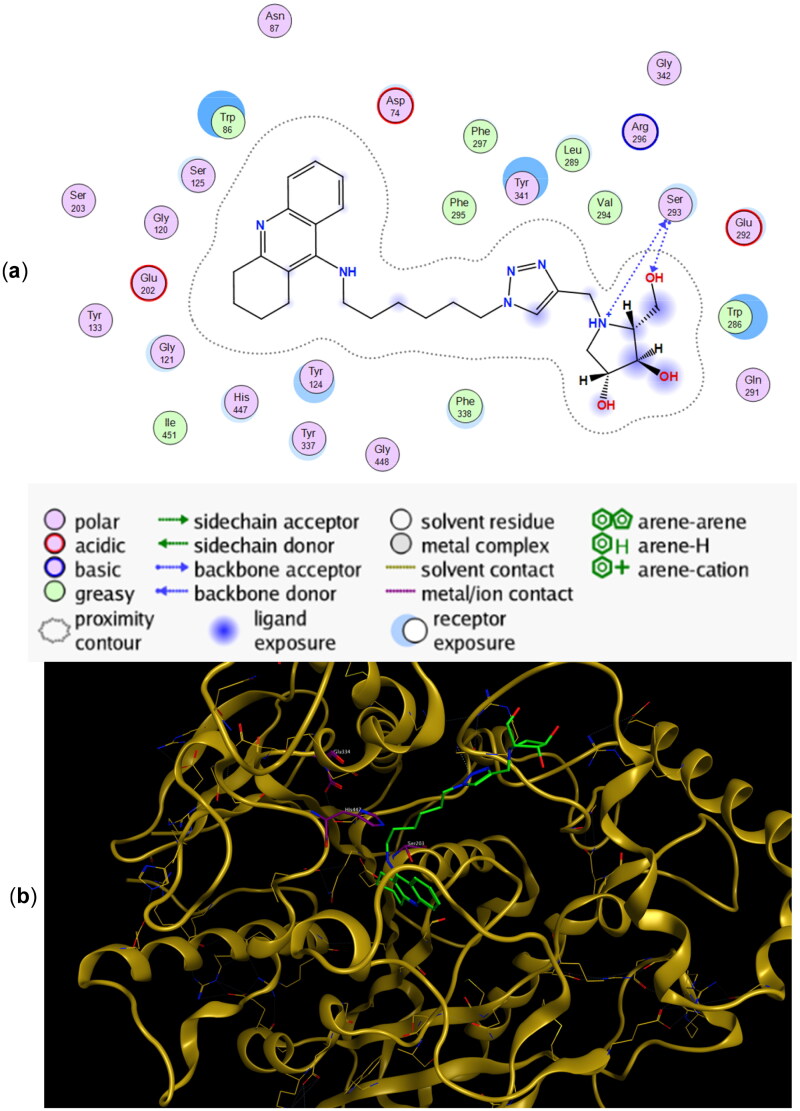
(a) Docking simulations for the interactions in the **11a**-*rh*AChE complex. (b) Three-dimensional structure of *rh*AChE showing the binding mode of compound **11a**. The residues, Ser203, His447, and Glu334 corresponding to the catalytic triad are depicted in sticks.

**Figure 5.**
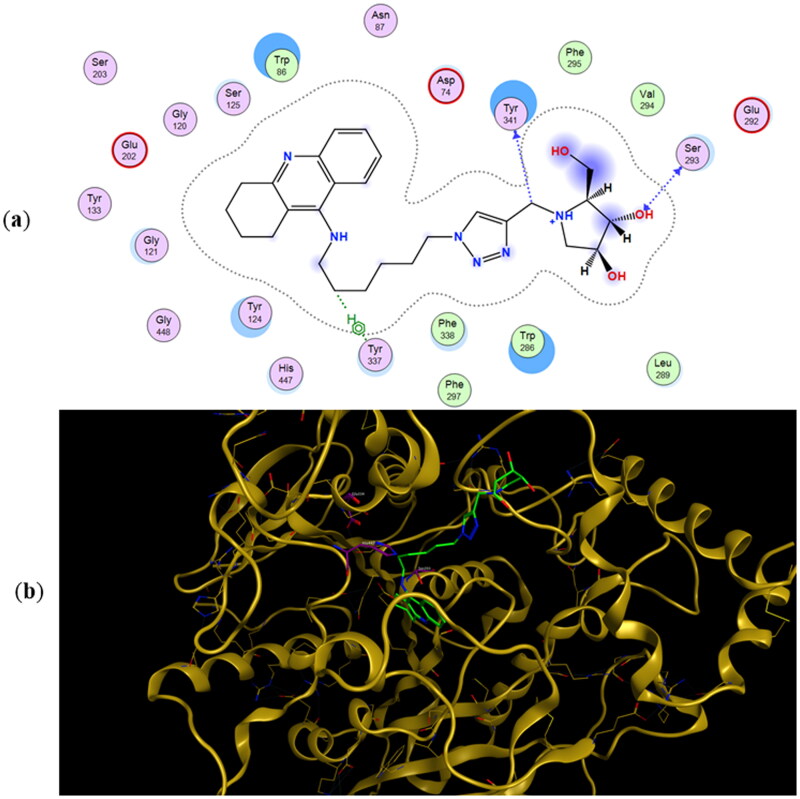
(a) Docking simulations for the interactions in the **11b**-*rh*AChE complex. (b) Three-dimensional structure of *rh*AChE showing the binding mode of compound **11b**. The residues, Ser203, His447, and Glu334 corresponding to the catalytic triad are depicted in sticks.

The preferred binding poses for enantiomers **11a** and **11b** in recombinant human acetylcholinesterase (*rh*AChE) are presented in Figure 4 and Figure 5, respectively, whereas the preferred binding poses for the enantiomeric pairs **9a** and **9b**, and **10a** and **10b** are presented in Figure SI2 and Figure SI3, respectively. A common trend for all energetically preferred binding poses is that the tacrine moiety and the iminosugar moiety bind to the active site and PAS, respectively. Such preferred binding pose is not very surprising given that X-ray analysis has shown that tacrine is bound to the active site of AChE^51^.

Hydrogen bonding interactions between one of the hydroxyl groups of the iminosugar moiety and Ser293 in *rh*AChE are observed in both **11a** (Figure 4) and **11b** (Figure 5). Interestingly, the protonated imino group of **11a** showed another hydrogen bond with the same Ser293, a feature not observed in **11b**. This helps explain the lower binding energy for **11a** (−10.45 kcal/mol) compared to its antipode **11b** (−9.92 kcal/mol) (Table 2). Slight differences in binding energies were also observed between enantiomers **9a** and **9b** (−9.25 kcal/mol for **9a** vs. −9.05 kcal/mol for **9b**) and between **10a** and **10b** (−9.52 kcal/mol for **10a** vs. −8.91 kcal/mol for **10b**) when they are bound to *rh*AChE. The hydroxyl groups of **9a** showed interactions with Tyr341 and Arg296 meanwhile there is an arene cation interaction between one of the hydroxyl groups in **9b** and Trp286 (Figure SI2). Hydrogen bond interaction between Ser293 and the iminosugar moiety is observed for **10a** but is lacking in its antipode **10b** (Figure SI3).

**Table 2.** Binding energies for **9a**, **9b**, **10a**, **10b**, **11a**, and **11b** to *rh*AChE and *h*BuChE.

	Binding energies (kcal/mol)	
Compound	*rh*AChE	*h*BuChE
**9a**	−9.25	−8.83
**9b**	−9.05	−8.94
**10a**	−9.52	−9.53
**10b**	−8.91	−9.44
**11a**	−10.45	−9.57
**11b**	−9.92	−9.67

We found that our measured IC_50_ values (Table 1) for the inhibition of *ee*AChE by **11a** and **11b** are in agreement with the calculated binding energies (Table 2), which predict **11a** and **11b** to possess the highest affinity for the enzyme. However, IC_50_ is not a true measure of binding affinity of a ligand to an enzyme^52^, which explains why the calculated binding energies in Table 2 fail in predicting the relative IC_50_ values for the inhibition of *ee*AChE by the heterodimers (**9a**–**11a** and **9b**–**11b**) in our series.

**Figure 6.**
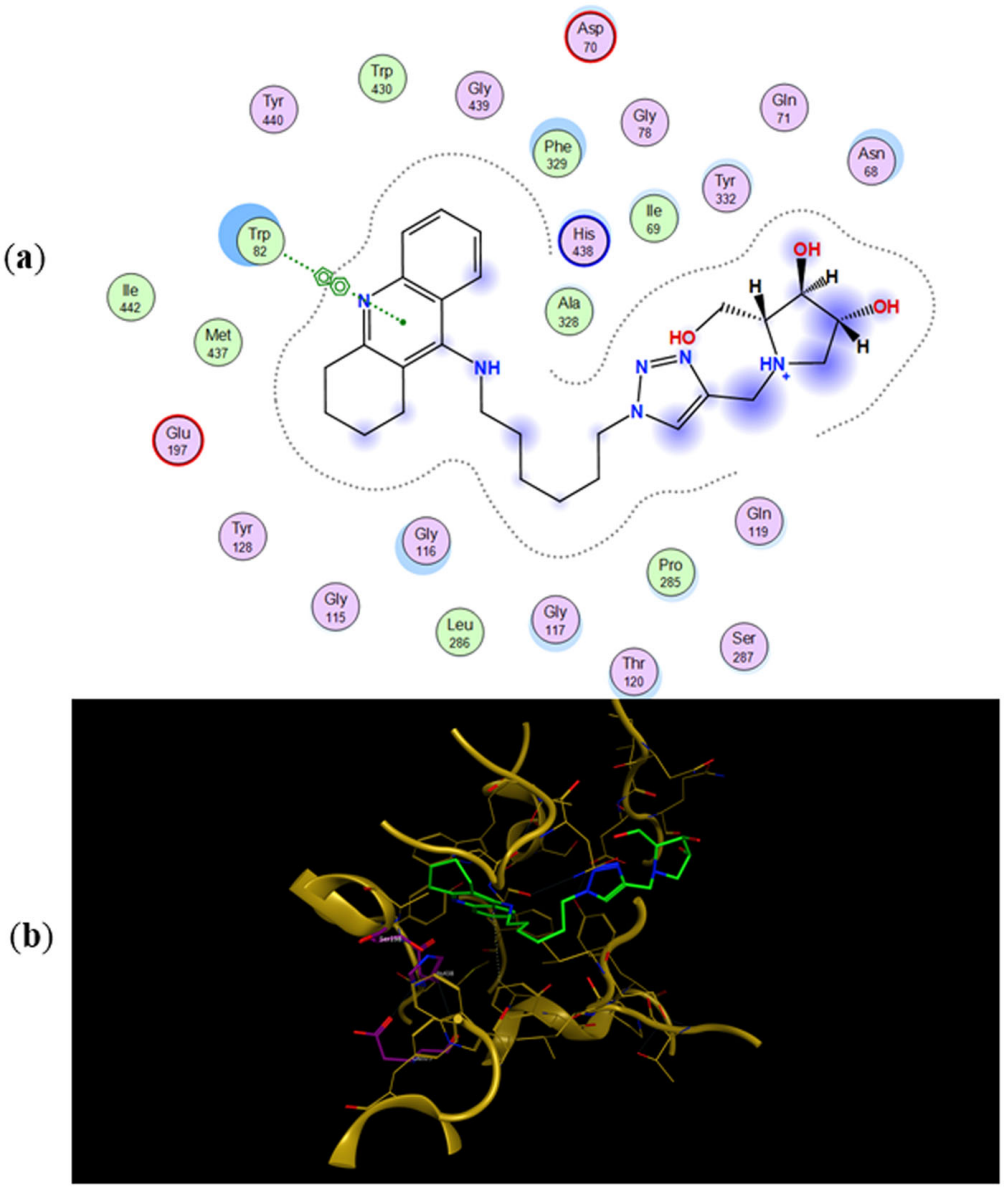
(a) Docking simulations for the interactions in the **11a**-*h*BuChE complex. (b) Three-dimensional structure of *h*BuChE showing the binding mode of compound **11a**. The residues, Ser198, His438 and Glu325, corresponding to the catalytic triad are depicted in sticks.

**Figure 7.**
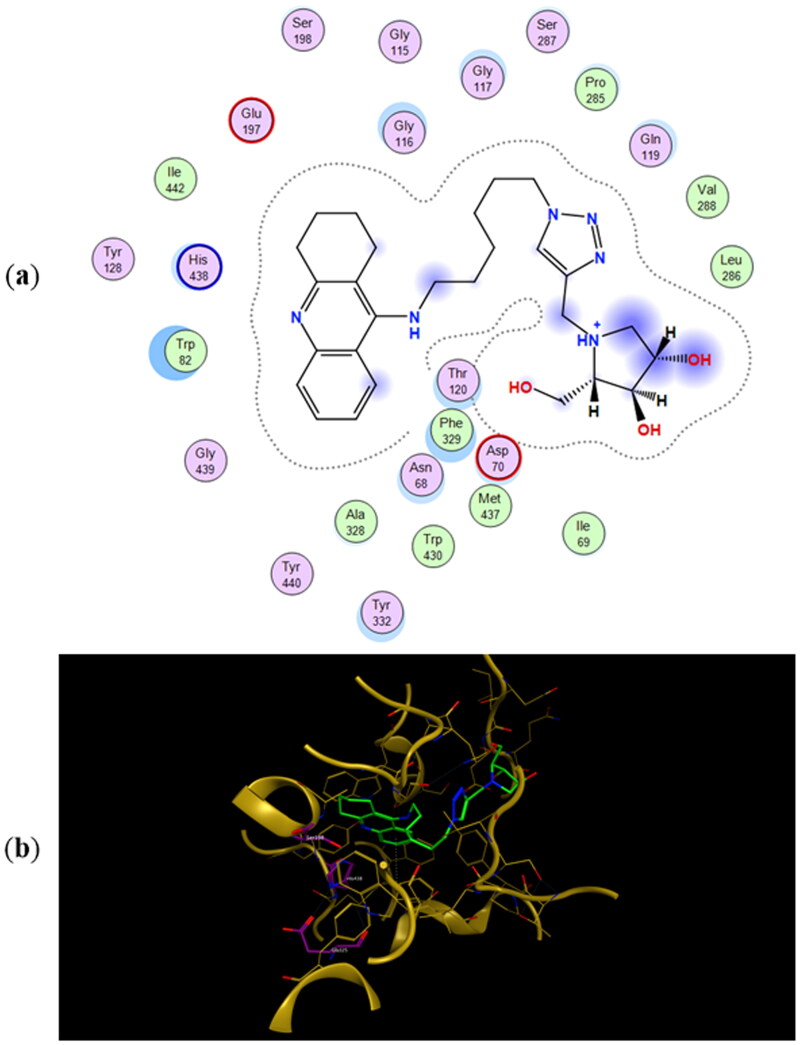
(a) Docking simulations for the interactions in the **11b**-*h*BuChE complex. (b) Three-dimensional structure of *h*BuChE showing the binding mode of compound **11b**. The residues, Ser198, His438, and Glu325, corresponding to the catalytic triad are depicted in sticks.

The most energetically favourable binding poses of enantiomers **11a** and **11b** to human butyrylcholinesterase (*h*BuChE) are presented in Figure 6 and Figure 7, respectively. The preferred binding poses of the enantiomeric pairs **9a** and **9b**, and **10a** and **10b** to the same enzyme are presented in Figures SI5 and SI6, respectively. The number of CH_2_-groups between the tacrine ring and 1,2,3-triazole ring appears to control whether the iminosugar moiety is bound to the active site or PAS. In fact, the tacrine ring of **9a**, **9b**, **11a**, and **11b** is accommodated in the active site whereas their iminosugar moiety is bound to PAS. For heterodimers **10a** and **10b** the binding scenarios are different, as the tacrine ring is accommodated in PAS and the iminosugar moiety in the active site. As for the inhibition of *ee*AChE, even though the calculated binding energies in Table 2 predict **11a** and **11b** to be the most potent BuChE inhibitors, they fail in predicting the relative IC_50_ values for the whole testing series.

## Antiproliferative activity

Antiproliferative activity of our heterodimers was investigated for six cancer cell lines including A549, HBL-100, HeLa, SW1573, T-47D and WiDr. The inhibition of cancer cell growth by each heterodimer is expressed in concentration of heterodimer required to lower the cell growth by 50% (GI_50_). The antiproliferative activity of a compound is only significant when GI_50_ ˂ 100 μM. The measured GI_50_ values demonstrated that those heterodimers with two CH_2_-groups (**9a** and **9b**) and three CH_2_-groups (**10a** and **10b**) between the tacrine and 1,2,3-triazole rings display no significant antiproliferative activity (GI_50_ > 100 μM). **11a** and **11b** on the other hand that contain six CH_2_-groups between the tacrine and 1,2,3-triazole rings display GI_50_ values below 100 μM for the inhibition of A549 cancer cell growth (Table 3). In addition, **11a** and **11b** display weak but significant inhibition of cell growth of HeLa and SW1573 cancer cells, respectively.

**Table 3.** Antiproliferative activity (GI_50_) of **9a**, **9b**, **10a**, **10b**, **11a**, and **11b** against human cancer cells.

	GI_50_ (μM)					
Compound	A549	HBL-100	HeLa	SW1573	T-47D	WiDr
**11a**	94 ± 9.6	>100	93 ± 12	>100	>100	>100
**11b**	84 ± 28	>100	>100	97 ± 5.4	>100	>100

## Conclusions

In contrast to the enantiomeric pairs **1a** and **1b** of huperzine, **2a** and **2b** of galantamine, and **3a** and **3b** of physostigmine, our enantiomeric pairs **9a** and **9b**, **10a** and **10b**, and **11a** and **11b** of iminosugar-tacrine heterodimers displayed low enantioselectivity (˂4) for the inhibition of *ee*AChE and eqBuChE. The following three observations: (1) **9a** is a *ca.* 3.5-fold stronger *ee*AChE inhibitor than **9b**, (2) **10a** is a *ca.* 3.5-fold less potent *ee*AChE inhibitor than **10b**, and (3) **11a** and **11b** are essentially equipotent *ee*AChE inhibitors, show that *ee*AChE exhibits no consequent preference for any of the enantiomeric heterodimers, which include a DAB or LAB moiety. These observations can either be interpreted as the tacrine moiety contributes much more to the *ee*AChE inhibitory potencies than the DAB or LAB moieties or that the LAB and DAB moieties have similar contribution to the inhibition potencies when they are connected to a tacrine ring. However, the latter interpretation is to some extent contradicted by the modelling studies, which show that the DAB and LAB moieties display different interaction modes with the enzymes.

Like in our earlier studies when we connected an iminosugar to a tacrine ring to obtain ChE inhibitors of type **7** in Figure 2^32^, heterodimers **11a** and **11b** with the longest linkers exhibited the highest inhibition potencies. From modelling studies for the binding to BuChE, it appeared that the length of the linker between the tacrine ring and DAB or LAB moiety controls whether the tacrine ring binds to the active site of PAS. On the other hand, because the trend of the measured IC_50_ values do not parallel the calculated binding energies, it is possible that the title compounds are not bound in their most energetically favourable poses when they inhibit the enzymes in our testing series.

## Supplementary Material

Supplemental Material
