# Supplementary material for: Investigation of the enantioselectivity of acetylcholinesterase and butyrylcholinesterase upon inhibition by tacrine-iminosugar heterodimers
Source: J Enzyme Inhib Med Chem. 2022 Dec 1;38(1):349–60. doi: 10.1080/14756366.2022.2150762 (PMC9721440; doi:10.1080/14756366.2022.2150762)

Investigation of the Enantioselectivity of Acetylcholinesterase and Butyrylcholinesterase upon Inhibition by Tacrine-Iminosugar Heterodimers

I. Caroline Vaaland,[a] Óscar López,[b*] Adrián Puerta,[c] Miguel X. Fernandes,[c] José M. Padrón,[c*] José G. Fernández-Bolaños,[b] Magne O. Sydnes,[a] Emil Lindbäck[a*]

[a]*Department of Chemistry, Bioscience and Environmental Engineering, Faculty of Science and Technology, University of Stavanger, NO-4036 Stavanger, Norway.*

[b]*Departamento de Química Orgánica, Facultad de Química, Universidad de Sevilla c/Profesor García González 1, 41012 Seville, Spain*

[c]*BioLab, Instituto Universitario de Bio-Orgánica "Antonio González" (IUBO-AG), Universidad de La Laguna, c/Astrofísico Francisco Sánchez 2, La Laguna, E-38206, Spain*

Table of content

Procedures 3
Compounds **15a** and **15b** 3
Compounds **16a** and **16b** 4
Compounds **17a** and **17b** 5
Compounds **18a** and **18b** 6
Compounds **19a** and **19b** 7
Compounds **20a-22a** and **20b-22b** 8
General procedure for the preparation of compounds **20a-22a** and **20b-22b** 8
Compounds **9a-11a** and **9b-11b** 13
General procedure for the preparation of compounds **9a-11a** and **9b-11b** 13
Modelling studies 17
Docking of **9a** and **9b** to *rh*AChE. 18
Docking of **10a** and **10b** to *rh*AChE 19
Docking of **11a** and **11b** to *rh*AChE. 20
Docking of **9a** and **9b** to *h*BuChE. 21
Docking of **10a** and **10b** to *h*BuChE. 22
Docking of **11a** and **11b** to *h*BuChE 23
References 24
NMR Spectrum 25

Procedures

Compounds **15a** and **15b**

2,3,5-Tri-*O*-benzyl-D-xylofuranose (**15b**)[1]

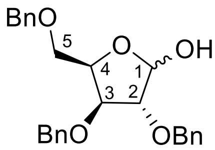

15b

The synthesis of **15b** was performed by following a reported 3-step procedure;[1] Compound **15b** (6.0 g, 72%) was obtained as a pale-yellow syrup and as mixture of anomers (5:3) from D-xylose (3.0 g, 20.0 mmol). R_f 0.25 (PE/EtOAc, 3:1); $[\alpha]_D^{26}$ +20 (*c* 0.8, MeOH); ^{1}H-NMR δ_H ($CDCl_3$, 400.13 MHz) 3.64-3.78 (2H, m, H-5a-major, H-5b-major, H-5a-minor-H-5b-minor), 3.84 (0.4H, d, *J* = 9.6 Hz, OH-minor), 3.91-3.94 (1H, m, OH-major, H-2-minor), 3.99-4.00 (0.6H, m, H-2-major), 4.03 (0.4H, dd, *J* = 2.4 Hz, J = 4.4 Hz, H-3-minor), 4.10 (0.6H, dd, *J* = 3.1 Hz, *J* = 5.5 Hz, H-3-major), 4.36-4.40 (1H, m, H-4-major, H-4-minor), 4.45-4.63 (6H, m, 6CHPh-major, 6CHPh-minor), 5.24 (0.6H, d, *J* = 11.6 Hz, H-1-major), 5.47 (0.4H, dd, *J* = 4.2 Hz, *J* = 9.6 Hz, H-1-minor), 7.23-7.36 (15H, m, 15ArH-major, 15Ar-minor); ^{13}C-NMR δC ($CDCl_3$, 100.61 MHz) 68.5 (C-5-minor), 68.9 (C-5-major), 72.0 (CH_2Ph-major), 72.4 (CH_2Ph-minor), 72.8 (CH_2Ph-major), 73.2 (CH_2Ph-minor), 73.6 (CH_2Ph-minor), 73.8 (CH_2Ph-major), 77.5 (C-4-minor), 80.0 (C-4-major), 81.1 (C-3-minor), 81.4 (C-3-major, C-2-minor), 86.7 (C-2-major), 96.3 (C-1-minor), 101.8 (C-1-major), 127.7-128.8 (9Ar-major, 9Ar-minor), 136.9 (Ar-minor), 137.5 (Ar-major), 137.6 (Ar-major), 137.7 (Ar-major), 137.8 (Ar-minor), 138.3 (Ar-minor).

2,3,5-Tri-*O*-benzyl-L-xylofuranose (**15a**)

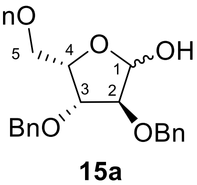

15a

Compound **15a** was prepared from L-xylose by following the same procedure as for **15b**. Compound **15a** (5.04 g, 61%) was obtained as a pale-yellow syrup and as mixture of epimers (5:3) from L-xylose (3.0 g, 20.0 mmol). $[\alpha]_D^{26}$ -9 (*c* 0.7, MeOH). The NMR data is in agreement with that of for **15b**.

Compounds **16a** and **16b**

2,3,5-Tri-*O*-benzyl-4-methanesulfonyl-D-xylose *O*-(*tert*-butyldiphenylsilyl) oxime (**16b**)[2]

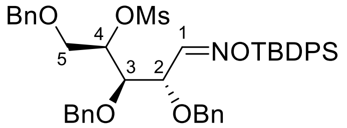

16b

To a solution of 2,3,5-tri-*O*-benzyl-D-xylofuranose (**15b**) (5.72 g, 13.6 mmol, 1 equiv.) and hydroxylamine hydrochloride (7.56 g, 0.11 mol, 8 equiv.) in MeOH (60 mL) at room temperature was added sodium methoxide (10.1 mL, 5.4 M in MeOH, 54.4 mmol, 4 equiv.). After addition, the mixture was kept stirring for 2 hours before the solvent was removed under reduced pressure. CH_2Cl_2 (50 mL) was added to the residue and the organic layer was washed with water (3 x 50 mL), dried ($MgSO_4$), filtered, and concentrated *in vacuo* to obtain crude oxime, 2,3,5-tri-*O*-benzyl-D-xylose oxime, as a pale-yellow oil.

The crude oxime and imidazole (1.2 g, 17.7 mmol, 1.3 equiv.) were dissolved in dry CH_2Cl_2 (25 mL) under an Ar-atmosphere. TBDPS-Cl (4.42 mL, 17 mmol, 1.25 equiv.) was added slowly at 0 °C and then the mixture was kept stirring at room temperature for 30 minutes. The mixture was added water/CH_2Cl_2 (50 mL, 1:1 v/v) and the phases were separated. The aqueous layer was extracted with CH_2Cl_2 (3 x 50 mL). The combined organic extracts were washed with brine, dried ($MgSO_4$), filtered, and concentrated under reduced pressure to obtain a crude of an *O*-TBDPS protected oxime.

The crude *O*-TBDPS protected oxime and triethylamine (3 mL, 21.8 mmol, 1.6 equiv.) were dissolved in dry CH_2Cl_2 (43 mL) under an Ar-atmosphere. MsCl (1.58 mL, 20.4 mmol, 1.5 equiv.) was added slowly at 0 ˚C and then the mixture was kept stirring for 30 minutes at 0 ˚C. After this time, water (15 mL) was added and the aqueous mixture was extracted with (3 x 50 mL). The combined organic extracts were dried ($MgSO_4$), filtered, and concentrated under reduced pressure. The residue underwent purification by silica gel flash column chromatography (PE/EtOAc, 16:4 → 13:7) to provide the title compound **16b** as a blank colorless syrup (5.7 g, 56%); R_f 0.23 (PE/EtOAc, 1.7:0.3); $[\alpha]_D^{26}$ +37 (c 0.6, MeOH); Very complex NMR spectrum due to mixture (4/1) of *E* and *Z* isomers: ^{1}H-NMR δ_H ($CDCl_3$, 400.13 MHz) 1.10 (9H, s, ^{t}Bu-major, ^{t}Bu-minor), 2.89 (0.6H, s, CH_3-minor), 2.91 (2.4H, s, CH_3-major), 3.37 (0.8H, dd, *J* = 5.4 Hz, *J* = 11.6 Hz, H-5b-major), 3.46 (0.2H, dd, *J* = 5.4 Hz, *J* = 11.6 Hz, H-5b-minor), 3.57 (0.8H, dd, *J* = 3.1 Hz, *J* = 11.6 Hz, H-5a-major), 3.61 (0.2H, dd, *J* = 2.7 Hz, *J* = 11.6 Hz, H-5a-minor), 3.87 (0.8H, dd, *J* = 4.7 Hz, *J* = 6.2 Hz, H-3-major), 4.02 (0.8H, dd, *J* = 4.7 Hz, *J* = 7.6 Hz, H-2-major), 4.07 (0.8H, d, *J* = 12.0 Hz, CHPh-major), 4.18 (0.8H, d, *J* = 11.7 Hz, CHPh-major), 4.21-4.24 (0.2H, m, H-3-minor), 4.32-4.63 (4.4H, m, 4CHPh-major, 6CHPh-minor), 4.87-4.91 (0.8H, m, H-4-major), 4.91-4.95 (0.2H, m, H-4-minor), 5.05 (0.2H, dd, *J* = 3.0 Hz, *J* = 5.0 Hz, H-2-minor), 7.07-7.10 (1.6H, m, ArH), 7.16-7.42 (19.4H, m, H-1-minor, ArH), 7.67-7.72 (5H, m, H-1-major); HRMS (ESI): *m/z* $[M + Na]^+$ calcd. for $C_{43}H_{49}NO_7SSiNa$: 774.2891; found: 774.2895.

2,3,5-Tri-*O*-benzyl-4-methanesulfonyl-L-xylose *O*-(*tert*-butyldiphenylsilyl) oxime (**16a**)

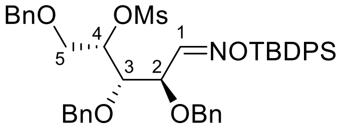

16a

Compound **16a** was prepared from methyl 2,3,5-tri-*O*-benzyl-L-xylofuranose **15a** following the same procedure as for **16b**. Compound **16a** (6.49 g, 78%) was obtained as a colorless syrup and as a mixture of (4/1) of E and Z isomers. R_f 0.24 (PE/EtOAc, 1.7:0.3); $[\alpha]_D^{26}$ -17 (c 0.8, MeOH). HRMS (ESI): *m/z* $[M + Na]^+$ calcd. for $C_{43}H_{49}NO_7SSiNa$: 774.2891; found: 774.2897. The ^{1}H-NMR data is in agreement with that for **16b**.

Compounds **17a** and **17b**

1,4-Anhydro-2,3,5-tri-*O*-benzyl-1-deoxy-1-imino-D-arabinitol *N*-oxide (**17a**)[3]

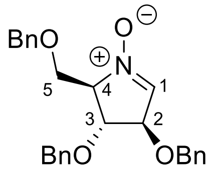

17a

A solution of 2,3,5-tri-*O*-benzyl-4-methanesulfonyl-L-xylose *O*-(*tert*-butyldiphenylsilyl) oxime (**16a**) (5.2 g, 6.9 mmol, 1 equiv.) in toluene (80 mL) under an Ar-atmosphere at room temperature was slowly added tetrabutylammonium fluoride (11 mL, 1 M in THF, 11 mmol, 1.55 equiv.). The mixture was refluxed for 45 minutes. After this time, the mixture was allowed to reach ambient temperature before the volatiles were removed under reduced pressure. The residue was dissolved in CH_2Cl_2 (50 mL), and water (100 mL) and the phases were separated. The aqueous phase was extracted with CH_2Cl_2 (3 x 50 mL). The combined organic extracts were washed with brine (30 mL) and the solvent was removed under reduced pressure. Purification by flash column chromatography (PE/EtOAc, 1:1 → 0:1) gave the title compound **17a** (1.99 g, 69%) as a brown wax; R_f 0.32 (PE/EtOAc, 0.65:0.35); $[\alpha]_D^{26}$ -33 (*c* 0.6, $CHCl_3$); ^{1}H-NMR δ_H ($CDCl_3$, 400.13 MHz) 3.77 (1H, dd, *J* = 2.8 Hz, *J* = 9.9 Hz, H-5a), 4.00-4.06 (2H, m, H-4, H-5b), 4.37 (1H, dd, *J* = 2.3 Hz, *J* = 3.5 Hz, H-3), 4.49-4.55 (5H, m, 5CHPh), 4.61 (1H, d, *J* = 12.0 Hz, CHPh), 4.66 (1H, t, *J* =2.1 Hz, H-2), 6.88 (1H, brs, H-1), 7.25-7.36 (15H, m, 15ArH); ^{13}C-NMR δC ($CDCl_3$, 100.61 MHz) 66.2 (C-5), 71.8, 72.0, 73.6 ($3CH_2Ph$), 77.6 (C-4), 80.5 (C-3), 82.9 (C-2), 127.8-128.8 (Ar), 137.2 (Ar), 137.3 (Ar), 137.8 (Ar). The NMR data is in agreement with reported data.[2]

1,4-Anhydro-2,3,5-tri-*O*-benzyl-1-deoxy-1-imino-L-arabinitol *N*-oxide (**17b**)

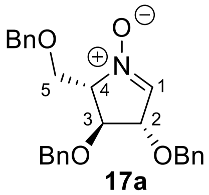

17a

Compound **17b** was prepared from 2,3,5-tri-*O*-benzyl-4-methanesulfonyl-D-xylose *O*-(*tert*-butyldiphenylsilyl) oxime (**16b**) using the same procedure as for **17a**. Compound **17b** (1.39 g, 46%) was obtained as a brown wax; R_f 0.32 (PE/EtOAc, 0.65:0.35); $[\alpha]_D^{27}$ +74 (c 0.2, $CHCl_3$). The NMR data is in agreement with that for **17a**.

Compounds **18a** and **18b**

2,3,5-Tri-*O*-benzyl-1,4-dideoxy-1,4-imino-L-arabinitol (**18b**)[4]

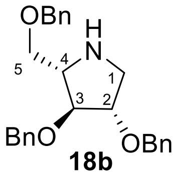

18b

The synthesis of **18b** from 1,4-anhydro-2,3,5-tri-*O*-benzyl-1-deoxy-1-imino-L-arabinitol *N*-oxide (**17b**) (1.31 g, 3.14 mmol, 1 equiv.) was performed by following a reported procedure.[4] Compound **18b** was purified by flash column chromatography (CH_2Cl_2/MeOH, 100:0 → 94:6) affording title compound (0.85 g, 67%) as a pale yellow oil; R_f 0.13 (CH_2Cl_2/MeOH, 30:1); $[\alpha]_D^{26}$ -9 (c 0.7, $CHCl_3$). ^{1}H-NMR δ_H ($CDCl_3$, 400.13 MHz) 2.06 (1H, brs, NH), 3.09 (2H, d, *J*= 3.82 Hz, H-1), 3.24 (1H, q, *J* = 5.4 Hz, H-4), 3.55 (1H, dd, *J* = 9.5, *J* = 5.6 Hz, H-5b), 3.61 (1H, dd, *J* = 9.5 Hz, *J* = 5.2 Hz, H-5a), 3.87 (1H, dd, *J* =4.6 Hz, *J* =1.6 Hz, *J* =1.7 Hz, H-3), 4.01 (1H, m, H-2), 4.50 (6H, m, 3CH_2Ph), 7.28-7.32 (15H, m, 15ArH); ^{13}C-NMR δC ($CDCl_3$, 100.61 MHz) 51.3 (C-1), 64.3 (C-4), 70.6 (C-5), 71.2, 72.0, 73.3 (3CH_2Ph), 84.7 (C-2), 85.9 (C-3), 127.7-128.5 (Ar), 138.3 (Ar), 138.3 (Ar), 138.4 (Ar). The NMR data is in agreement with reported data.[5]

2,3,5-Tri-*O*-benzyl-1,4-dideoxy-1,4-imino-D-arabinitol (**18a**)

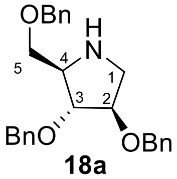

18a

Compound **18** was prepared from 1,4-anhydro-2,3,5-tri-*O*-benzyl-1-deoxy-1-imino-D-arabinitol *N*-Oxide (**17a**) using the same procedure as for **18b**. Compound **18a** (1.47 g, 82%) was obtained as a pale-yellow oil. R_f 0.13 (CH_2Cl_2/MeOH, 30:1); $[\alpha]_D^{26}$ +14 (c 0.3, $CHCl_3$). The NMR data is in agreement with that for **18b**.

Compounds **19a** and **19b**

N-Propagyl-2,3,5-tri-*O*-benzyl-1,4-dideoxy-1,4-imino-D-arabinitol (**19a**)

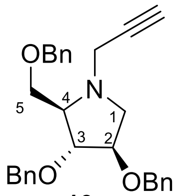

19a

To a stirred solution of 2,3,5-tri-*O*-benzyl-1,4-dideoxy-1,4-imino-D-arabinitol (**18a**) (0.42 g, 1.052 mmol, 1 equiv.) in acetone (10 ml), K_2CO_3 (0.36 g, 2.6 mmol, 2.6 equiv.) under an Ar-atmosphere, propargyl bromide (0.293 mL, 0.39 g, 2.5 mmol, 2.5 equiv.) was added. The resultant mixture was refluxed for 4 hours before the solvent was removed under reduced pressure. Water was added (40 mL) to the residue and the resultant aqueous mixture was extracted with CH_2Cl_2 (3 x 40 mL). The organic layer was washed with brine, dried ($MgSO_4$), and concentrated *in vacuo*. The crude product was purified by silica gel column chromatography (PE/EtOAc, 95:5 → 80:20) to obtain the title compound **19a** as yellow syrup (0.26 g, 56%); R_f 0.32 (PE:EtOAc, 3:1); $[\alpha]_D^{26}$ -33 (c 0.43, $CHCl_3$). ^{1}H-NMR δ_H ($CDCl_3$, 400.13 MHz) 2.20 (1H, t, *J* = 2.4 Hz, ≡C−H), 2.96-2.98 (1H, m, H-4), 2.99-3.00 (1H, m, H-1a), 3.07 (1H, d, *J* = 10.4 Hz, H-1b), 3.54-3.62 (3H, m, NCH_2, H-5), 3.67 (1H, dd, *J* = 17.5 Hz, *J* = 2.3 Hz, NCH_2), 3.84-3.85 (1H, m, H-3), 3.91-3.93 (1H, m, H-2), 4.42-4.56 (6H, m, 3xCH_2Ph), 7.26-7.33 (15H, m, 15ArH); ^{13}C-NMR δC ($CDCl_3$, 100.61 MHz) 41.5 (NCH_2), 56.0 (C-1), 65.7 (C-4), 70.7 (C-5), 71.3, 71.6, 73.2 (3xCH_2Ph), 73.5 (≡C−H), 78.6 (C≡), 81.5 (C-2), 85.8 (C-3), 127.7-128.5 (Ar), 138.2 (Ar), 138.3 (Ar), 138.4 (Ar); HRMS (ESI): *m/z* $[M + H]^+$ calcd. for $C_{29}H_{32}NO_3$: 442.2377; found: 442.2373.

N-Propagyl-2,3,5-tri-*O*-benzyl-1,4-dideoxy-1,4-imino-L-arabinitol (**19b**)

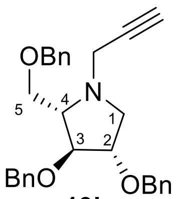

19b

Compound **19b** was prepared from 2,3,5-tri-*O*-benzyl-1,4-dideoxy-1,4-imino-L-arabinitol (**18b**) using the same procedure as for **19a**. Compound **19b** was obtained as yellow syrup (0.35 g, 47%); R_f 0.32 (PE:EtOAc, 3:1); $[\alpha]_D^{26}$ +32 (*c* 0.82, $CHCl_3$); The NMR data is in agreement with that for **19a**; HRMS (ESI): *m/z* $[M + H]^+$ calcd. for $C_{29}H_{32}NO_3$: 442.2377; found: 442.2372.

Compounds **20a-22a** and **20b-22b**

General procedure for the preparation of compounds **20a-22a** and **20b-22b**

A mixture of **19b** (1 equiv., 0.04 M for synthesis of **20b**, **21b**, and **22b**) or **19a** (1 equiv., 0.07 M for synthesis of **20a**, 0.04 M for synthesis of **21a**, and 0.05 M for synthesis of **22a**), azide **13** (0.98 equiv.), and copper(II) sulfate pentahydrate (0.30 equiv.) in anhydrous DMF in an aluminum foil covered round bottom flask was degassed and introduced an argon atmosphere before the addition of sodium ascorbate (0.60 equiv.). After addition, the mixture was kept stirring for 48 h at rt. The solvent was then removed under reduced pressure and the residue obtained was purified by silica gel column chromatography

N-((1-(2-((1,2,3,4-Tetrahydroacridin-9-yl)amino)ethyl)-1*H*-1,2,3-triazol-4-yl)methyl)-2,3,5-tri-*O*-benzyl-1,4-dideoxy-1,4-imino-L-arabinitol (**20b**)

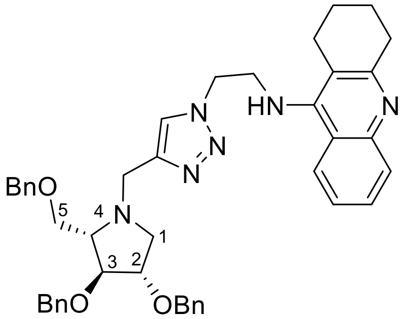

20b

The crude product of **20b** was purified by silica gel flash column chromatography (CH_2Cl_2/MeOH/NH_4OH, 190:10:1 → 180:20:1) to obtain the title compound **20b** (65 mg, 82%) as a brown oil. R_f 0.37 (CH_2Cl_2/MeOH/NH_4OH, 9:1:0.1); $[\alpha]_D^{26}$ +17 (*c* 0.7, $CHCl_3$); ^{1}H-NMR δ_H ($CDCl_3$, 400.13 MHz) 1.84-1.86 (4H, m, 2CH_2), 2.58-2.61 (2H, m, CH_2), 2.71-2.80 (2H, m, H-1b, H-4), 3.03-3.11 (2H, m, CH_2), 3.10 (1H, d, *J* = 10.7 Hz, H-1a), 3.63-3.64 (2H, m, H-5a, H-5b), 3.78-3.79 (1H, m, H-3), 3.85-3.86 (1H, m, H-2), 3.92-3.95 (3H, m, CHbN, CH_2), 4.15 (1H, d, *J* = 14.5 Hz, CHaN), 4.36-4.50 (8H, m, 6CHPh, CH_2), 4.71 (1H, brs, NH), 7.20-7.37 (16H, m, ArH), 7.42 (1H, s, ArH), 7.51-7.55 (1H, m, ArH), 7.77 (1H, d, *J* = 8.1, ArH), 7.94 (1H, d, *J* = 8.1, ArH); ^{13}C-NMR δC ($CDCl_3$, 100.61 MHz) 22.6 (CH_2), 22.9 (CH_2), 24.8 (CH_2), 33.5 (CH_2), 48.0 (2CH_2), 50.6 (CH_2), 56.6 (C-1) 67.1 (C-4), 71.2 (CH_2Ph), 71.7 (C-5, CH_2Ph), 73.4 (CH_2Ph), 81.3 (C-2), 85.8 (C-3), 117.7 (Ar), 120.3 (Ar), 122.4 (Ar), 124.2 (Ar), 124.6 (Ar), 127.8-129.0 (11Ar), 138.1 (2Ar), 138.3 (Ar), 144.2 (Ar), 146.5 (Ar), 149.9 (Ar), 158.3 (Ar); HRMS (ESI): *m/z* $[M + H]^+$ calcd. for $C_{44}H_{49}N_6O_3$: 709.3861; found: 709.3856.

N-((1-(2-((1,2,3,4-Tetrahydroacridin-9-yl)amino)ethyl)-1*H*-1,2,3-triazol-4-yl)methyl)-2,3,5-tri-*O*-benzyl-1,4-dideoxy-1,4-imino-D-arabinitol (**20a**)

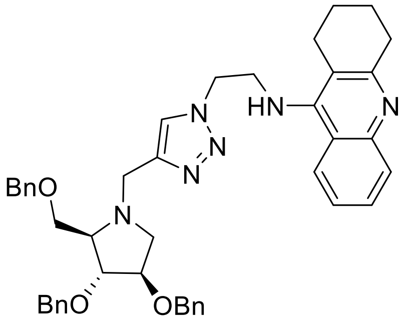

20a

The crude product was purified by silica gel flash column chromatography (CH_2Cl_2/MeOH/NH_4OH, 95:5:1) to obtain the title compound **20a** (110 mg, 76%) as a brown oil. R_f 0.37 (CH_2Cl_2/MeOH/NH_4OH, 9:1:0.1); $[\alpha]_D^{26}$ -18 (*c* 0.9, $CHCl_3$); The NMR data is in agreement for that of **20b**; HRMS (ESI): *m/z* $[M + H]^+$ calcd. for $C_{44}H_{49}N_6O_3$: 709.3861; found: 709.3854.

N-((1-(2-((1,2,3,4-Tetrahydroacridin-9-yl)amino)propyl)-1*H*-1,2,3-triazol-4-yl)methyl)-2,3,5-tri-*O*-benzyl-1,4-dideoxy-1,4-imino-L-arabinitol (**21b**)

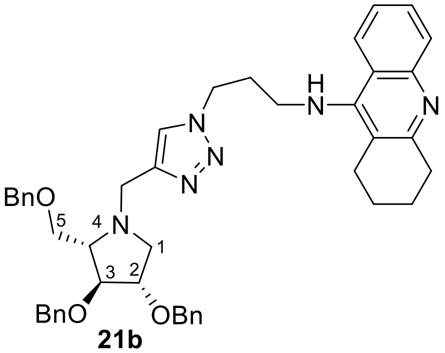

21b

The crude product was purified by silica gel flash column chromatography (CH_2Cl_2/MeOH/NH_4OH, 190:10:1 → 180:20:1) to obtain the title compound **21b** (49.8 mg, 59%) as a brown oil. R_f 0.24 (CH_2Cl_2/MeOH/NH_4OH, 9:1:0.1); $[\alpha]_D^{26}$ +8 (*c* 0.5, $CHCl_3$); ^{1}H-NMR δ_H ($CDCl_3$, 400.13 MHz) 1.90 (4H, brs, 2CH_2), 2.14-2.18 (2H, m, CH_2), 2.70 (2H, brs, CH_2), 2.73-2.81 (2H, m, H-1b, H-4), 3.11-3.13 (3H, m, H-1a, CH_2), 3.47 (2H, brs, CH_2), 3.64-3.66 (2H, m, H-5a, H-5b), 3.78-3.80 (1H, m, H-3), 3.85-3.86 (1H, m, H-2), 3.95 (1H, d, *J* = 14.6 Hz, CHbN), 4.16 (1H, d, *J* = 14.6 Hz, CHaN), 4.37-4.52 (8H, m, CH_2, 6CHPh), 7.27-7.31 (15H, m, ArH), 7.36-7.40 (1H, m, ArH), 7.41 (1H, s, ArH), 7.57-7.60 (1H, m, ArH), 7.92 (1H, d, *J* = 8.1, ArH), 8.03 (1H, brs, ArH); ^{13}C-NMR δC ($CDCl_3$, 100.61 MHz) 22.5 (CH_2), 22.9 (CH_2), 24.9 (CH_2), 31.4 (CH_2), 33.2 (CH_2, visible in HSQC), 45.3 (CH_2), 47.5 (CH_2), 48.0 (CH_2), 56.6 (C-1), 67.0 (C-4), 71.3, 71.7, 71.8 (2CH2Ph, C-5), 73.5 (CH_2Ph), 81.3 (C-2), 85.9 (C-3), 122.5 (Ar), 123.5 (Ar), 124.7 (Ar), 127.8-129.4 (11Ar), 138.1 (Ar), 138.2 (Ar), 138.4 (Ar), 144.3 (Ar) (five signals are obscured in the ^{13}C-NMR spectrum); HRMS (ESI): *m/z* $[M + H]^+$ calcd. for $C_{45}H_{51}N_6O_3$: 723.4017; found: 723.4010.

N-((1-(2-((1,2,3,4-Tetrahydroacridin-9-yl)amino)propyl)-1*H*-1,2,3-triazol-4-yl)methyl)-2,3,5-tri-*O*-benzyl-1,4-dideoxy-1,4-imino-D-arabinitol (**21a**)

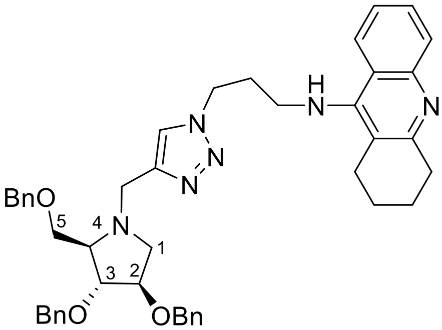

21a

The crude product was purified by silica gel flash column chromatography (CH_2Cl_2/MeOH/NH_4OH, 95:5:1 → 85:15:1) to obtain the title compound **21a** (54.2 mg, 73%) as a brown oil. R_f 0.23 (CH_2Cl_2/MeOH/NH_4OH, 9:1:0.1); $[\alpha]_D^{26}$ -25 (*c* 0.5, $CHCl_3$); The NMR data is in agreement with that for **21b**; HRMS (ESI): *m/z* $[M + H]^+$ calcd. for $C_{45}H_{51}N_6O_3$: 723.4017; found: 723.4013.

N-((1-(2-((1,2,3,4-Tetrahydroacridin-9-yl)amino)hexyl)-1*H*-1,2,3-triazol-4-yl)methyl)-2,3,5-tri-*O*-benzyl-1,4-dideoxy-1,4-imino-D-arabinitol (**22a**)

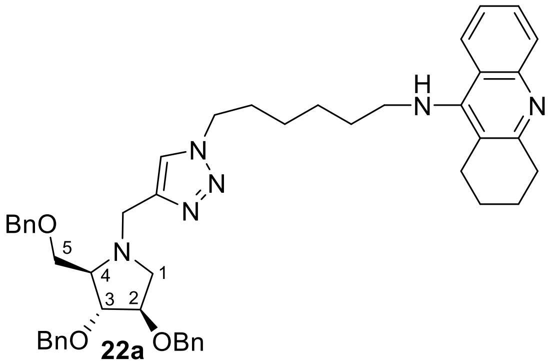

The crude product was purified by silica gel flash column chromatography (CH_2Cl_2/MeOH/NH_4OH, 95:5:1 → 90:10:1) to obtain the title compound **22a** (88.8 mg, 55%) as a brown oil. R_f 0.29 (CH_2Cl_2/MeOH/NH_4OH, 9:1:0.1); $[\alpha]_D^{26}$ -24 (*c* 0.8, $CHCl_3$); ^{1}H-NMR δ_H ($CDCl_3$, 400.13 MHz) 1.25-1.34 (2H, m, CH_2), 1.36-1.43(2H, m, CH_2), 1.59-1.61 (2H, m, CH_2), 1.79-1.86 (2H, m, CH_2), 1.91-1.92 (4H, m, 2CH_2), 2.69-2.70 (2H, m, CH_2), 2.73-2.80 (2H, m, H-1b, H-4), 3.07 (2H, brs, CH_2), 3.12 (1H, d, *J* = 11.1 Hz, H-1a), 3.45 (2H, t, *J* = 7.2 Hz, CH_2), 3.64-3.65 (2H, m, H-5a, H-5b), 3.80 (1H, d, *J* = 5.1 Hz, H-3), 3.85-3.86 (1H, m, H-2), 3.93 (1H, d, *J* = 14.5 Hz, CHbN), 4.14 (1H, d, *J* = 14.5 Hz, CHaN), 4.21-4.25 (2H, m, CH_2), 4.38 (1H, d, *J* = 12.2 Hz, CHPh), 4.42-4.52 (5H, m, 5CHPh), 7.21-7.36 (16H, m, 16ArH), 7.39 (1H, s, ArH), 7.53-7.57 (1H, m, ArH), 7.92-7.94 (2H, m, 2ArH); ^{13}C-NMR δC (D_2O 100.61 MHz) 22.7 (CH_2), 23.0 (CH_2), 24.8 (CH_2), 26.3 (2CH_2), 30.1 (CH_2), 31.5 (CH_2), 33.8 (CH_2), 48.0 (CH_2), 49.2 (CH_2), 49.9 (CH_2), 56.4 (C-1), 66.9 (C-4), 71.1 (CH_2Ph), 71.6 (C-5, CH_2Ph), 73.4 (CH_2Ph), 81.2 (C-2), 85.8 (C-3), 115.9 (Ar), 120.1 (Ar), 122.8 (Ar), 122.9 (Ar), 123.8 (Ar), 127.6-128.5

(11Ar), 138.0 (Ar), 138.1 (Ar), 138.3 (Ar), 143.7 (Ar), 147.2 (Ar), 150.7 (Ar), 158.3 (Ar); HRMS (ESI): *m/z* [M + H]$^+$ calcd. for $C_{48}H_{57}N_6O_3$: 765.4487; found: 765.4477.

N-((1-(2-((1,2,3,4-Tetrahydroacridin-9-yl)amino)hexyl)-1*H*-1,2,3-triazol-4-yl)methyl)-2,3,5-tri-*O*-benzyl-1,4-dideoxy-1,4-imino-L-arabinitol (**22b**)

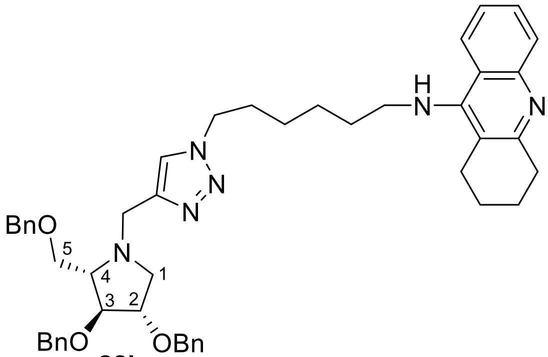

22b

The crude product was purified by silica gel flash column chromatography (CH_2Cl_2/MeOH/NH_4OH, 190:10:1 → 180:20:1) to obtain the title compound **22b** (60.1 mg, 72%) as a brown oil, which was pure enough for going ahead. R_f 0.26 (CH_2Cl_2/MeOH/NH_4OH, 9:1:0.1); NMR data is in agreement with that of **22a**; HRMS (ESI): *m/z* [M + H]$^+$ calcd. for $C_{48}H_{57}N_6O_3$: 765.4487; found: 765.4477.

Compounds **9a-11a** and **9b-11b**

General procedure for the preparation of compounds **9a-11a** and **9b-11**

To a mixture of **20a-22a** (0.02 M, 1 equiv.) or **20b-22b** (0.02 M, 1 equiv.) in anhydrous CH_2Cl_2 under an argon atmosphere at -78 ˚C was slowly added BCl_3 (1 M in heptane, 15 equiv.). After addition, the mixture was kept stirring at -78 ˚C for 2 h and then at 0 ˚C overnight. The volatiles were then removed under reduced pressure and the concentrate underwent purification by silica gel chromatography ($MeCN/H_2O/NH_4OH$, 190:10:1 → 180:20:1) (column 1). The corresponding HCl salt was dissolved in MeOH (2 mL) and NH_4OH (0.5 mL) and kept stirring for 48 hours. The solvent was removed under reduced pressure and the resulting residue was purified by silica gel chromatography (column 2).

N-((1-(2-((1,2,3,4-Tetrahydroacridin-9-yl)amino)ethyl)-1*H*-1,2,3-triazol-4-yl)methyl)-1,4-dideoxy-1,4-imino-L-arabinitol (**9b**)

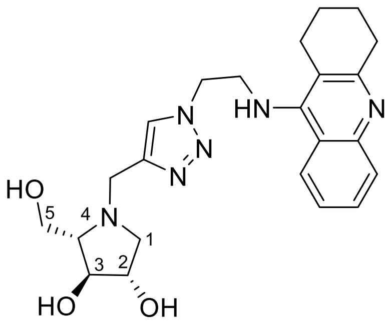

9b

The crude product was purified by silica gel flash column chromatography ($MeCN/H_2O/NH_4OH$, 190:10:1 → 180:20:1) (column 2) to obtain the title compound **9b** (21.7 mg, 74%) as a colorless oil. R_f 0.19 ($MeCN/H_2O/NH_4OH$, 15:5:0.2); $[\alpha]_D^{26}$ +11 (*c* 0.5, MeOH); ^{1}H-NMR δ_H (D_2O, 400.13 MHz) 1.79 (4H, brs, $2CH_2$), 2.35-2.36 (3H, m, H-4, CH_2), 2.44-2.52 (2H, m, H-1a. H-1b), 2.77 (2H, brs, CH_2), 3.39 (1H, d, *J* = 14.2 Hz, CHbN), 3.54-3.67 (3H, m, CHaN, H-5a, H-5b), 3.80-3.81 (1H, m, H-3), 3.84-3.86 (1H, m, H-2), 4.22 (2H, t, *J* = 5.0 Hz, CH_2), 4.58 (2H, t, *J* = 5.0 Hz, CH_2), 7.39-7.42 (1H, m, ArH), 7.47-7.50 (2H, m, 2ArH), 7.64-7.67 (1H, m, ArH), 7.76 (1H, d, *J* = 8.5 Hz, ArH); ^{13}C-NMR δC (D_2O 100.61 MHz) 20.6 (CH_2), 21.4 (CH_2), 23.7 (CH_2), 29.2 (CH_2), 46.5 (CH_2), 46.9 (CH_2), 51.3 (CH_2), 57.3 (C-1), 60.6 (C-5), 69.7 (C-4), 75.0 (C-2), 78.8 (C-3), 114.3 (Ar), 116.3 (Ar), 120.9 (Ar), 123.5 (2Ar), 125.1 (Ar), 131.7 (Ar), 139.4 (Ar), 142.5 (Ar), 153.2 (Ar), 154.4 (Ar); HRMS (ESI): *m/z* $[M + H]^+$ calcd. for $C_{23}H_{31}N_6O_3$: 439.2452; found: 439.2453.

N-((1-(2-((1,2,3,4-Tetrahydroacridin-9-yl)amino)ethyl)-1*H*-1,2,3-triazol-4-yl)methyl)-1,4-dideoxy-1,4-imino-D-arabinitol (**9a**)

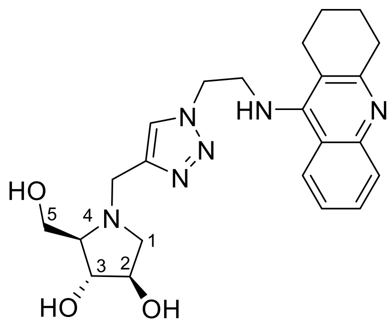

9a

The crude product was purified by silica gel flash column chromatography ($MeCN/H_2O/NH_4OH$, 190:10:1 → 180:20:1) (column 2) to obtain the title compound **9a** (16.4 mg, 63%) as a colorless oil. R_f 0.19 ($MeCN/H_2O/NH_4OH$, 15:5:0.2); $[\alpha]_D^{27}$ -20 (*c* 0.5, MeOH); The NMR data is in agreement with that of **9b**; HRMS (ESI): *m/z* $[M + H]^+$ calcd. for $C_{23}H_{31}N_6O_3$: 439.2452; found: 439.2450.

N-((1-(2-((1,2,3,4-Tetrahydroacridin-9-yl)amino)propyl)-1*H*-1,2,3-triazol-4-yl)methyl)-1,4-dideoxy-1,4-imino-L-arabinitol (**10b**)

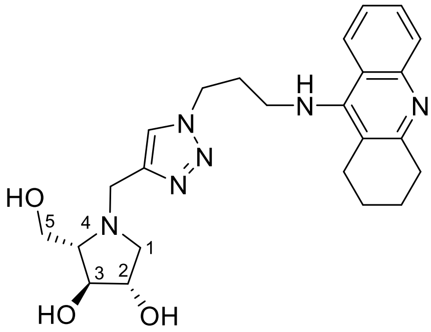

10b

The crude product was purified by silica gel flash column chromatography ($MeCN/H_2O/NH_4OH$, 190:10:1 → 180:20:1) (column 2) to obtain the title compound **10b** (28.3 mg, 90%) as a colorless oil. R_f 0.19 ($MeCN/H_2O/NH_4OH$, 15:5:0.2); $[\alpha]_D^{27}$ +5 (*c* 0.41, MeOH); ^{1}H-NMR δ_H (CD_3OD, 400.13 MHz) 1.95-1.97 (4H, m, $2CH_2$), 2.46-2.53 (2H, m, CH_2), 2.72 (2H, brs, CH_2), 3.04 (2H, brs, CH_2), ca. 3.31 (1H, H-1b, overlap with the NMR solvent signal), 3.37 (2H, brs, H-1a, H-4), 3.82-3.97 (3H, m, H-2, H-5a, H-5b), 3.99 (2H, t, *J* = 7.2 Hz, CH_2), 4.05 (1H, brs, H-3), 4.40 (1H, d, *J* = 14.2 Hz, CHbN), 4.54 (1H, d, *J* = 14.2 Hz, CHaN), 4.63 (2H, t, *J* = 6.6 Hz, CH_2), 7.55-7.59 (1H, m, ArH), 7.79-7.86 (2H, m, 2ArH), 8.30-8.32 (2H, m, 2ArH); ^{13}C-NMR δC (CD_3OD 100.61 MHz) 21.8 (CH_2), 23.0 (CH_2), 25.1 (CH_2), 29.4 (CH_2), 31.6 (CH_2), 46.1 (CH_2), *ca.* 49.0 (CH_2, overlap with the solvent signal), 50.1 (CH_2),

59.9 (C-1), 61.2 (C-5), 75.4 (C-4), 75.8 (C-3), 78.4 (C-2), 113.3 (Ar), 117.1 (Ar), 120.2 (Ar), 126.3 (Ar), 126.6 (Ar), 128.1 (Ar), 134.1 (Ar), 139.6 (2Ar), 152.1 (Ar), 158.1 (Ar); HRMS (ESI): *m/z* [M + H]$^+$ calcd. for $C_{24}H_{33}N_6O_3$: 453.2609; found: 453.2608.

N-((1-(2-((1,2,3,4-Tetrahydroacridin-9-yl)amino)propyl)-1*H*-1,2,3-triazol-4-yl)methyl)-1,4-dideoxy-1,4-imino-D-arabinitol (**10a**)

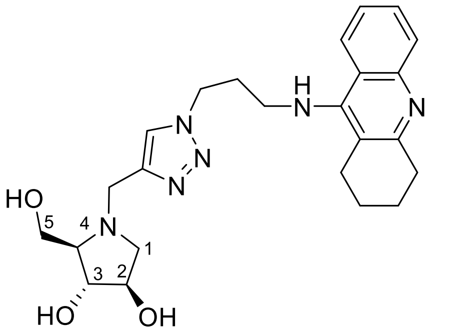

10a

The crude product was purified by silica gel flash column chromatography ($MeCN/H_2O/NH_4OH$, 190:10:1 → 180:20:1) (column 2) to obtain the title compound **10a** (24.7 mg, 87%) as a colorless oil. R_f 0.19 ($MeCN/H_2O/NH_4OH$, 15:5:0.2); $[\alpha]_D^{27}$ -3 (*c* 0.6, MeOH); The NMR data is in agreement for that of **10b**; HRMS (ESI): *m/z* [M + H]$^+$ calcd. for $C_{24}H_{33}N_6O_3$: 453.2609; found: 453.2607.

N-((1-(2-((1,2,3,4-Tetrahydroacridin-9-yl)amino)hexyl)-1*H*-1,2,3-triazol-4-yl)methyl)-1,4-dideoxy-1,4-imino-L-arabinitol (**11b**)

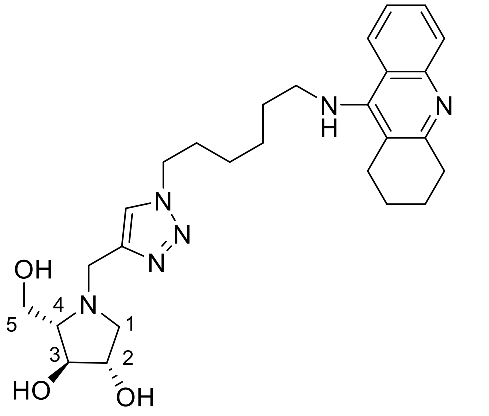

11b

The crude product was purified by silica gel flash column chromatography ($CH_2Cl_2/H_2O/NH_4OH$, 95:5:1 → 90:10:1) (column 2) to obtain the title compound **11b** (6.3 mg, 70%) as a colorless oil. R_f 0.2 ($CH_2Cl_2/MeOH/NH_4OH$, 15:5:0.2); $[\alpha]_D^{26.7}$ +14 (*c* 0.7, MeOH); ^{1}H-NMR δ_H (CD_3OD, 400.13 MHz) 1.26-1.32 (2H, m, CH_2), 1.35-1.41 (2H, m, CH_2), 1.59-1.67 (2H, m, CH_2), 1.84-1.92 (6H, m, $3CH_2$),

2.51 (1H, q, J = 4.0 Hz, H-4), 2.72-2.76 (3H, m, H-1a, CH_2), 2.82 (1H, d, J = 10.4 Hz, H-1b), 2.96-3.00 (2H, m, CH_2), 2.53 (2H, t, J = 7.2 Hz, CH_2), 3.63-3.76 (3H, m, H-5a, H-5b, CHaN), 3.85-3.87 (1H, m, H-2), 3.88-3.90 (1H, m, H-3), 4.05 (1H, d, J = 14.1 Hz, CHbN), 4.31-4.35 (2H, t, J = 7.0 Hz, CH_2), 7.35-7.39 (1H, m, ArH), 7.54-7.58 (1H, m, ArH), 7.75-7.78 (1H, m, ArH), 7.85 (1H, s, ArH), 8.08-8.11 (1H, m, ArH); ^{13}C-NMR δC (CD_3OD 100.61 MHz) 23.6 (CH_2), 24.1 (CH_2), 26.1 (CH_2), 27.1 (CH_2), 27.2 (CH_2), 31.1 (CH_2), 32.0 (CH_2), 34.0 (CH_2), 49.1 (CHaN, overlap solvent), 49.5 (NCH_2, overlap solvent), 51.1 (CH_2), 60.1 (C-1), 62.3 (C-5), 72.8 (C-4), 77.2 (C-2), 80.9 (C-3), 116.8 (Ar), 121.2 (Ar), 124.4 (Ar), 124.8 (Ar), 124.9 (Ar), 127.7 (Ar), 129.9 (Ar), 145.5 (Ar), 147.6 (Ar), 153.4 (Ar), 158.9 (Ar); HRMS (ESI): *m/z* $[M + H]^+$ calcd. for $C_{27}H_{39}N_6O_3$: 495.3078; found: 495.3078.

N-((1-(2-((1,2,3,4-Tetrahydroacridin-9-yl)amino)propyl)-1*H*-1,2,3-triazol-4-yl)methyl)-1,4-dideoxy-1,4-imino-D-arabinitol (**11a**)

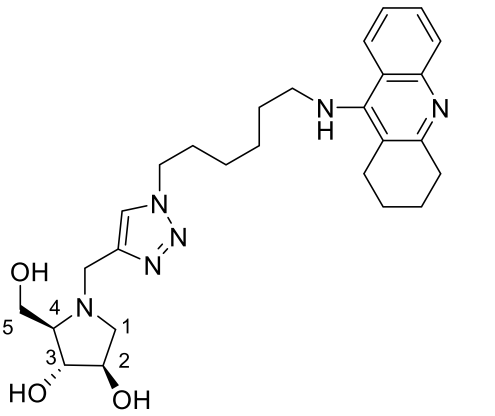

11a

The crude product was purified by silica gel flash column chromatography ($CH_2Cl_2/H_2O/NH_4OH$, 95:5:1 → 90:10:1) (column 2) to obtain the title compound **11a** (21.6 mg, 74%) as a colorless oil. R_f 0.17 ($MeCN/H_2O/NH_4OH$, 15:5:0.2); $[\alpha]_D^{27}$ -17 (*c* 0.8, MeOH); NMR data is in agreement for that of **11b**.; HRMS (ESI): *m/z* $[M + H]^+$ calcd. for $C_{27}H_{39}N_6O_3$: 495.3078; found: 495.3073.

Modelling studies

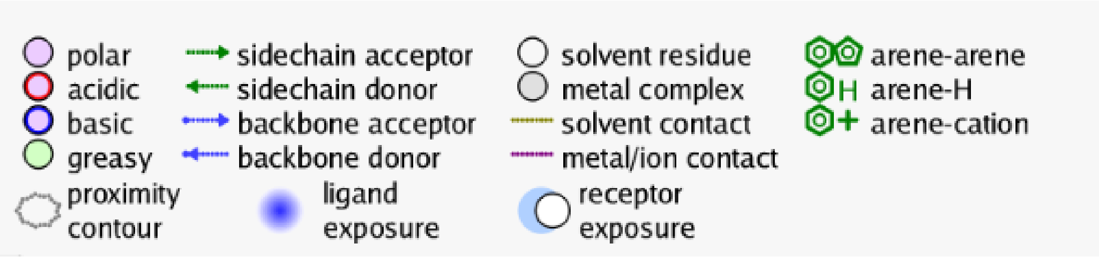

Figure SI1. Ligand-protein interaction types available in (or provided by) MOE.

Docking of **9a** and **9b** to *rh*AChE.

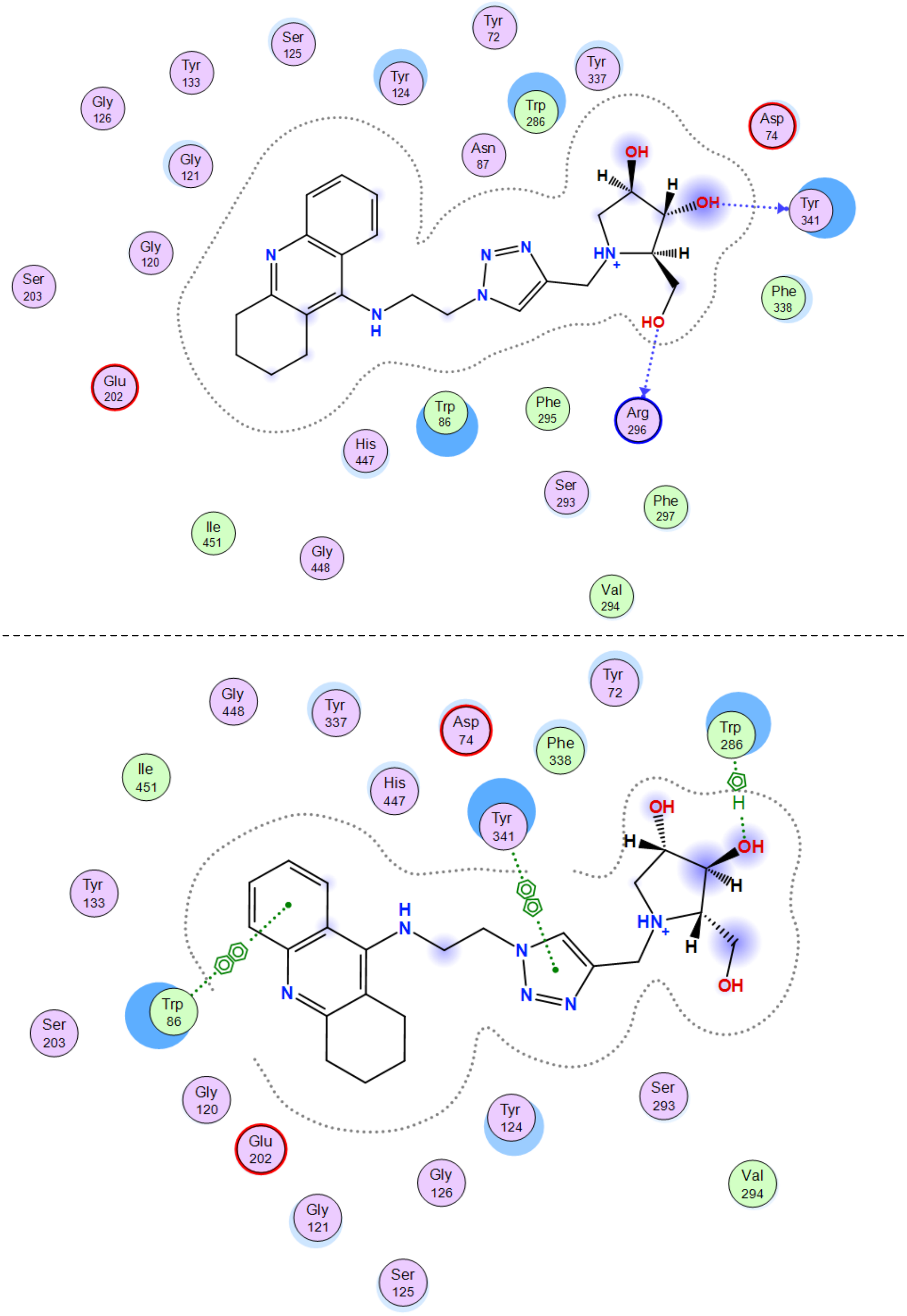

(**a**)

(**b**)

Figure SI2. Docking of (a) **9a** and (b) **9b** to *rh*AChE.

Docking of **10a** and **10b** to *rh*AChE

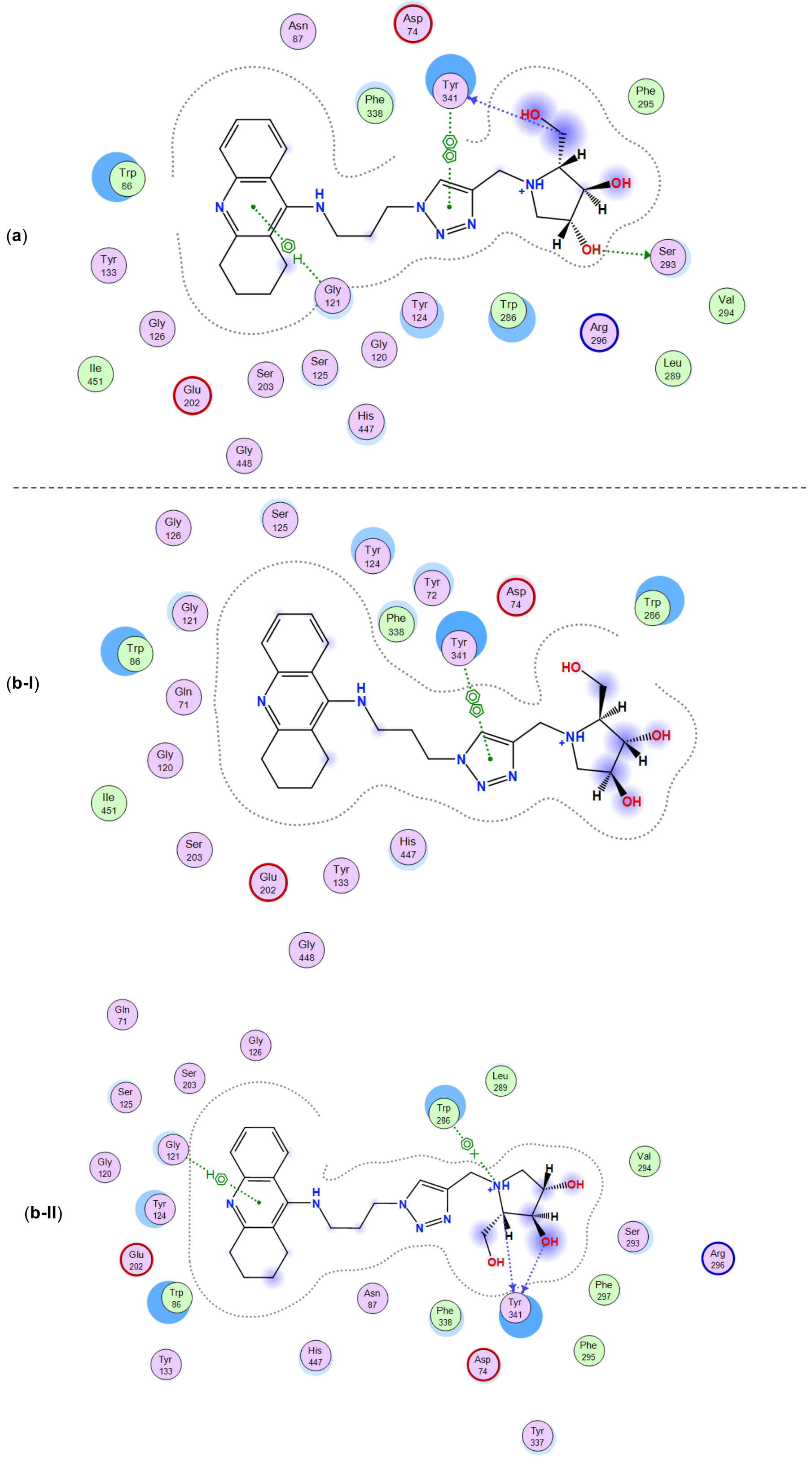

Figure SI3. Docking of (a) **10a** and (b) **10b** to *rh*AChE. The binding energies are identical for the poses of **10b** in b-I and b-II.

Docking of **11a** and **11b** to *rh*AChE.

(**a**)

(**b**)

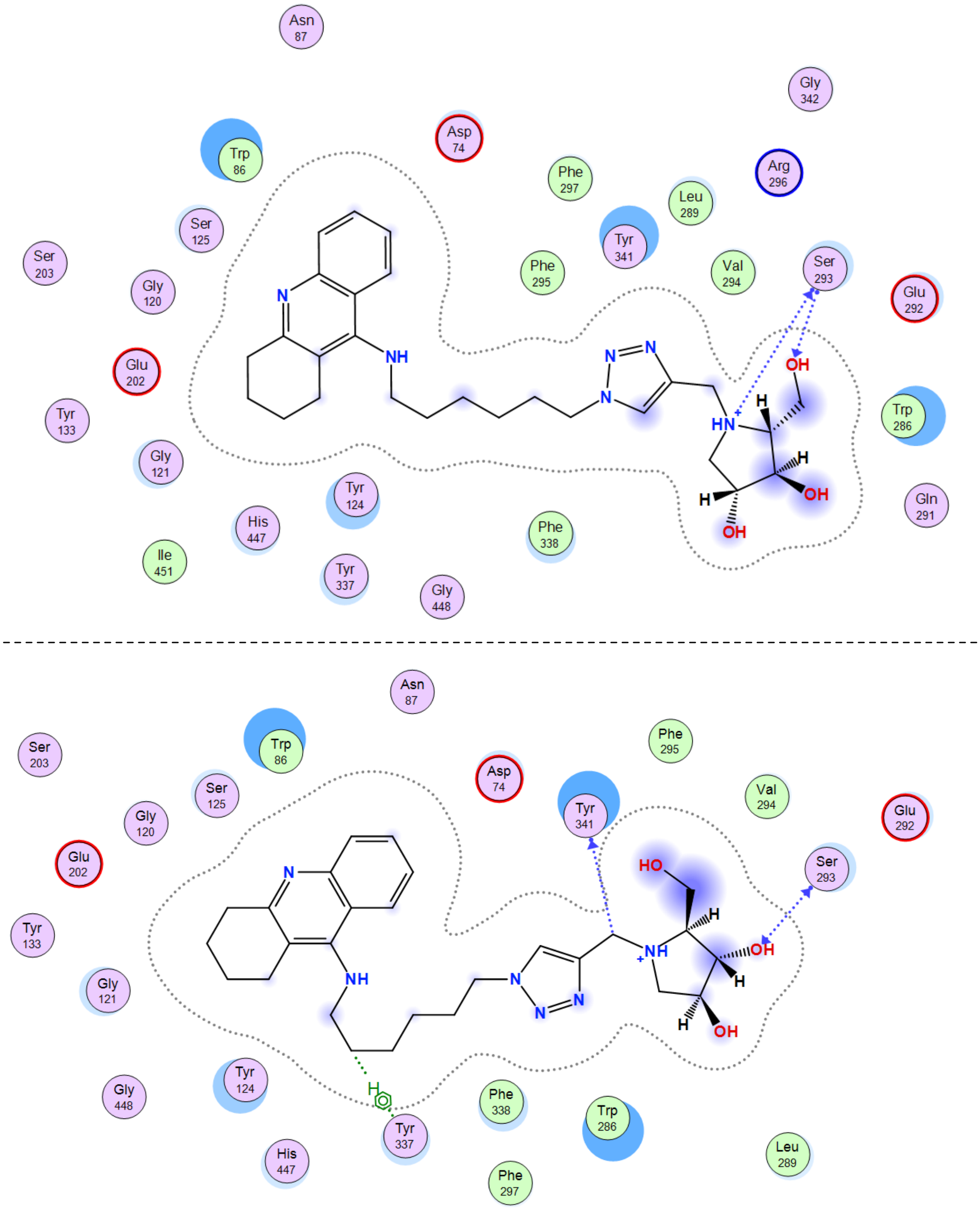

Figure SI4. Docking of (a) **11a** and (b) **11b** to *rh*AChE.

Docking of **9a** and **9b** to *h*BuChE.

(**a**)

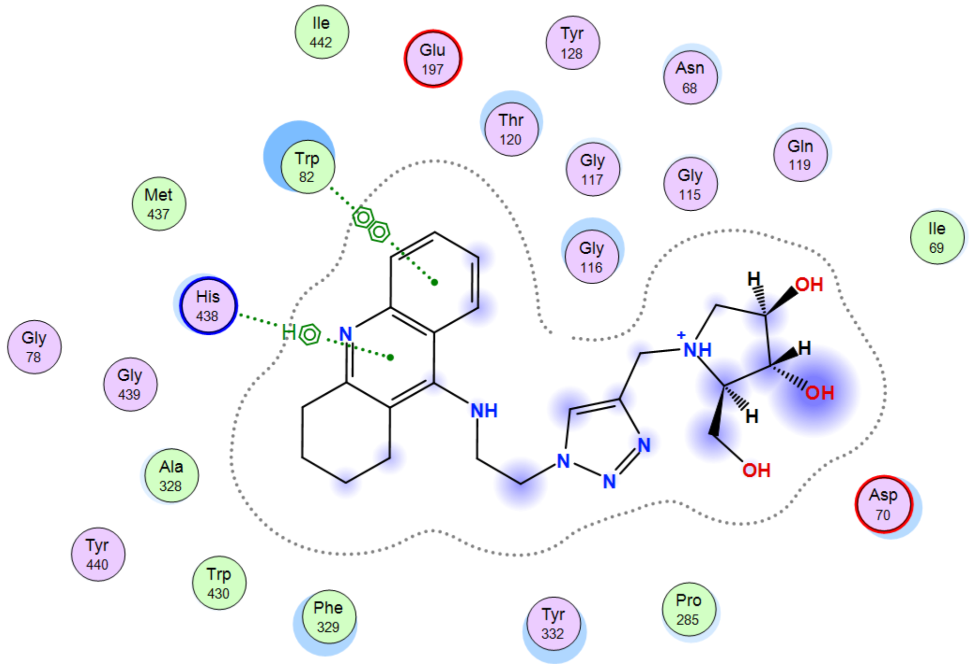

(**b**)

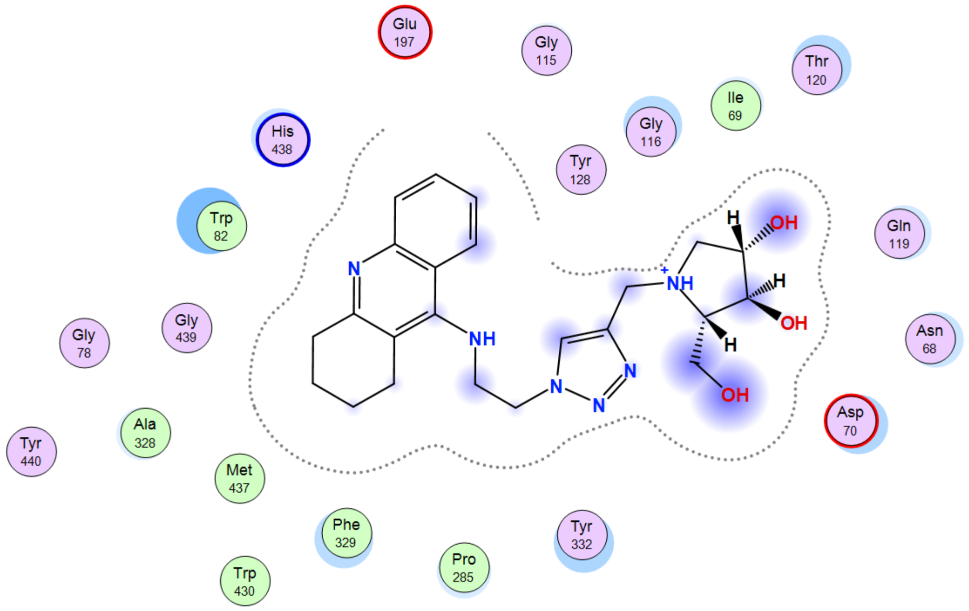

Figure SI5. Docking of (a) **9a** and (b) **9b** to *h*BuChE.

Docking of **10a** and **10b** to *h*BuChE.

(**a**)

(**b**)

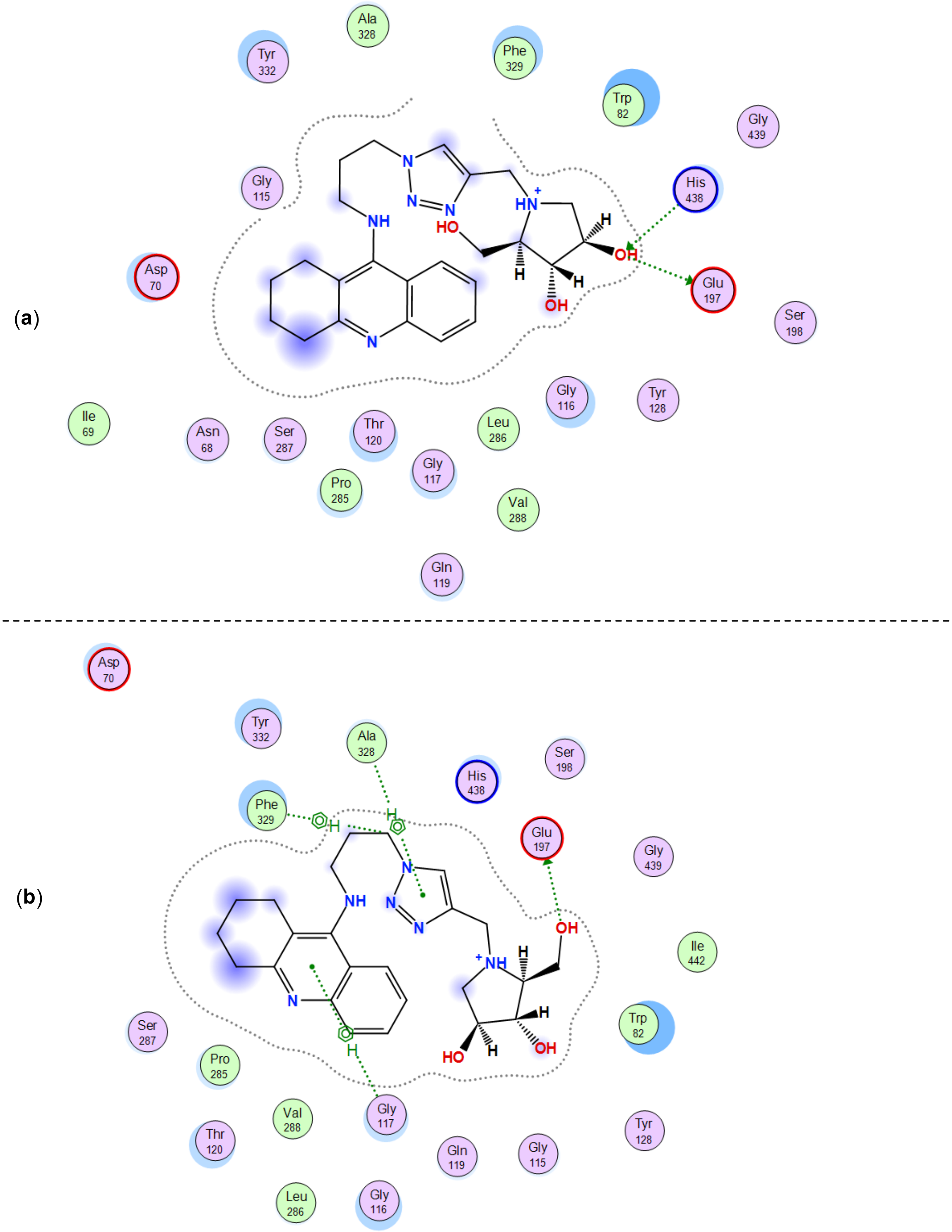

Figure SI6. Docking of (a) **10a** and (b) **10b** to *h*BuChE.

Docking of **11a** and **11b** to *h*BuChE

(**a**)

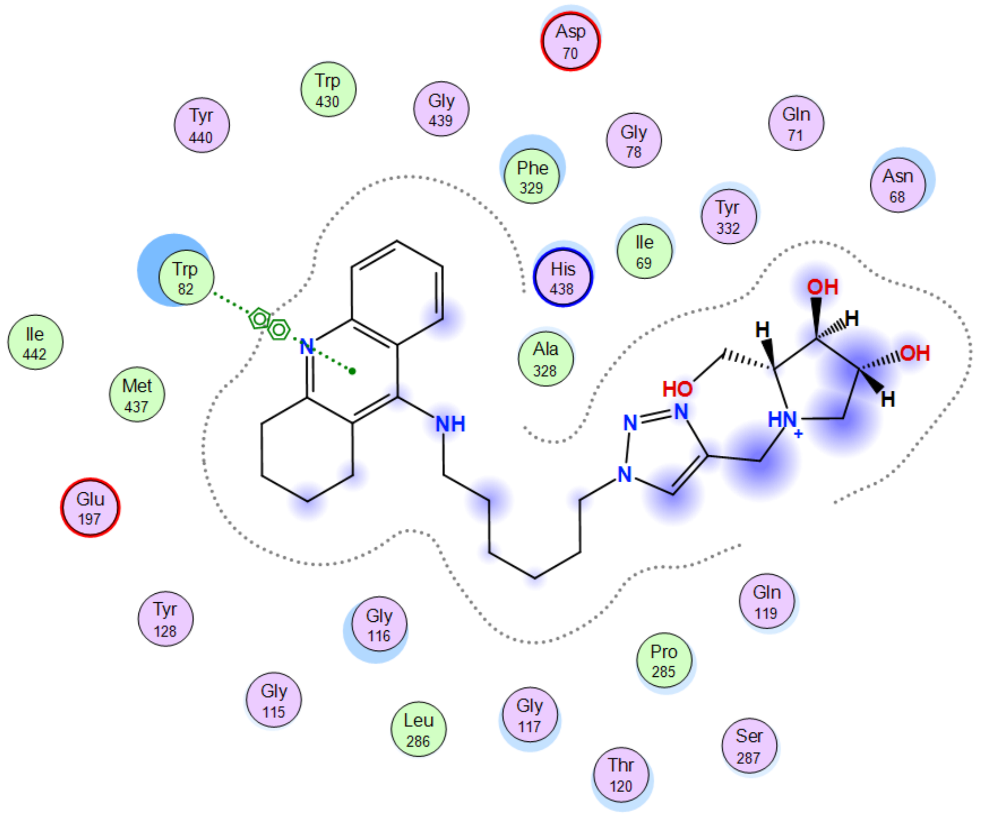

(**b**)

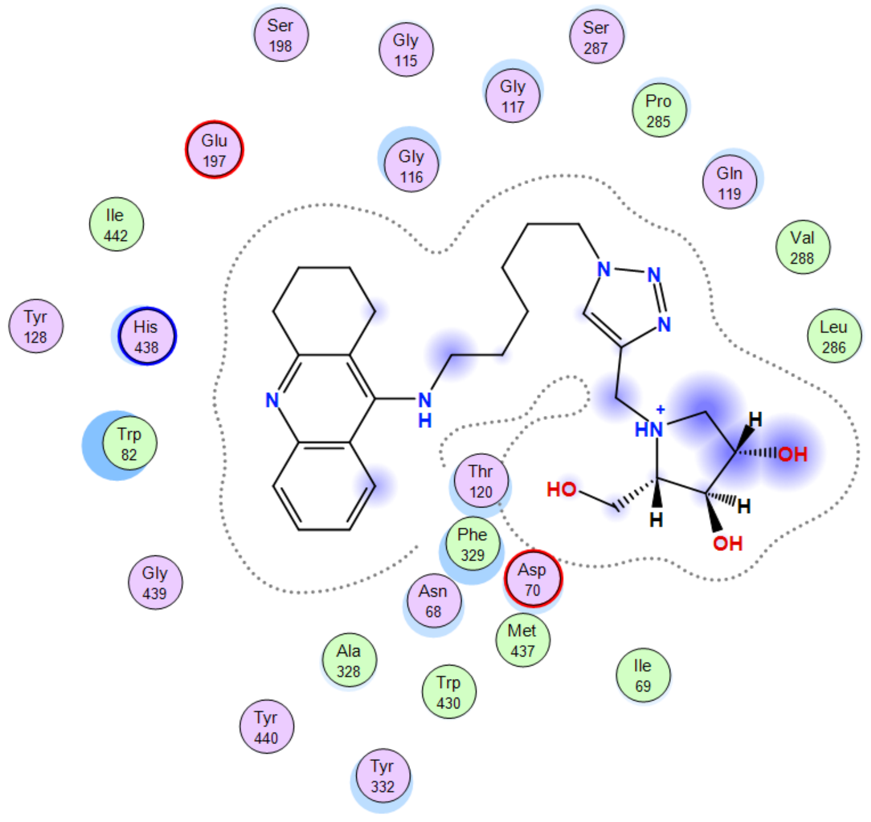

Figure SI7. Docking of (a) **11a** and (b) **11b** to *h*BuChE.

References

1. Santos Evangelista, T. C.; Ó. Lopéz; Sydnes, M. O.; Fernández-Bolaños, J. G.; Baptista Ferreira, S.; Lindbäck, E. Bicyclic 1-Azafagomine Derivatives: Synthesis and Glycosidase Inhibitory Testing. *Synthesis*, **2019**, *51*, 4066.
2. Rössler, S. L.; Schreib, B. S.; Ginterseder, M.; Hamilton, J. Y.; Carreira, E. M. Total Synthesis and Stereochemical Assignment of (+)-Broussonetine H. *Org. Lett*. **2017**, *19*, 5533.
3. Carmona, A. T.; Whigtman, R. H.; Robina, I.; Vogel, P. Synthesis and Glycosidase Inhibitory Activity of 7-Deoxycasuarine. *Helv. Chim. Acta*. **2003**, *86*, 3066.
4. D'Adamio, G. et al. Evidence for a multivalent effect in inhibition of sulfatases involved in lysosomal storage disorders (LSDs). *RSC advances*. **2016**, *6*, 64847.
5. Jaszczyk, J.; Li, S.; Cocaud, C.; Nicolas, C.; Martin, O. R. A practical approach to Dideoxy-1, 4-and 1, 5-iminopentitols from protected sugar hemiacetals. *Carbohydr. Res.* **2019**, *486*, 107855.

NMR Spectrum

^{1}H-NMR spectrum of compound **15b** ($CDCl_3$, 400.13 MHz)

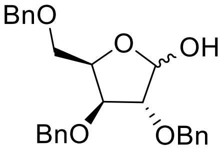

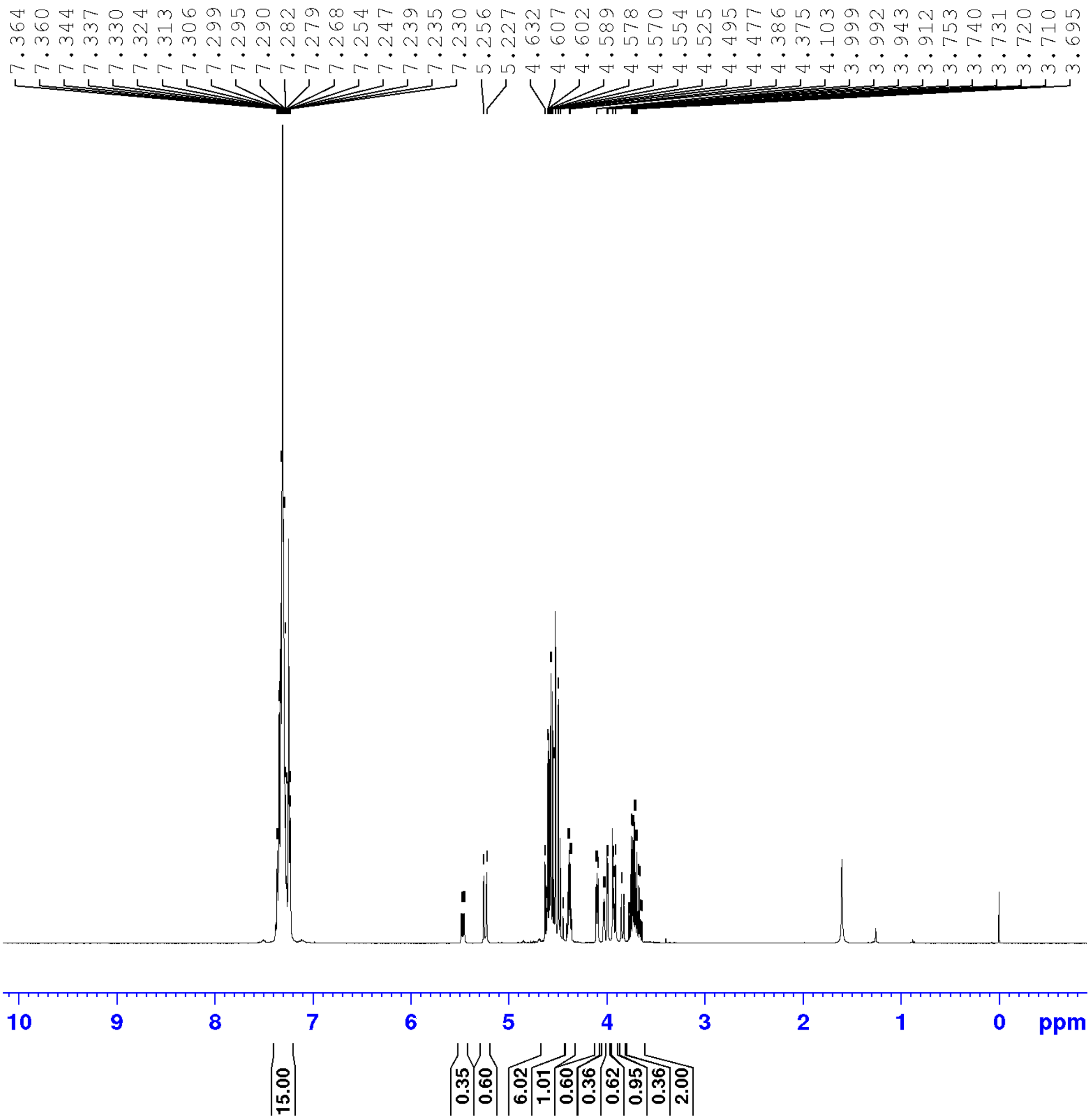

^{13}C-NMR spectrum of compound **15b** ($CDCl_3$, 100.61 MHz)

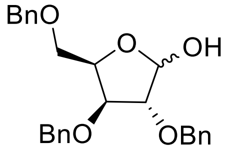

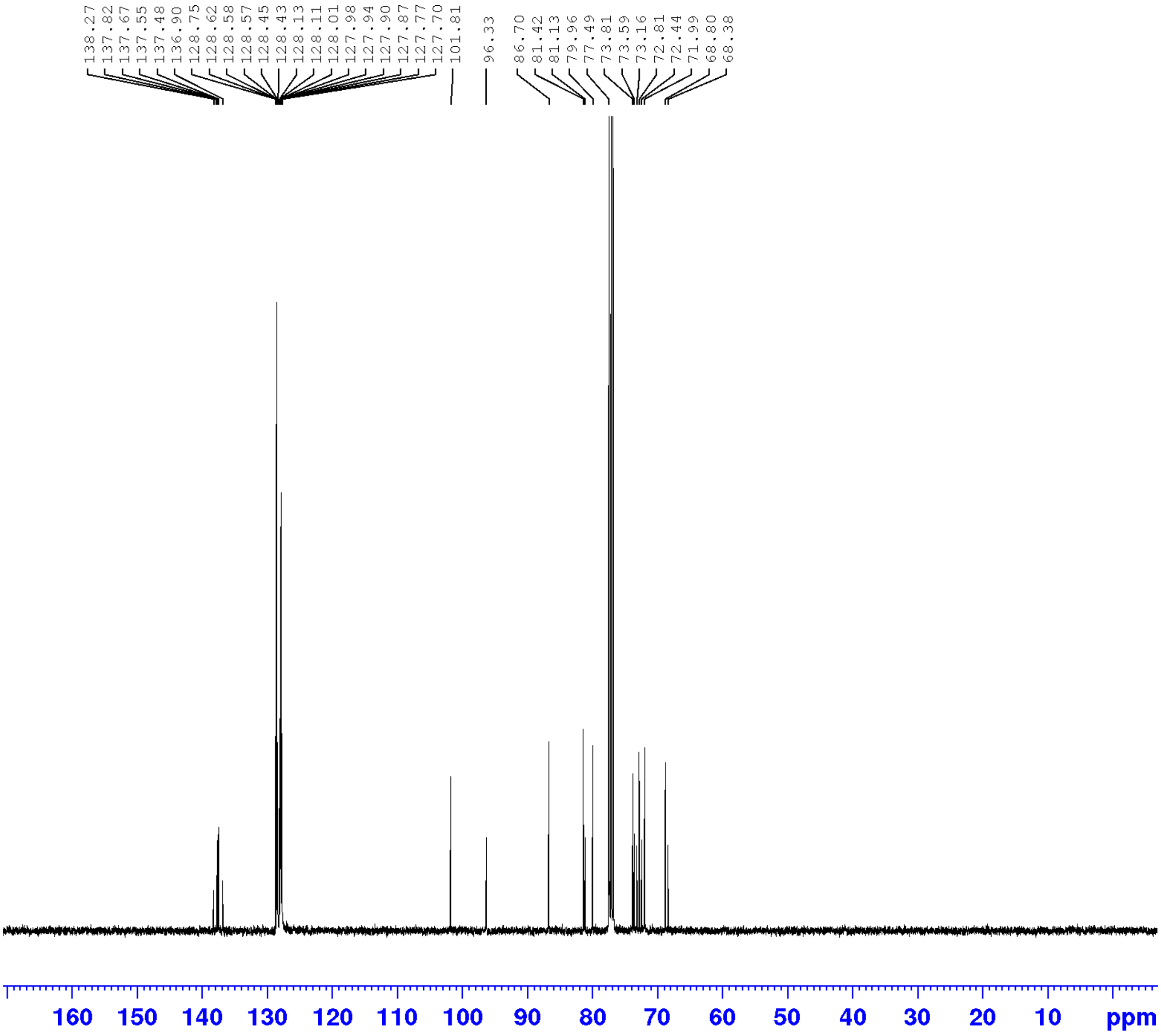

^{1}H-NMR spectrum of compound **15a** ($CDCl_3$, 400.13 MHz)

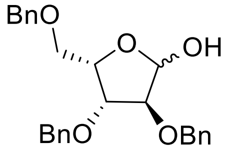

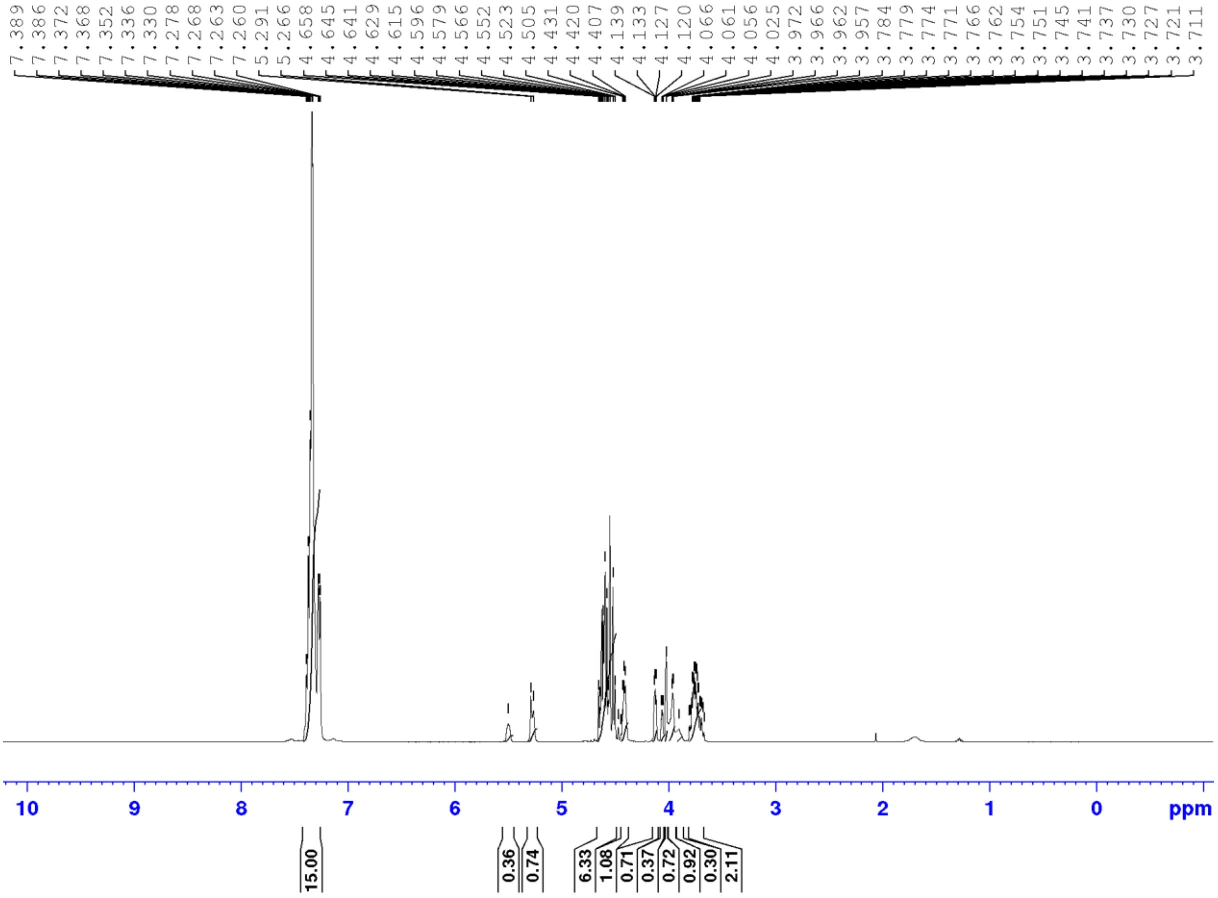

^{13}C-NMR spectrum of compound **15a** ($CDCl_3$, 100.61 MHz

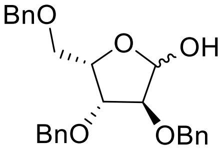

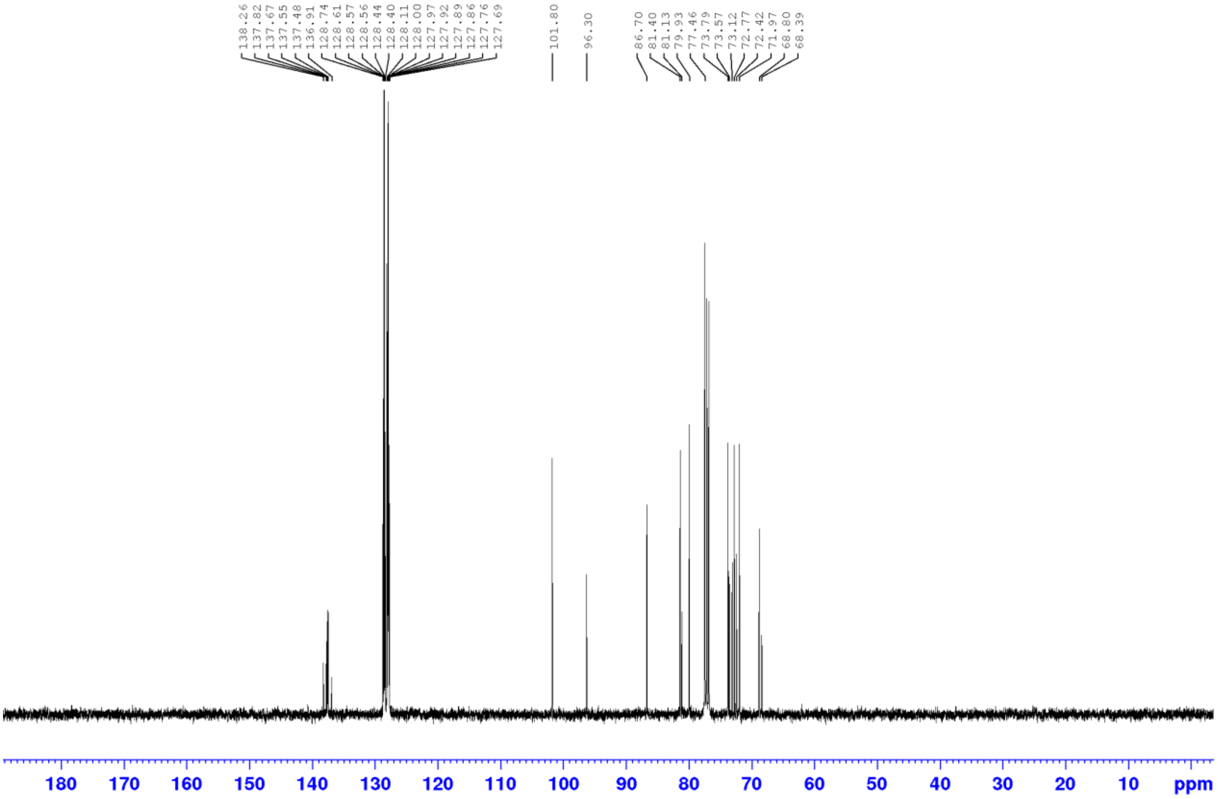

^{1}H-NMR spectrum of compound **16b** (CD_3OD, 400.13 MHz)

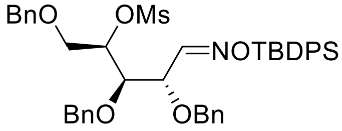

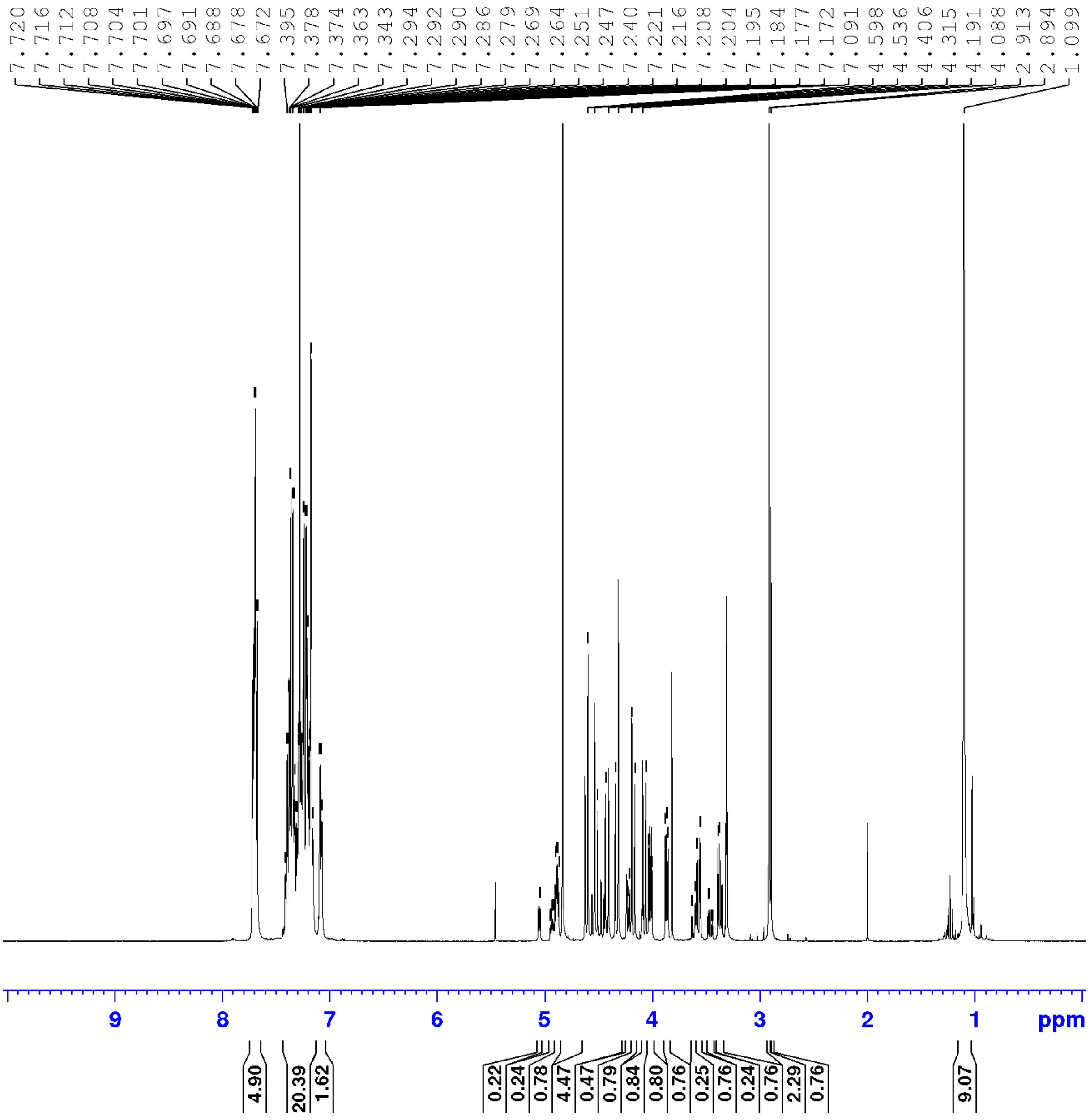

^{1}H-NMR spectrum of compound **16a** (CD_3OD, 400.13 MHz)

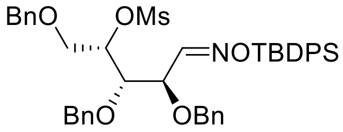

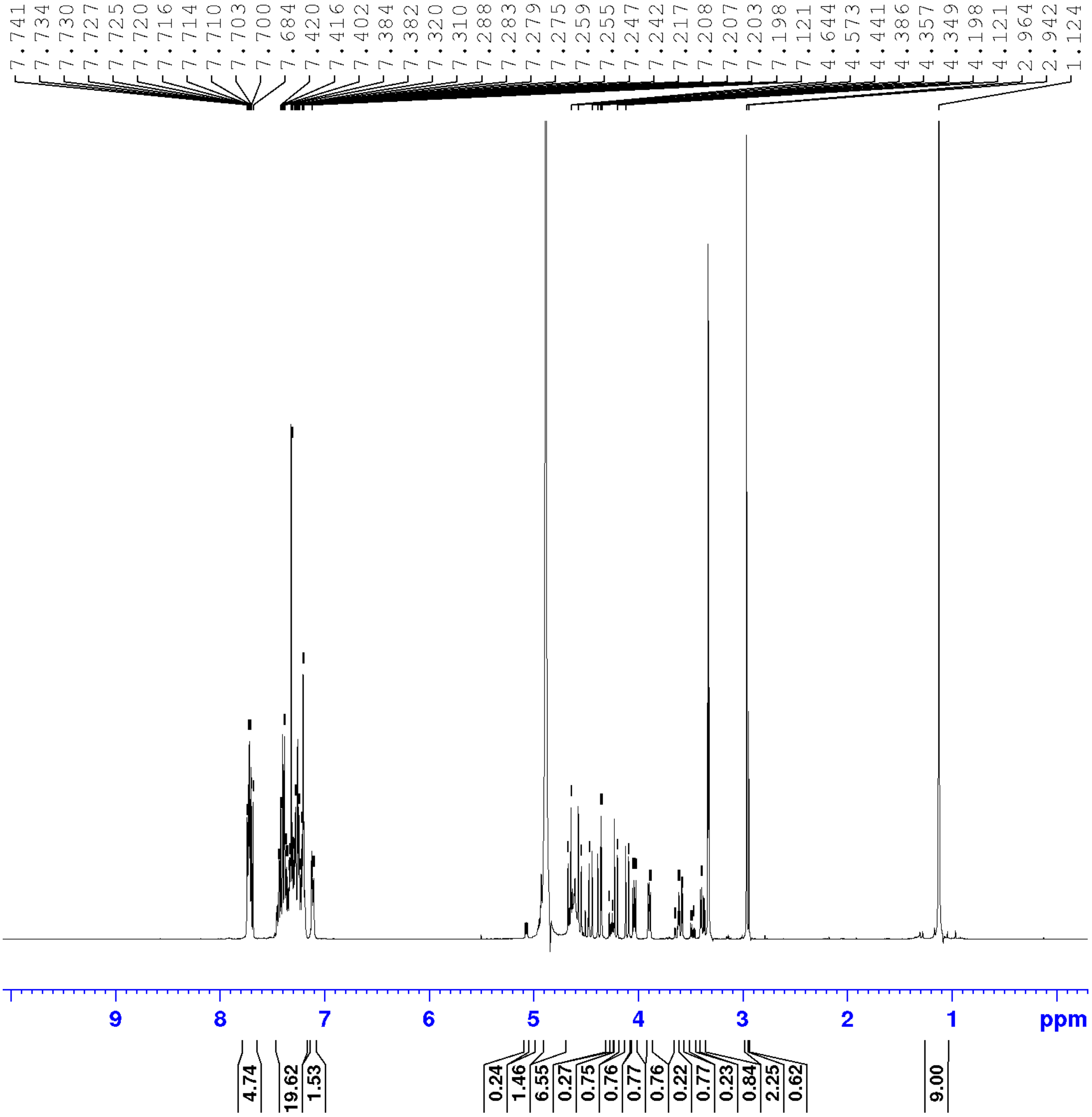

^{1}H-NMR spectrum of compound **17b** ($CDCl_3$, 400.13 MHz)

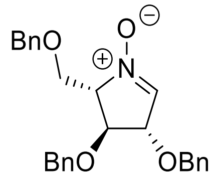

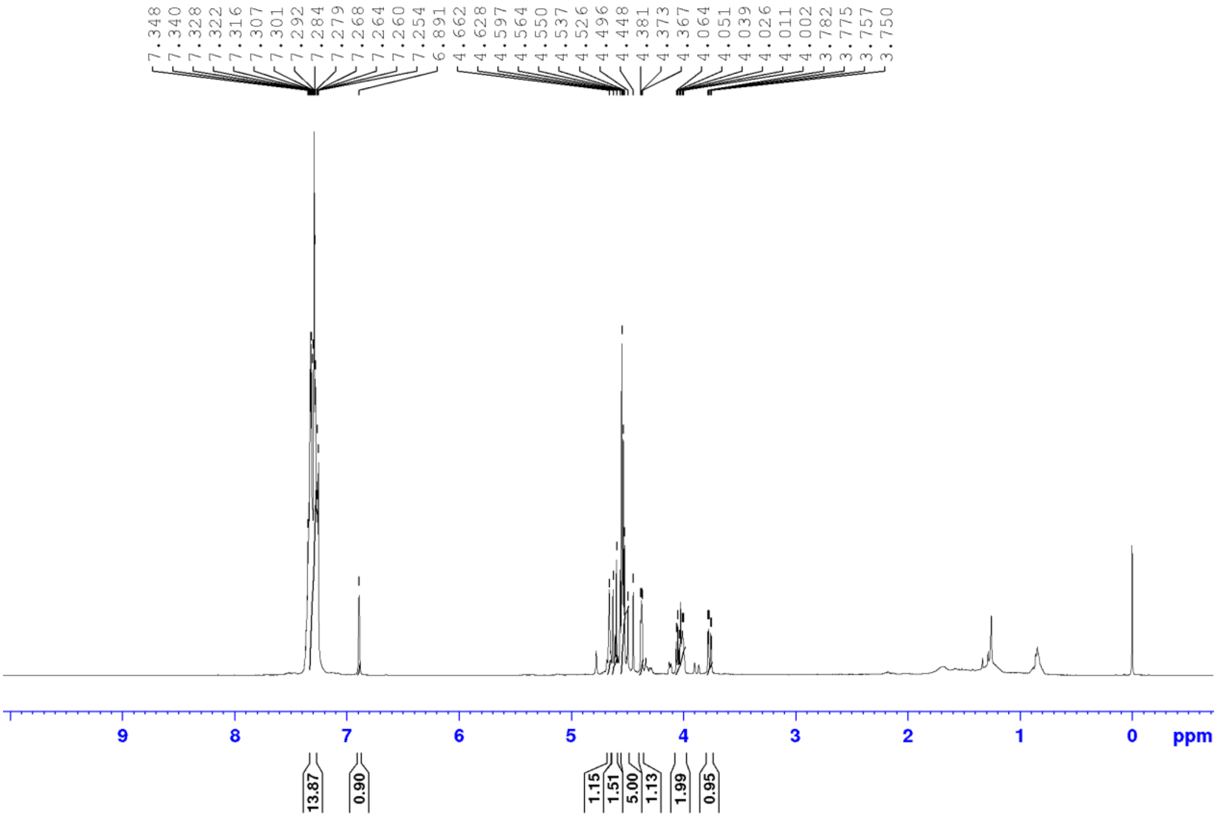

^{13}C-NMR spectrum of compound **17b** ($CDCl_3$, 100.61 MHz)

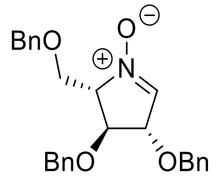

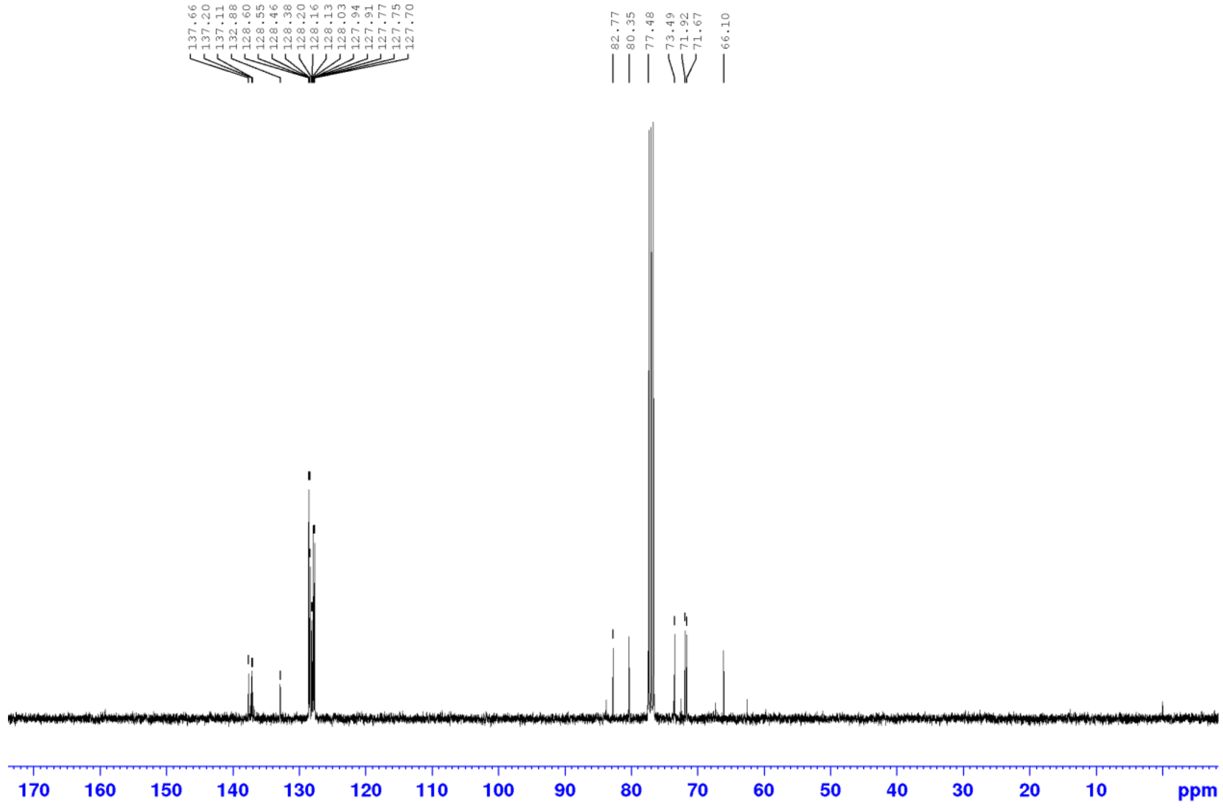

^{1}H-NMR spectrum of compound **17a** ($CDCl_3$, 400.13 MHz)

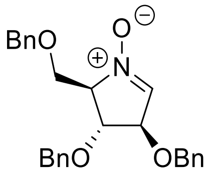

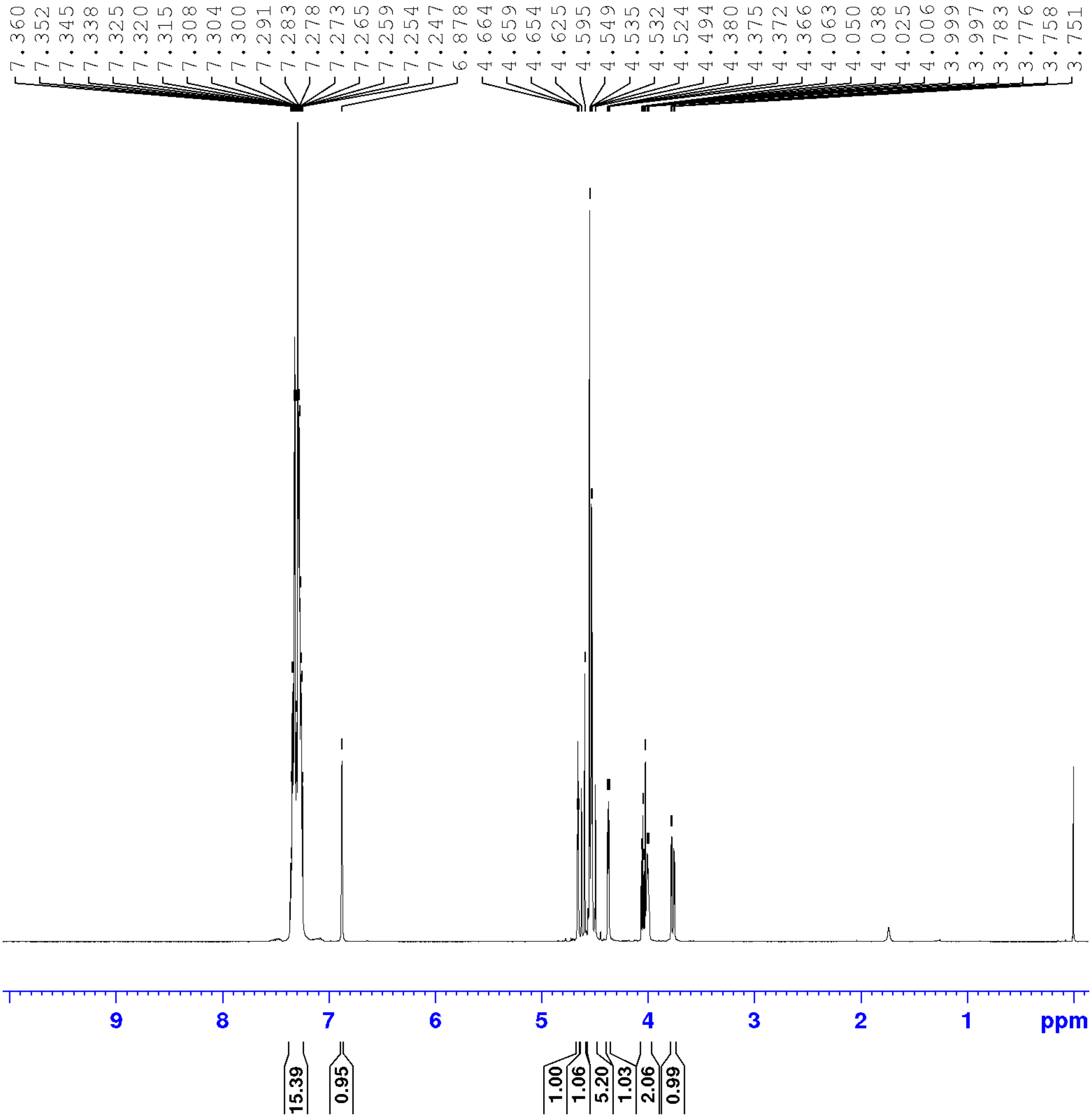

^{13}C-NMR spectrum of compound **17a** ($CDCl_3$, 100.61 MHz)

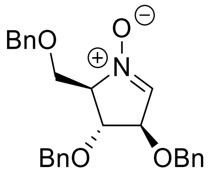

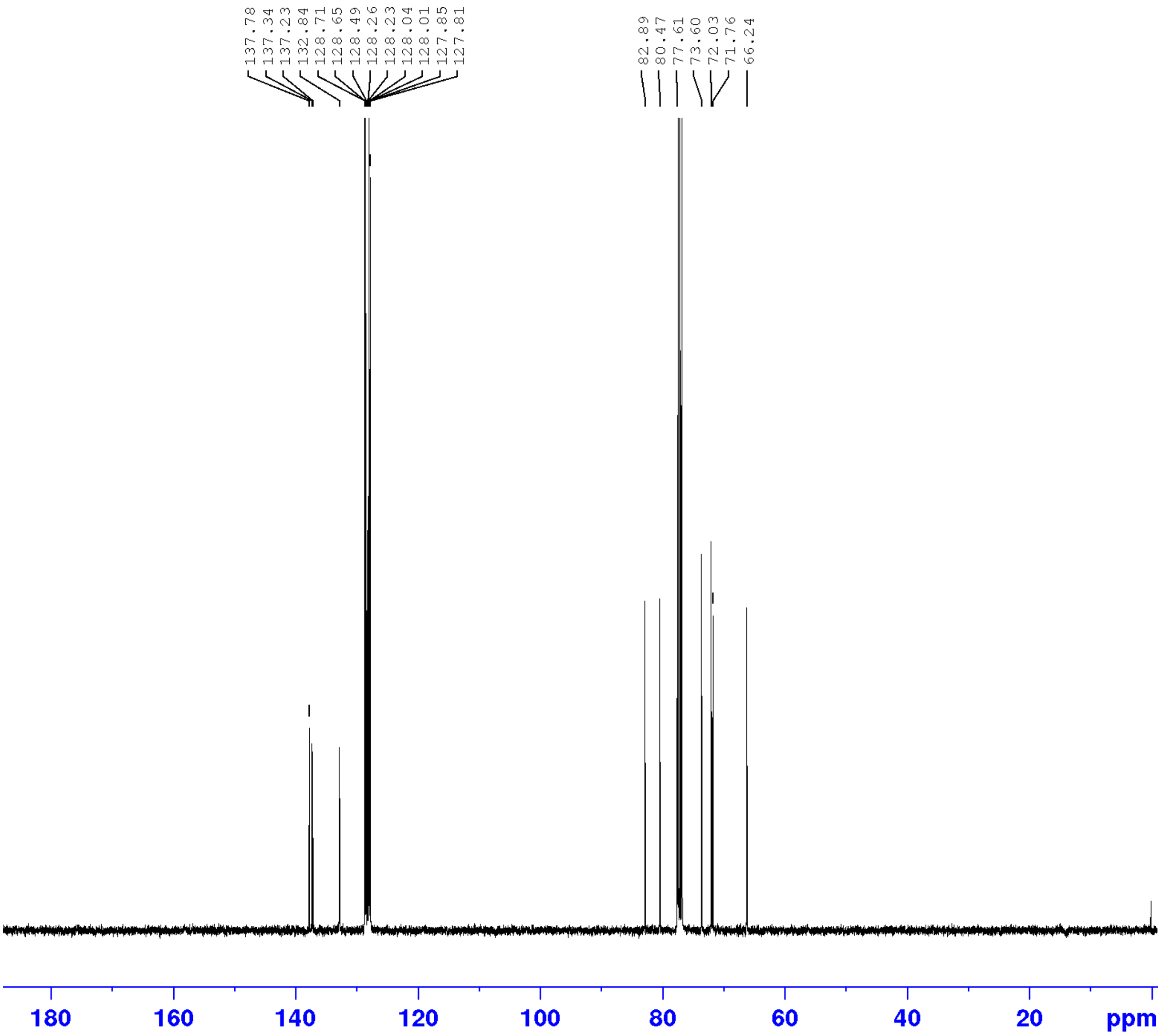

^{1}H-NMR spectrum of compound **18b** ($CDCl_3$, 400.13 MHz)

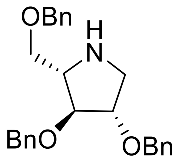

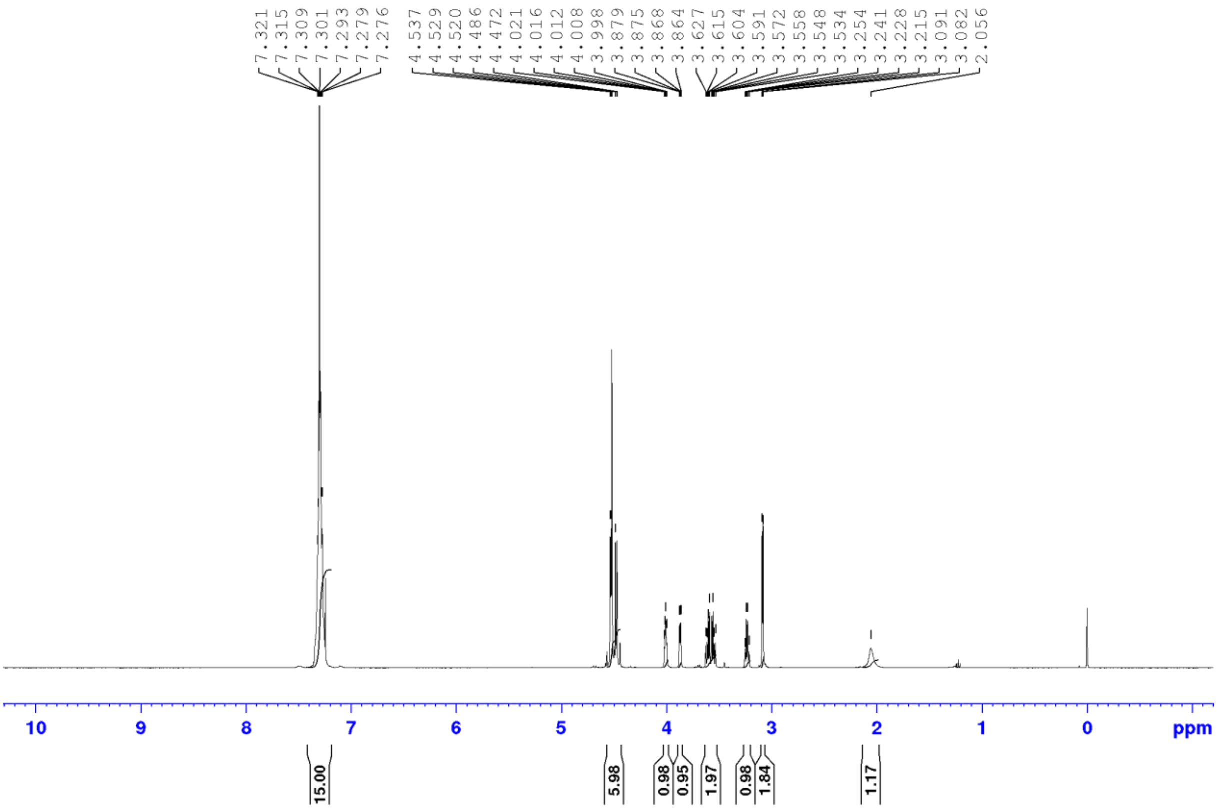

^{13}C-NMR spectrum of compound **18b** ($CDCl_3$, 100.61 MHz)

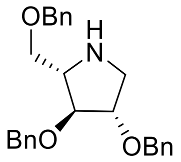

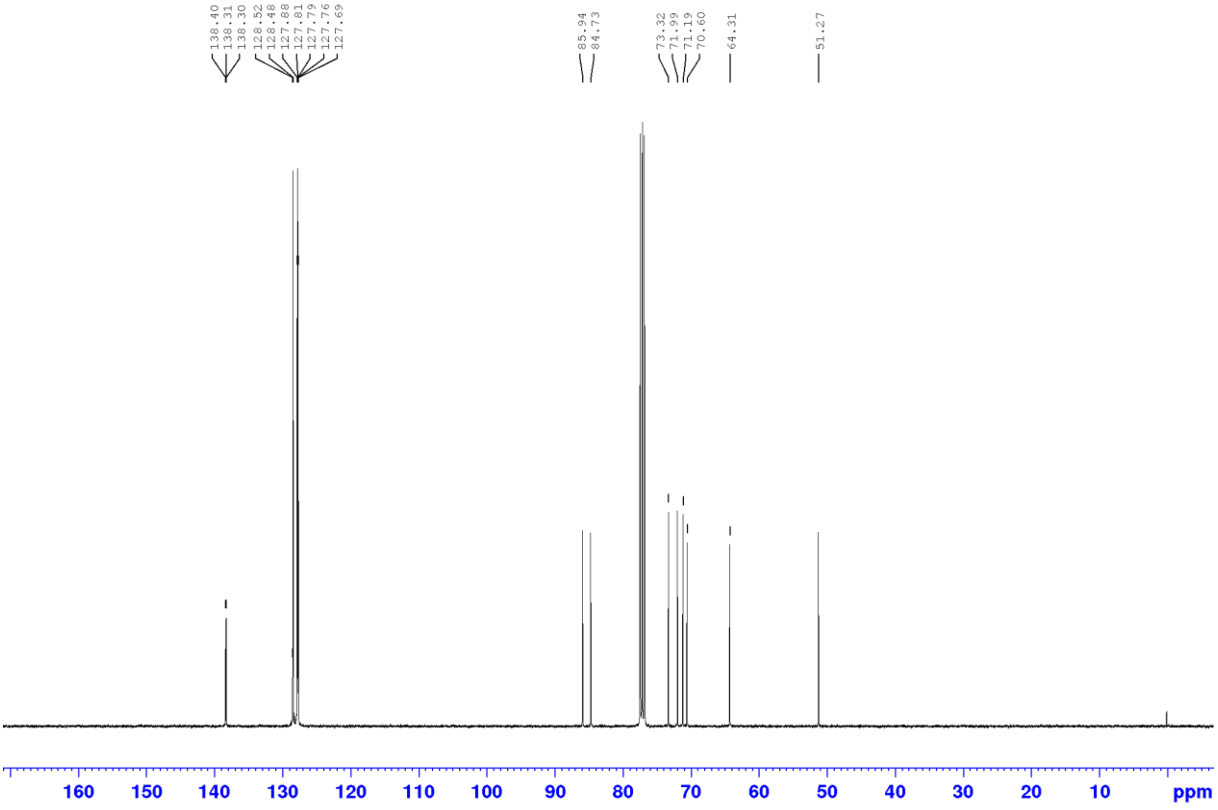

^{1}H-NMR spectrum of compound **18a** ($CDCl_3$, 400.13 MHz)

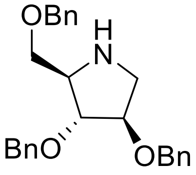

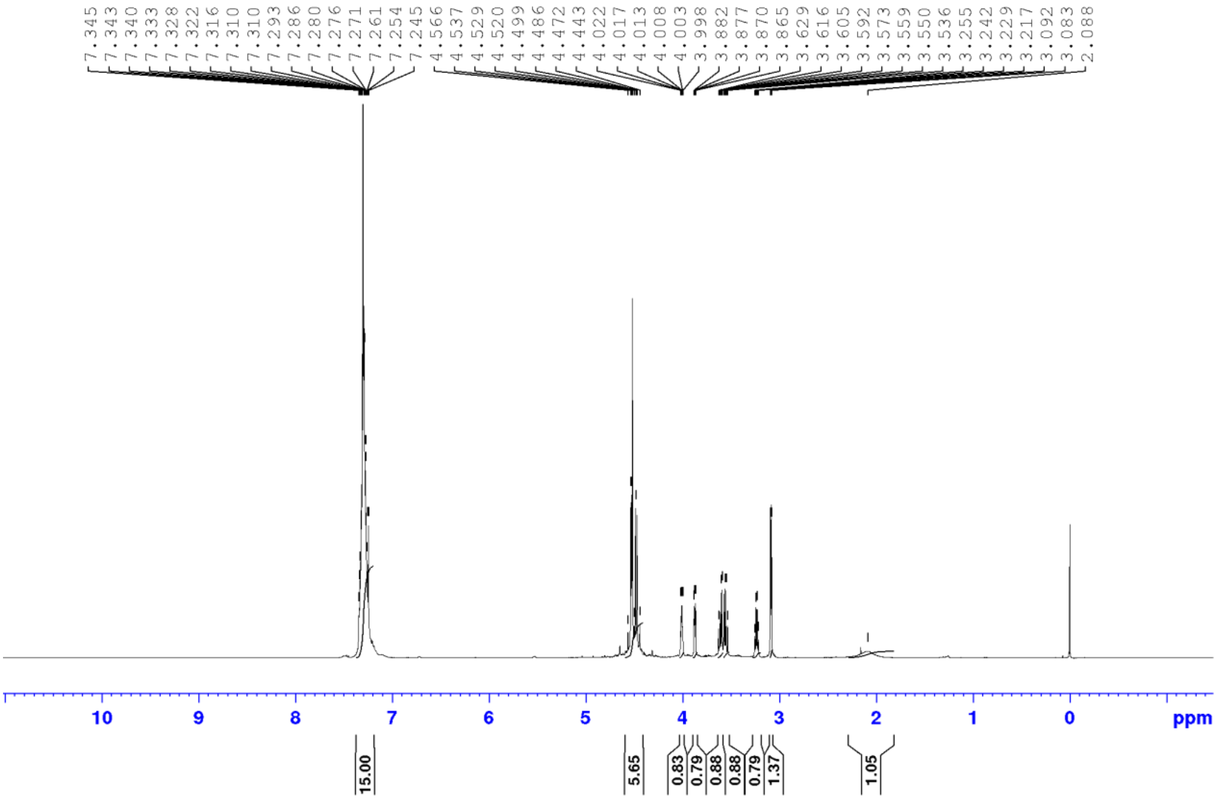

^{13}C-NMR spectrum of compound **18a** ($CDCl_3$, 100.61 MHz)

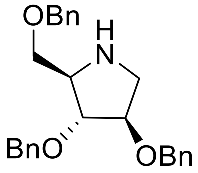

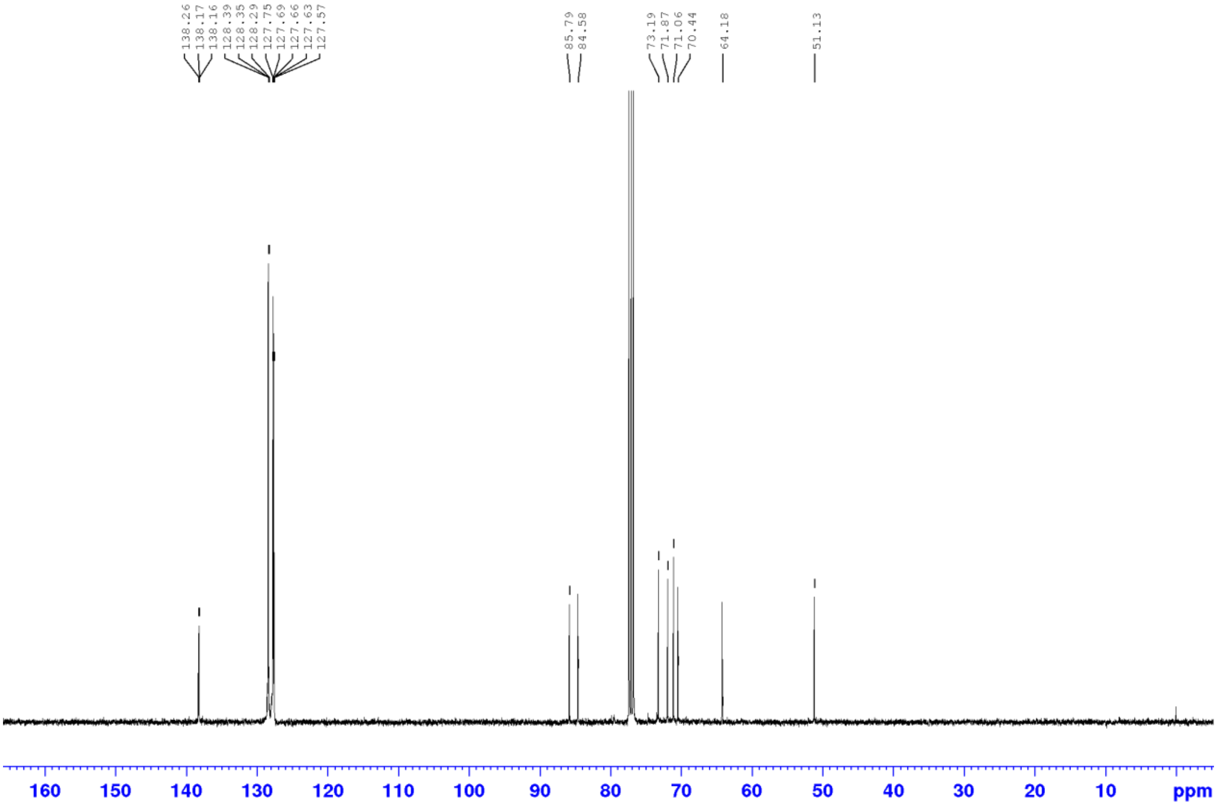

^{1}H-NMR spectrum of compound **19a** ($CDCl_3$, 400.13 MHz)

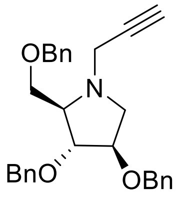

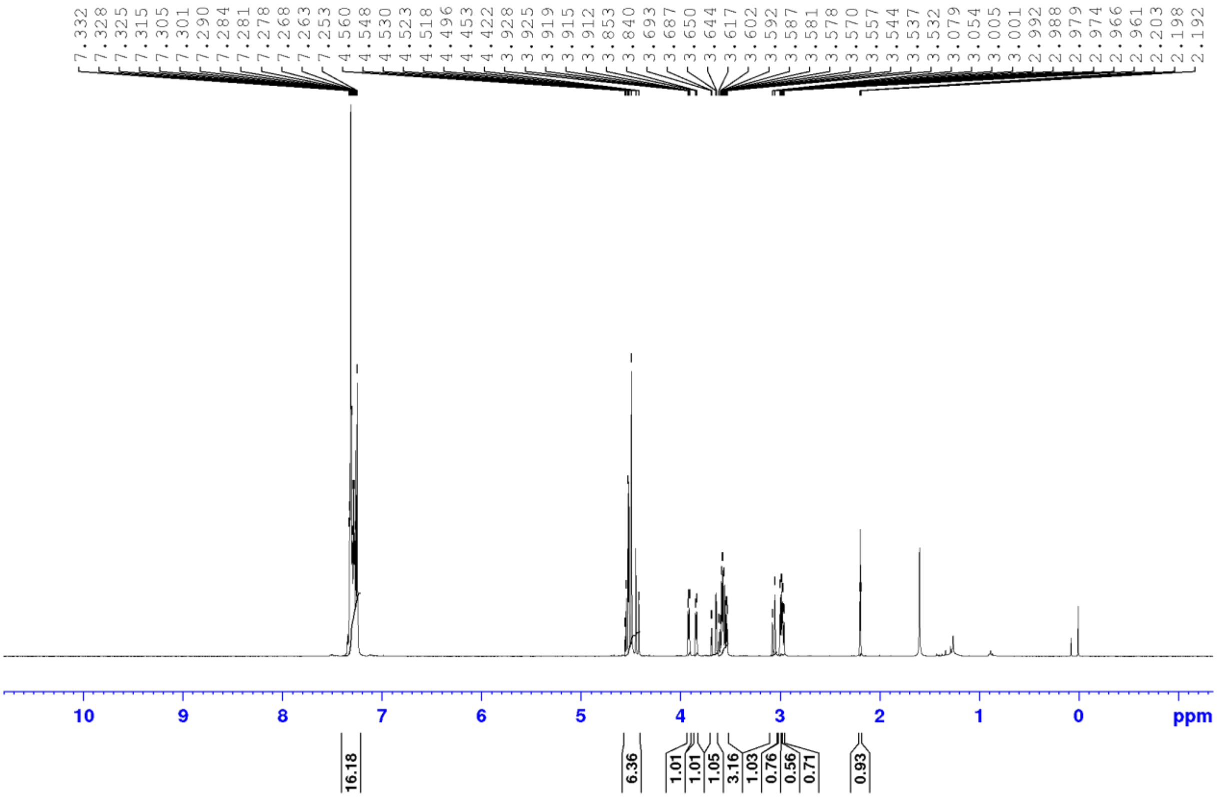

^{13}C-NMR spectrum of compound **19a** ($CDCl_3$, 100.61 MHz)

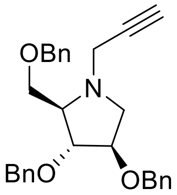

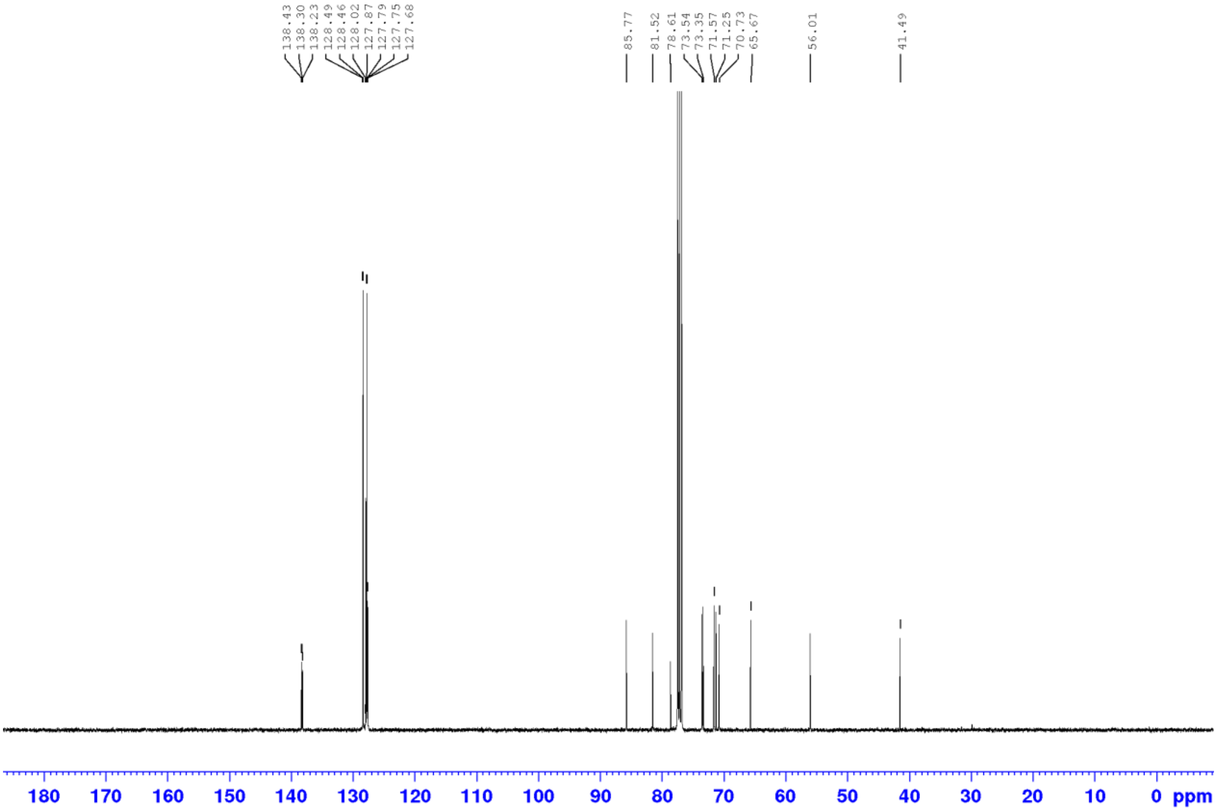

^{1}H-NMR spectrum of compound **19b** ($CDCl_3$, 400.13 MHz)

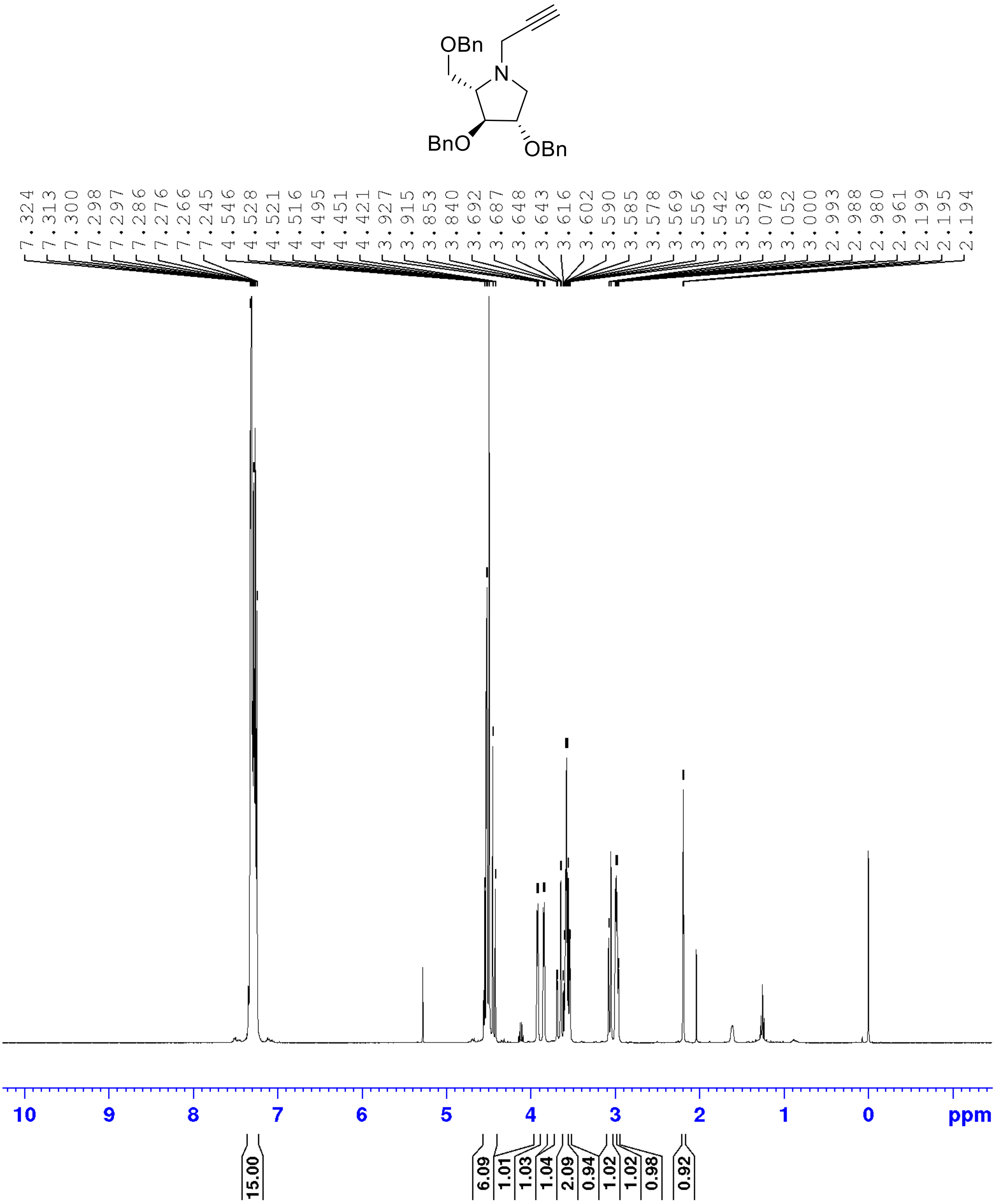

^{13}C-NMR spectrum of compound **19b** ($CDCl_3$, 100.61 MHz)

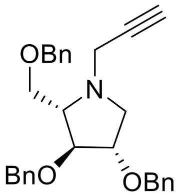

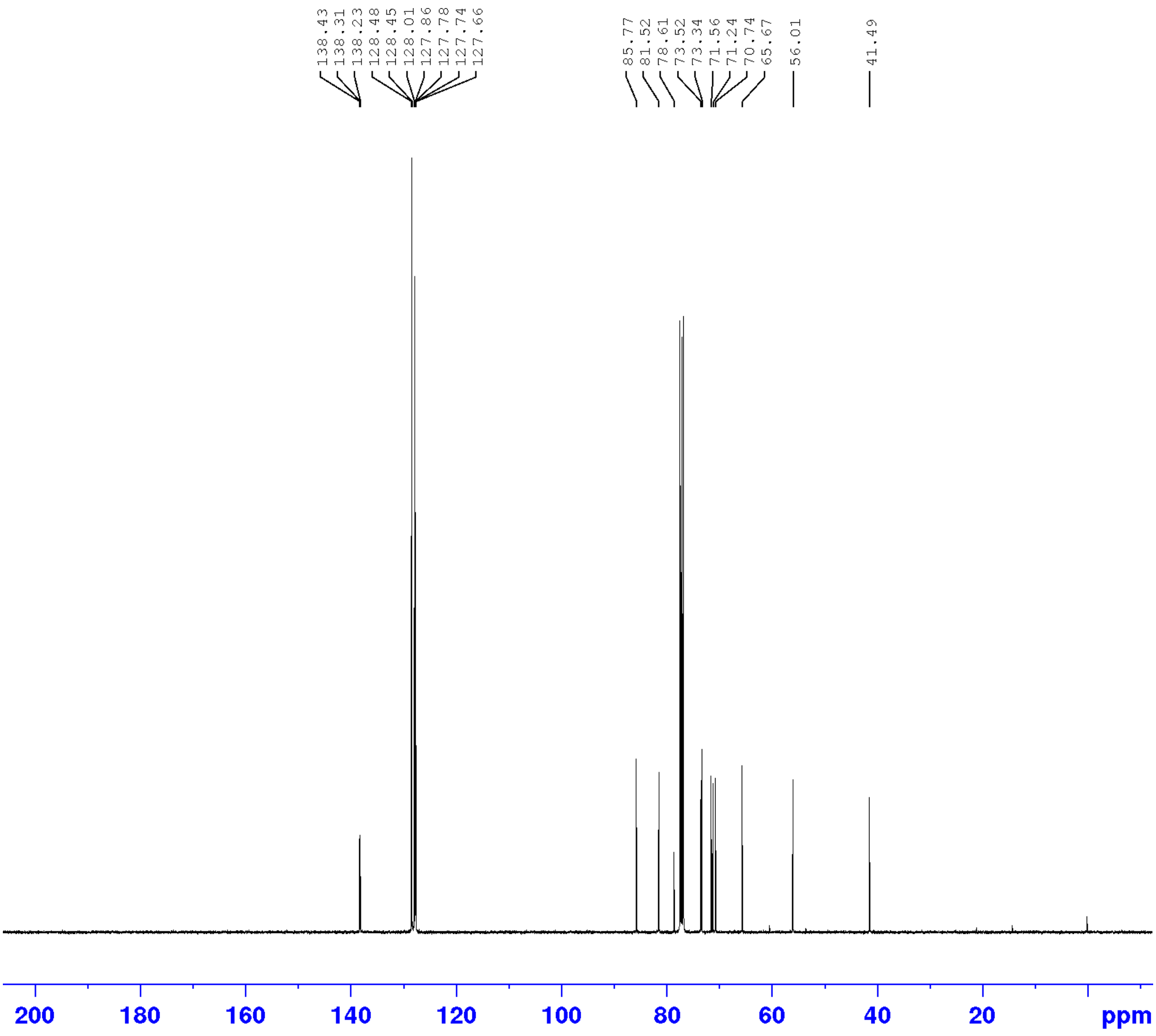

^{1}H-NMR spectrum of compound **20b** ($CDCl_3$, 400.13 MHz)

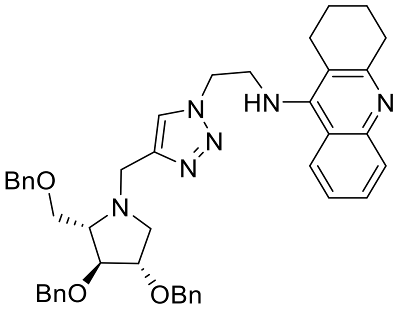

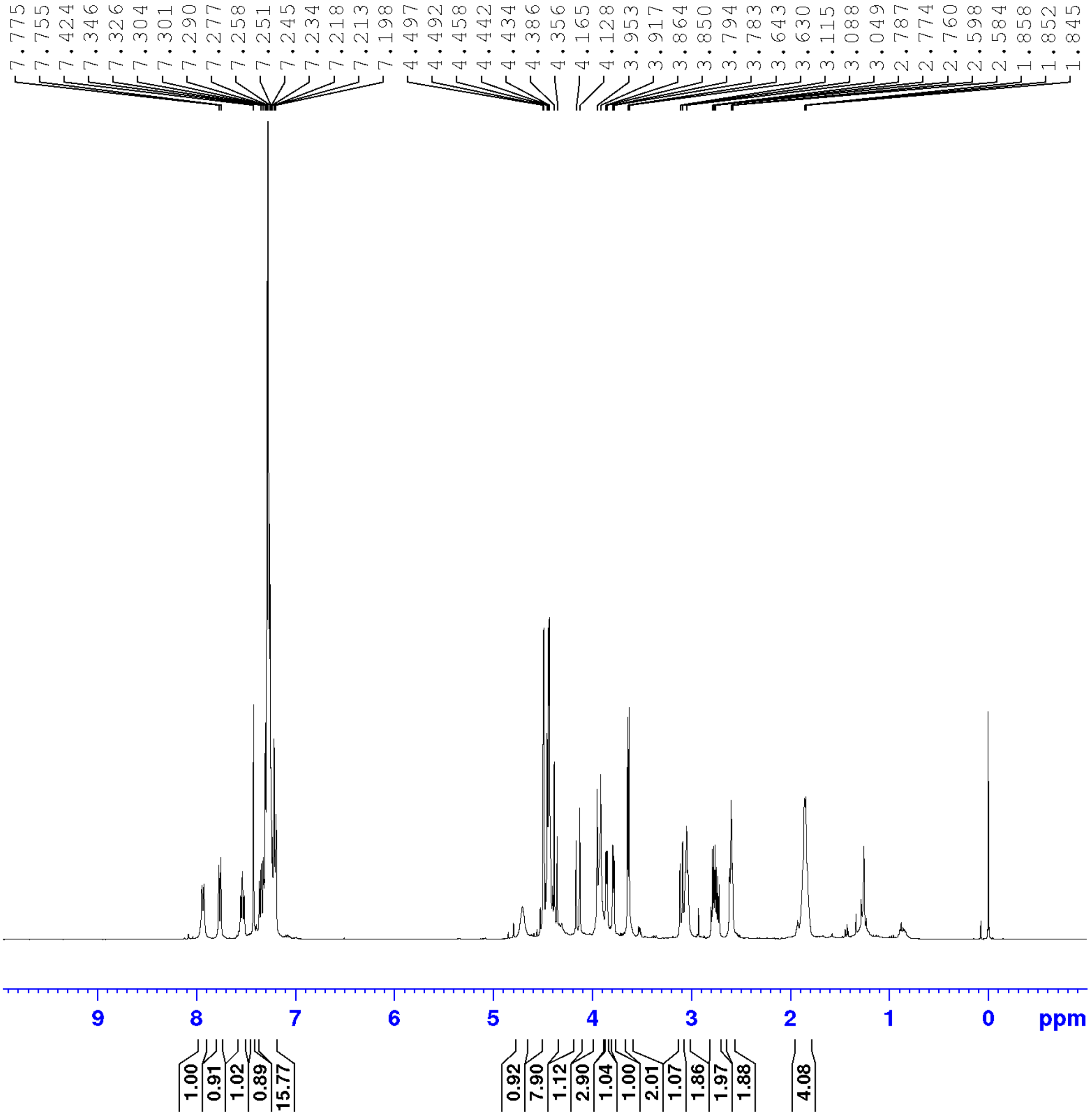

^{13}C-NMR spectrum of compound **20b** ($CDCl_3$, 100.61 MHz)

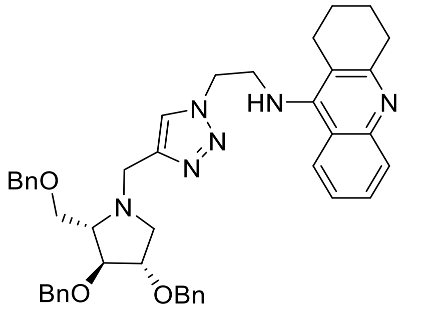

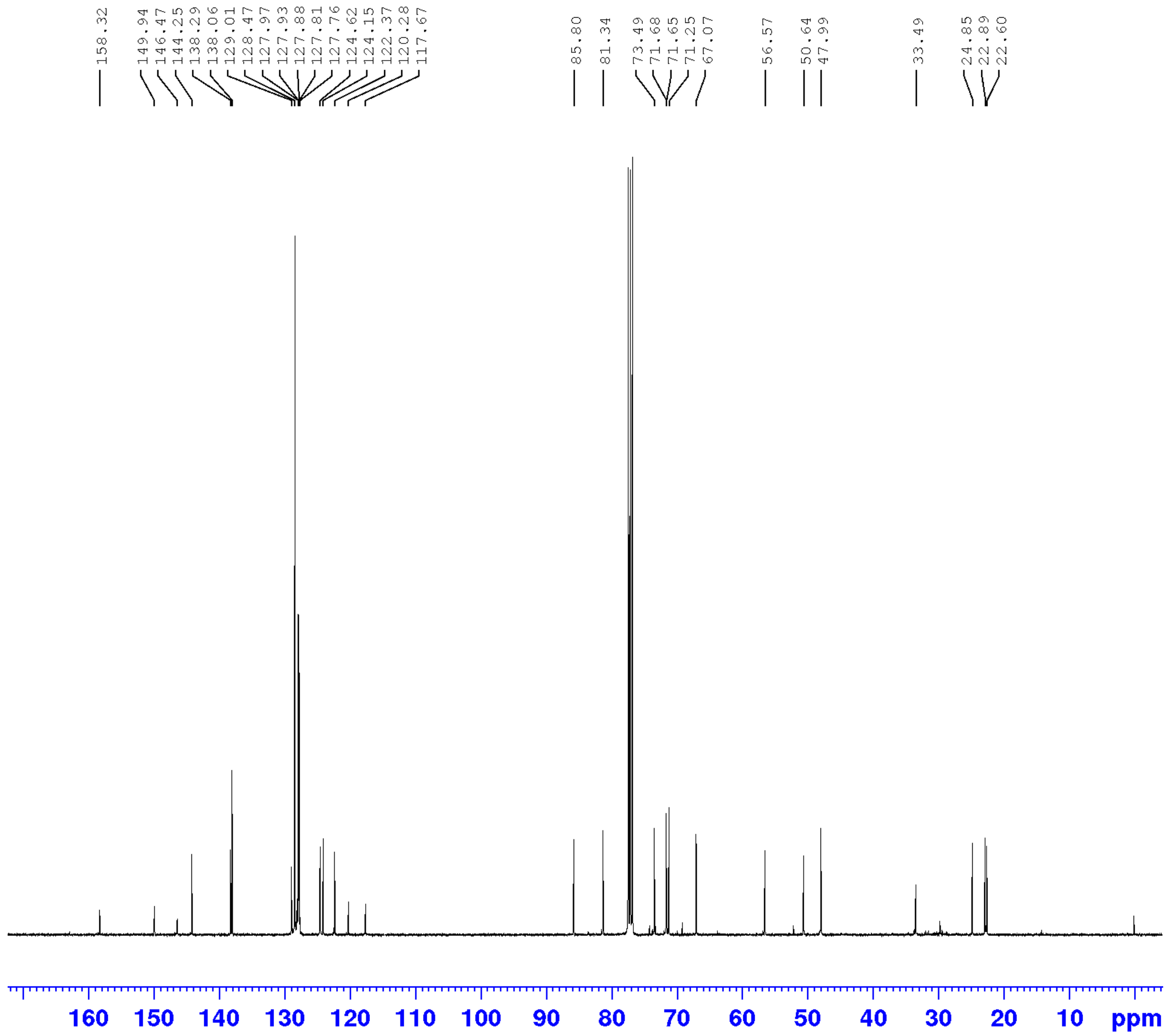

^{1}H-NMR spectrum of compound **20a** (CDCl3, 400.13 MHz)

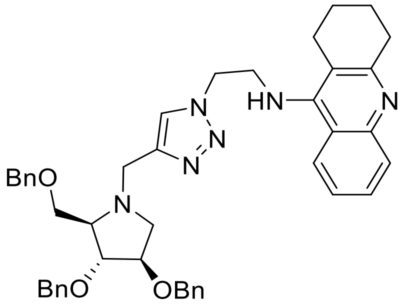

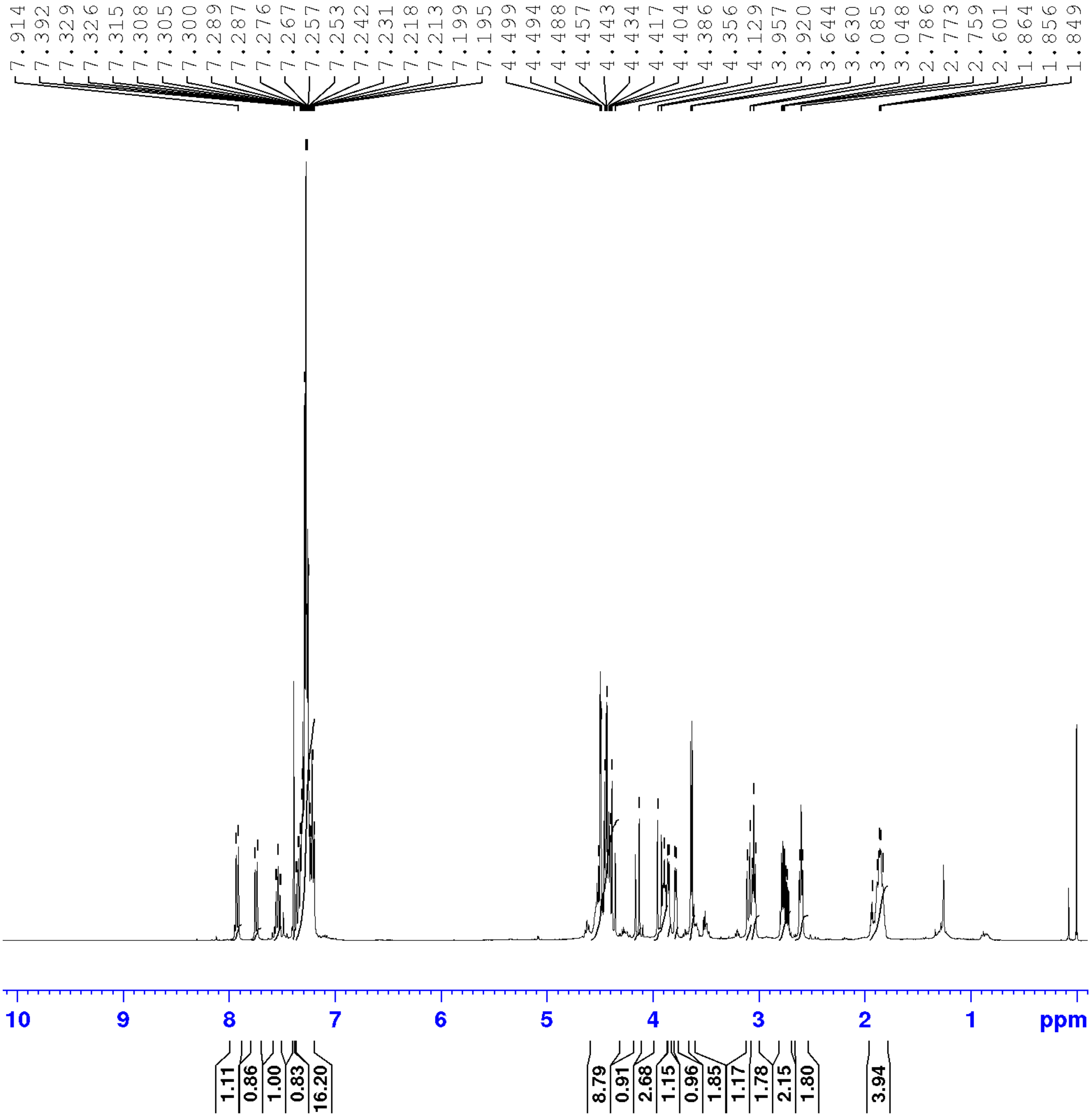

^{13}C-NMR spectrum of compound **20a** ($CDCl_3$, 100.61 MHz)

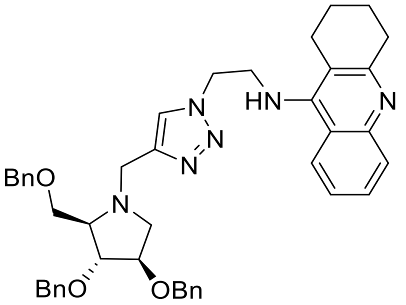

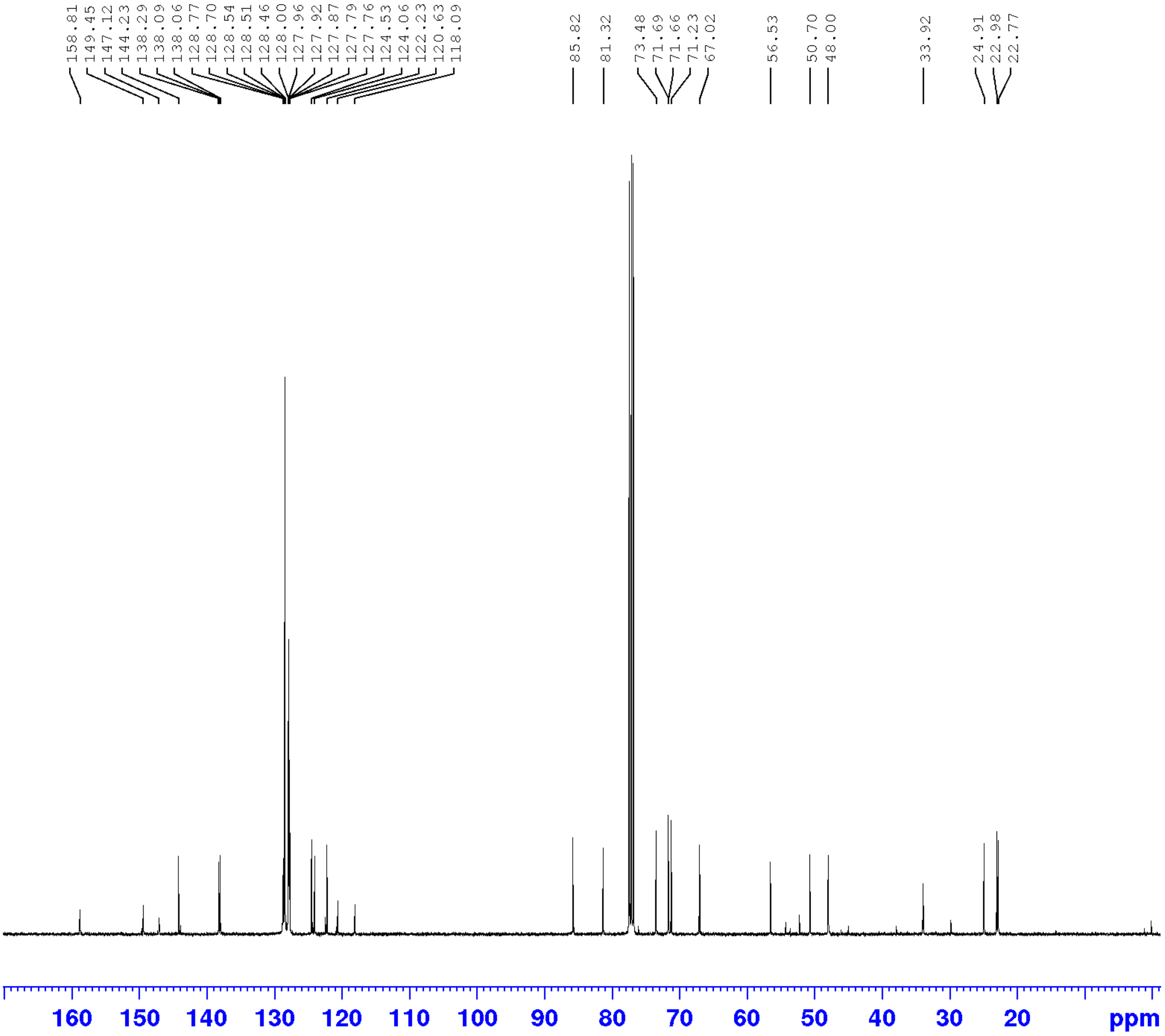

^{1}H-NMR spectrum of compound **21b** ($CDCl_3$, 400.13 MHz)

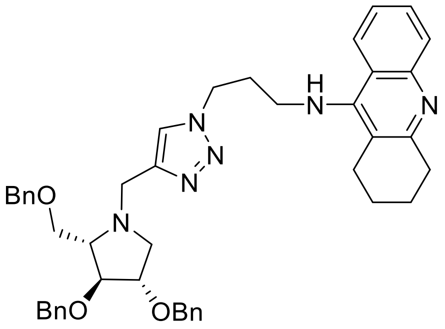

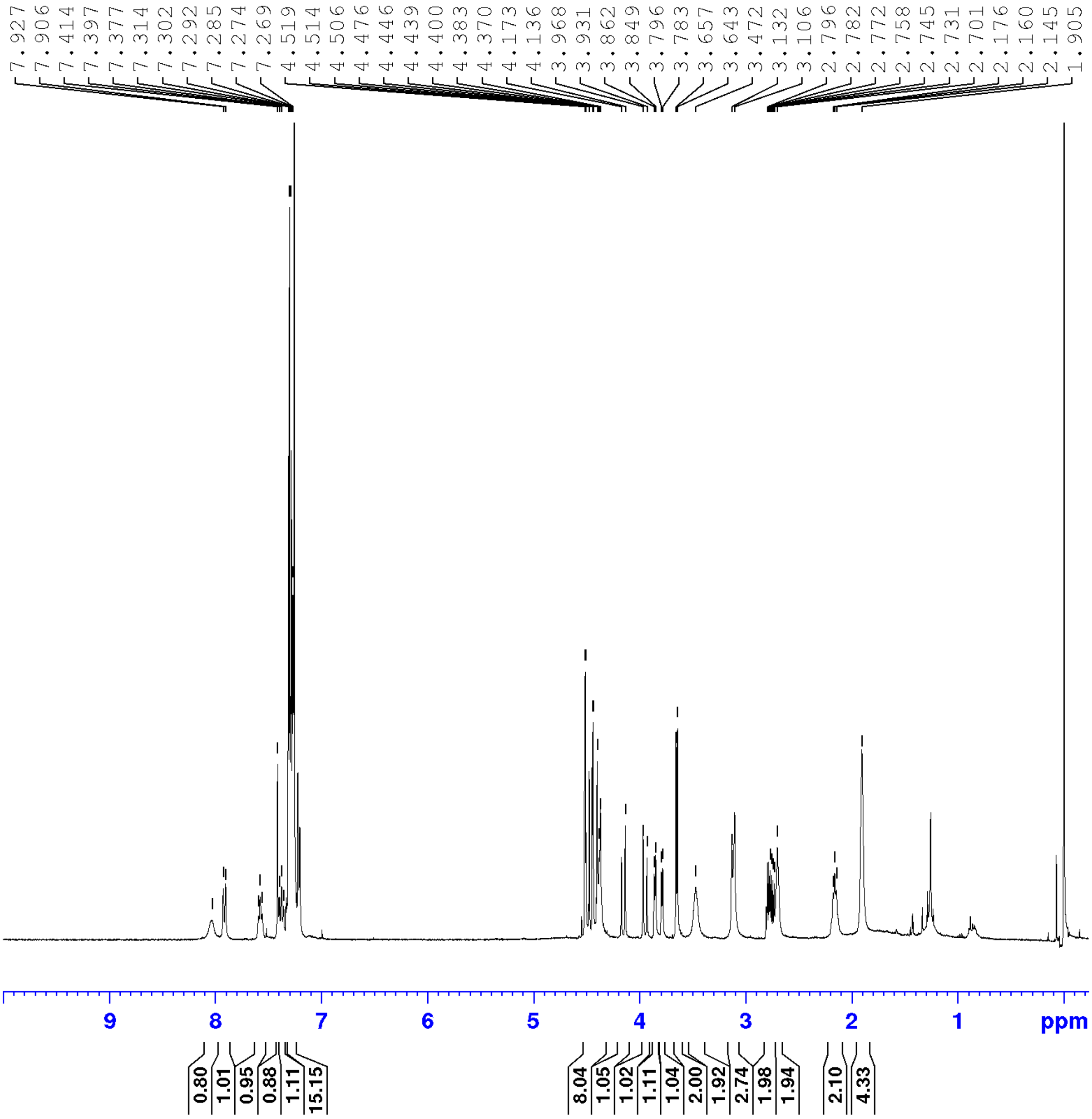

^{13}C-NMR spectrum of compound **21b** ($CDCl_3$, 100.61 MHz)

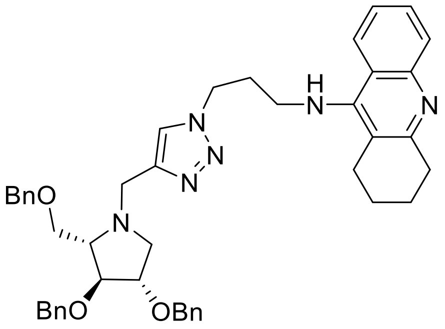

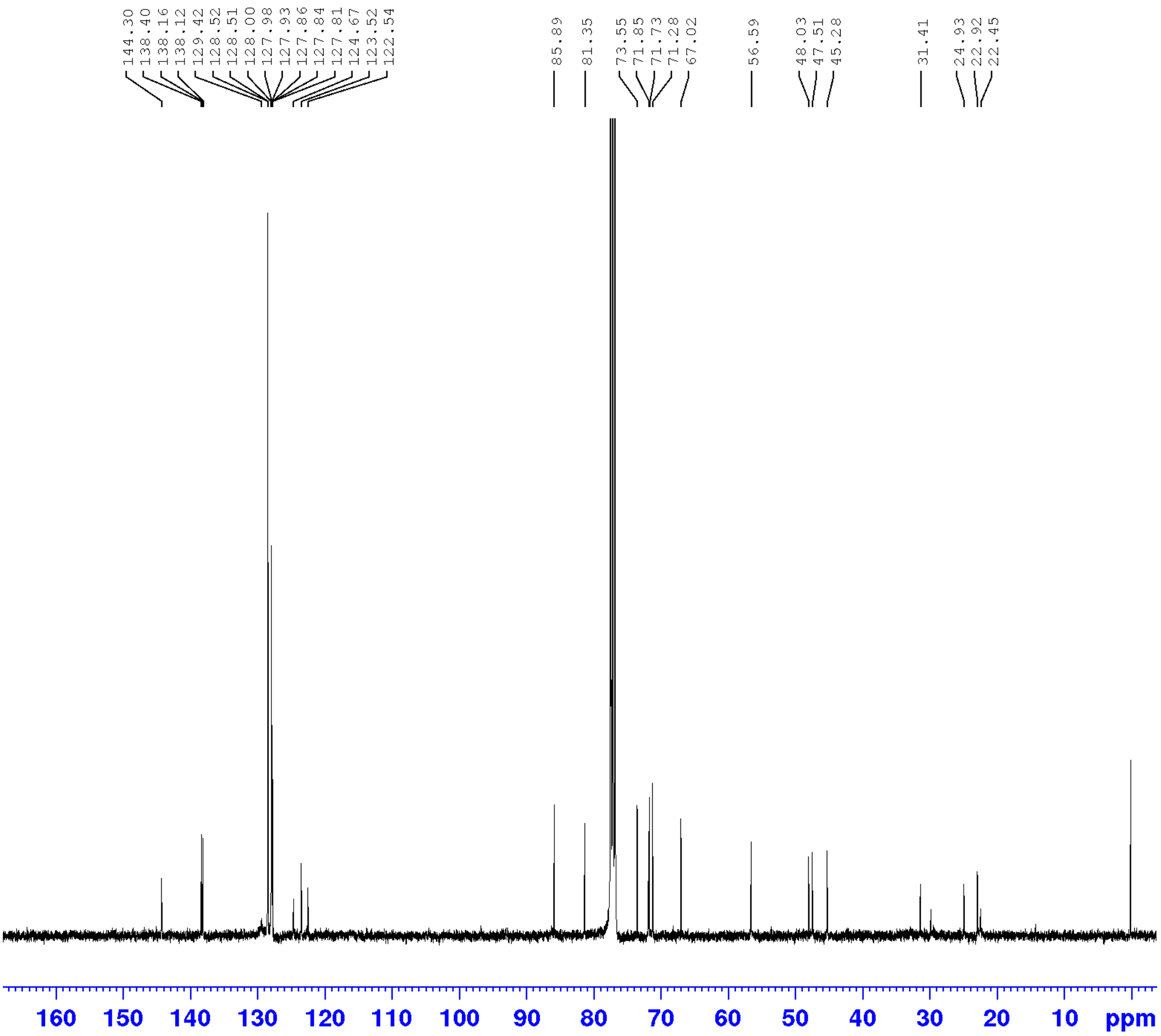

^{1}H-NMR spectrum of compound **21a** ($CDCl_3$, 400.13 MHz)

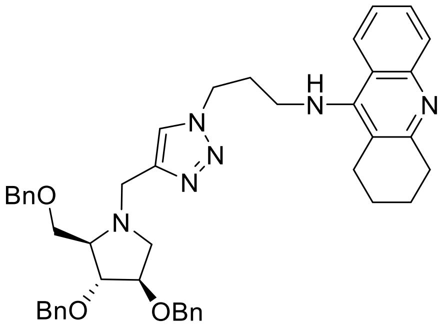

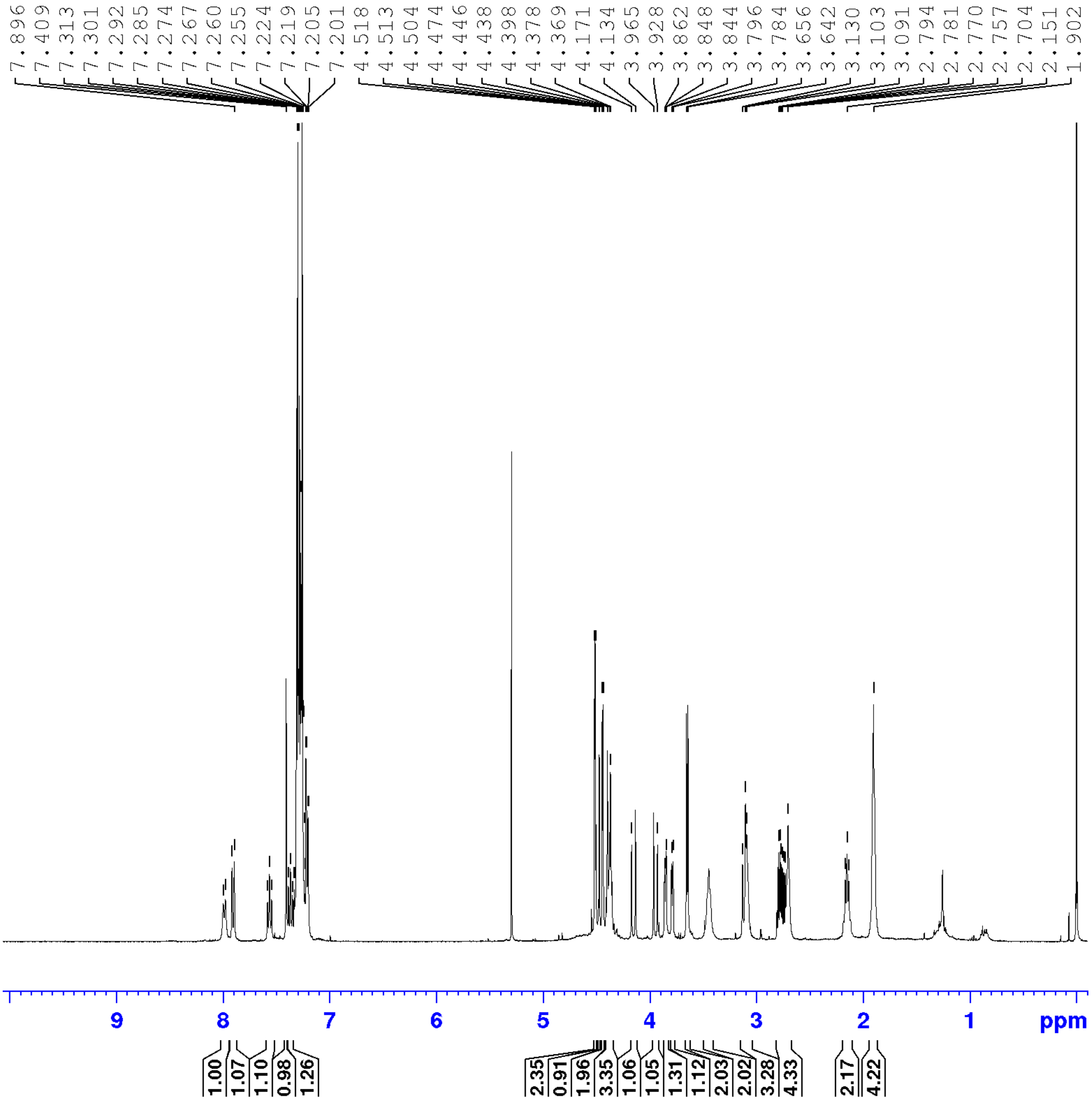

^{13}C-NMR spectrum of compound **21a** ($CDCl_3$, 100.61 MHz)

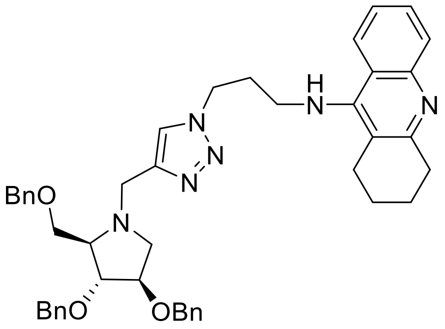

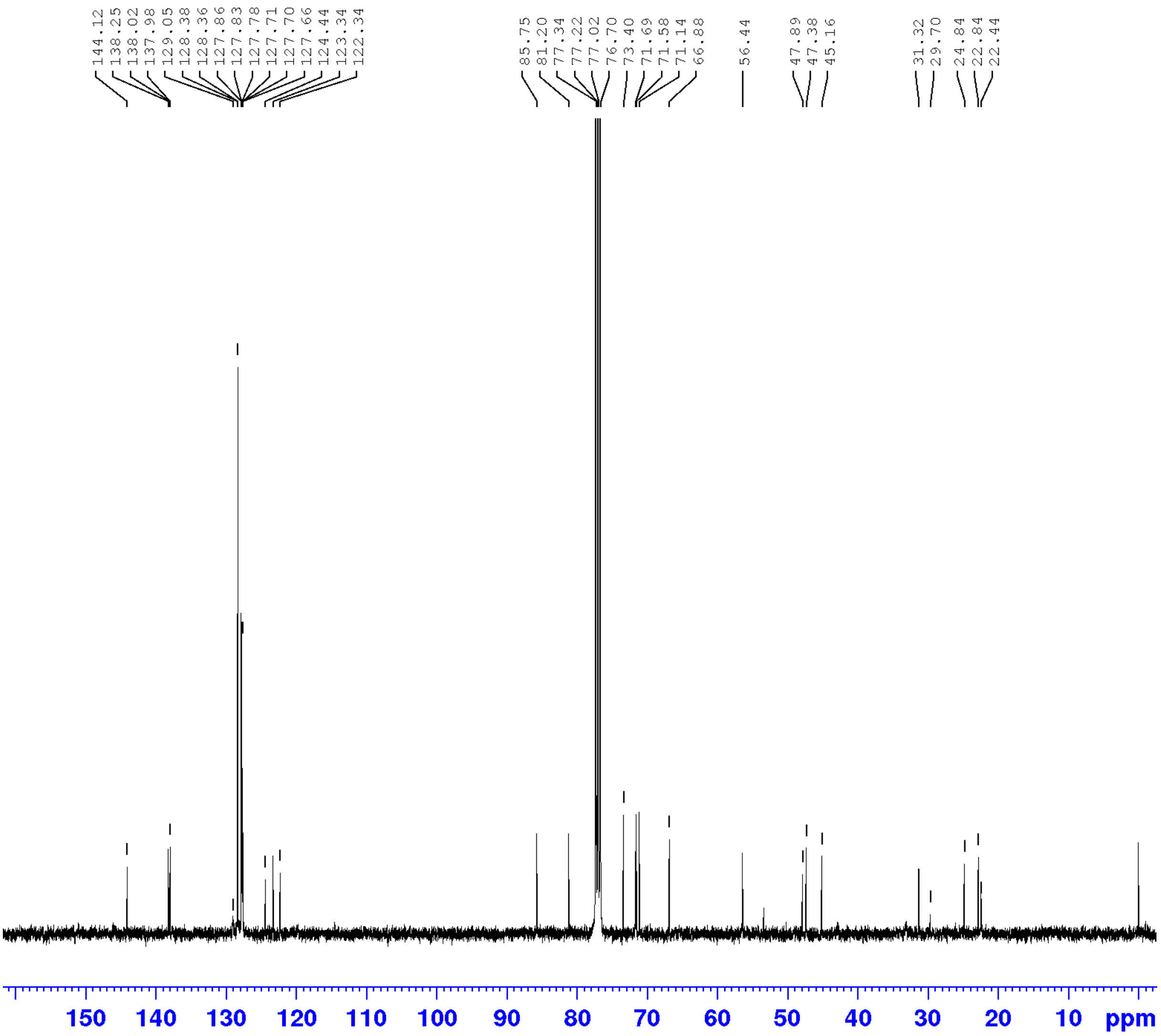

^{1}H-NMR spectrum of compound **22a** ($CDCl_3$, 400.13 MHz)

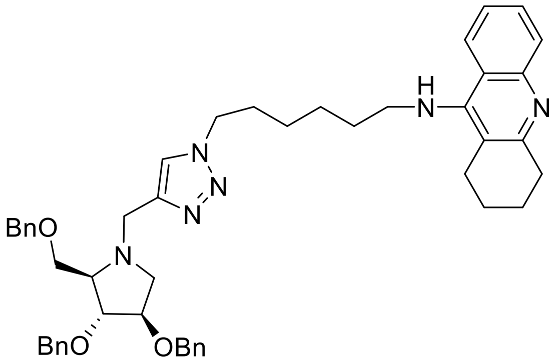

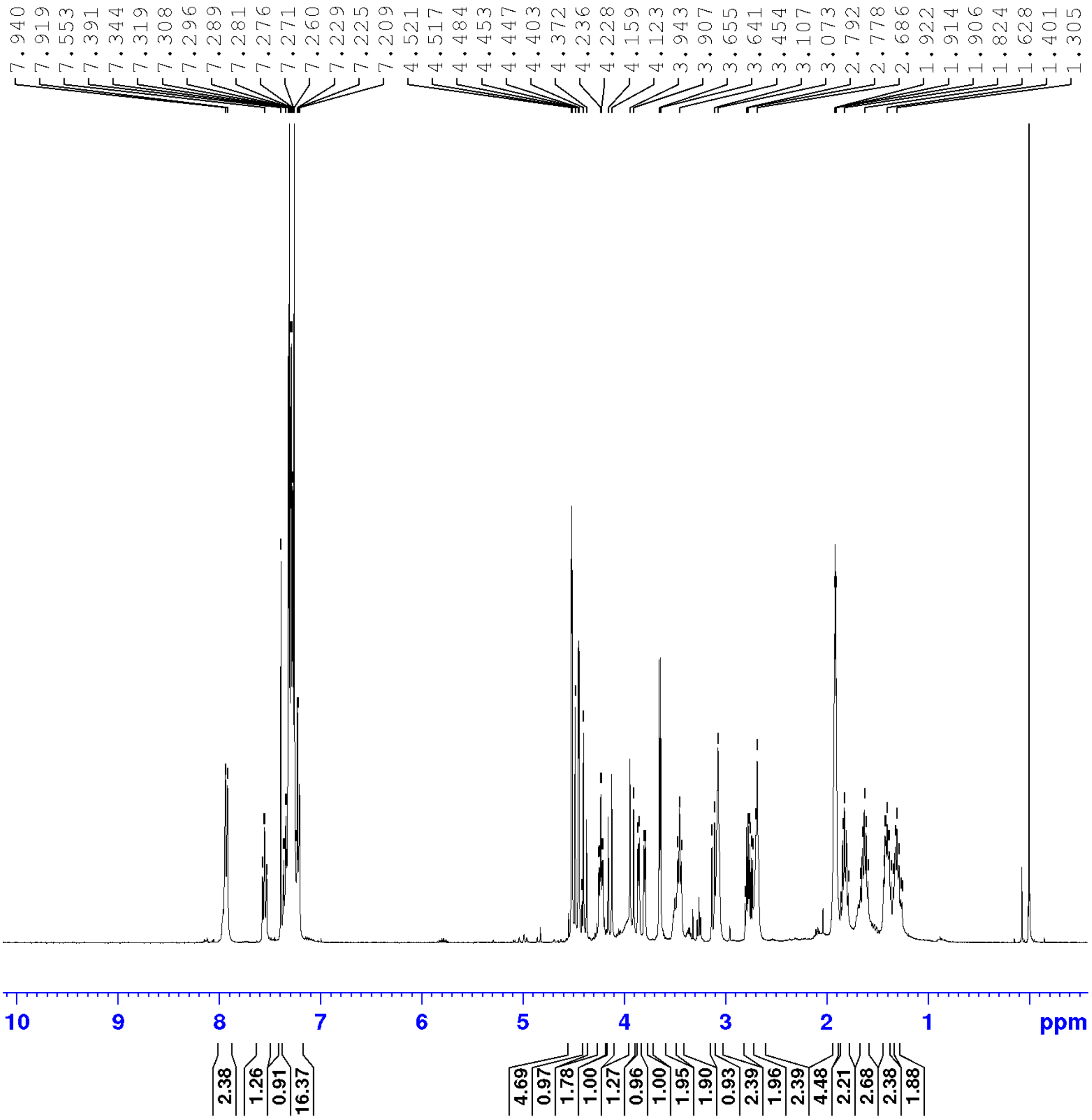

^{13}C-NMR spectrum of compound **22a** ($CDCl_3$, 100.61 MHz)

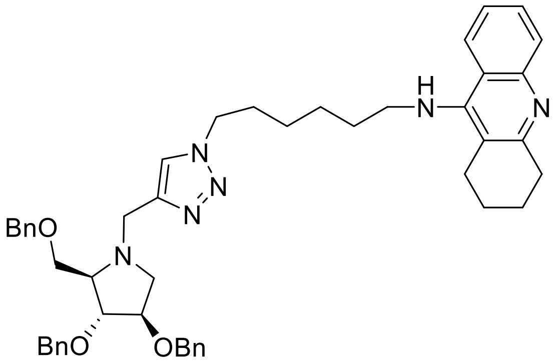

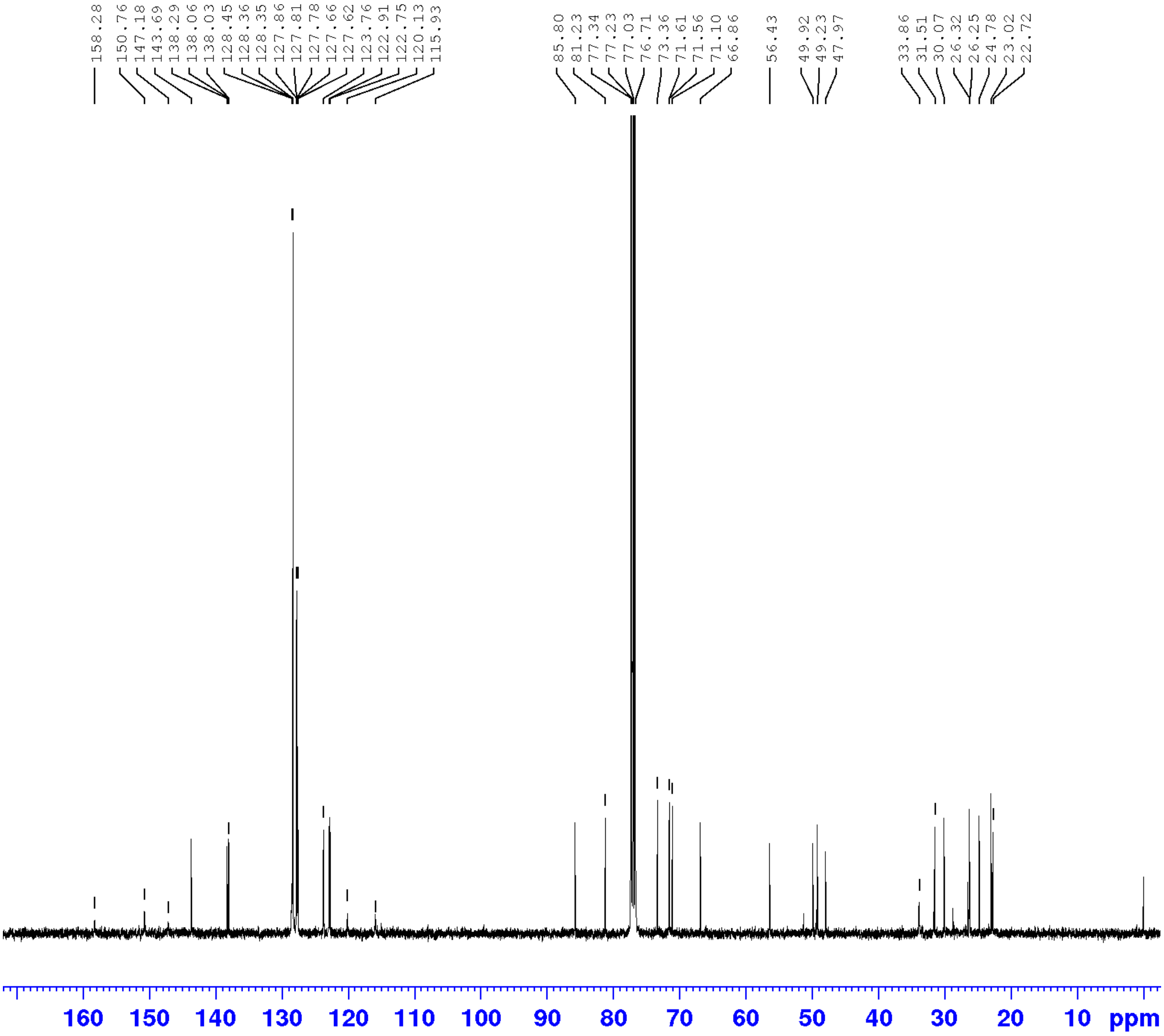

^{1}H-NMR spectrum of compound **9a** (D_2O, 400.13 MHz)

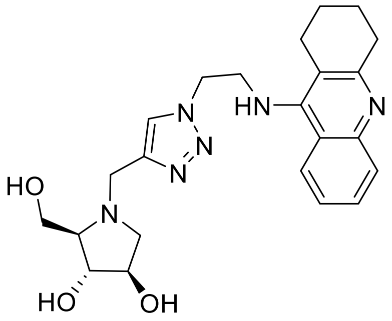

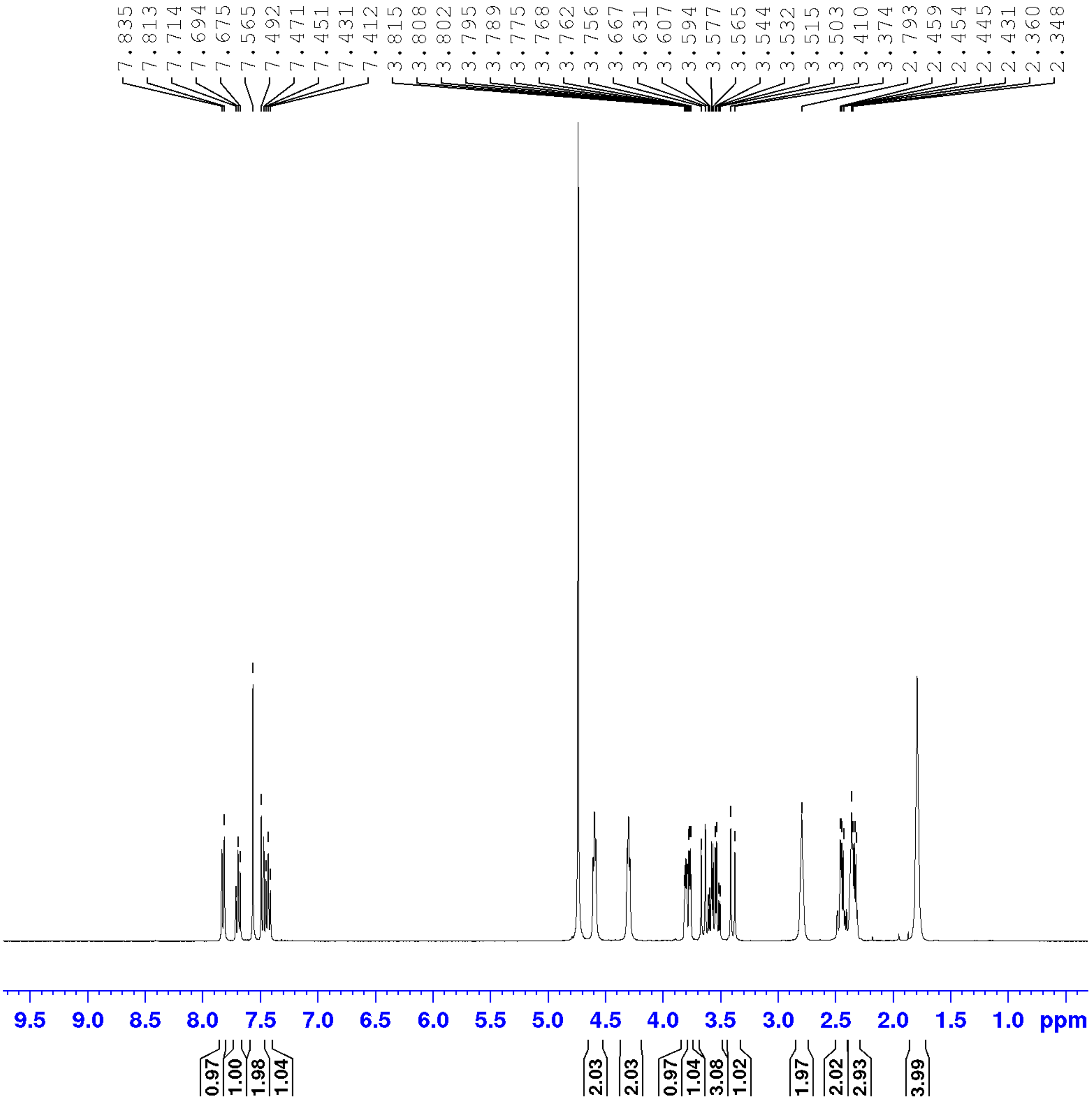

^{13}C-NMR spectrum of compound **9a** (D_2O, 100.61 MHz)

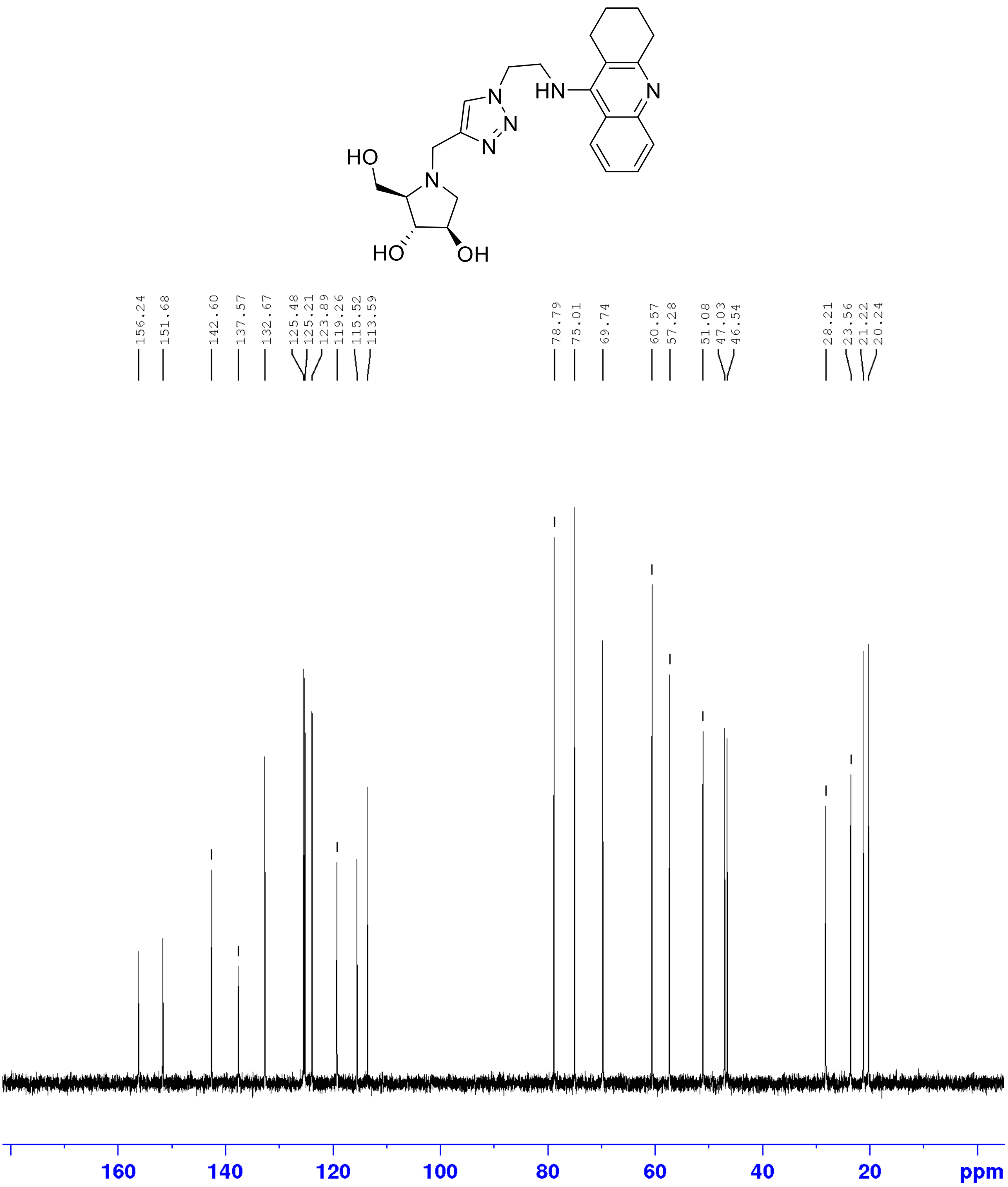

^{1}H-NMR spectrum of compound **9b** (D_2O, 400.13 MHz)

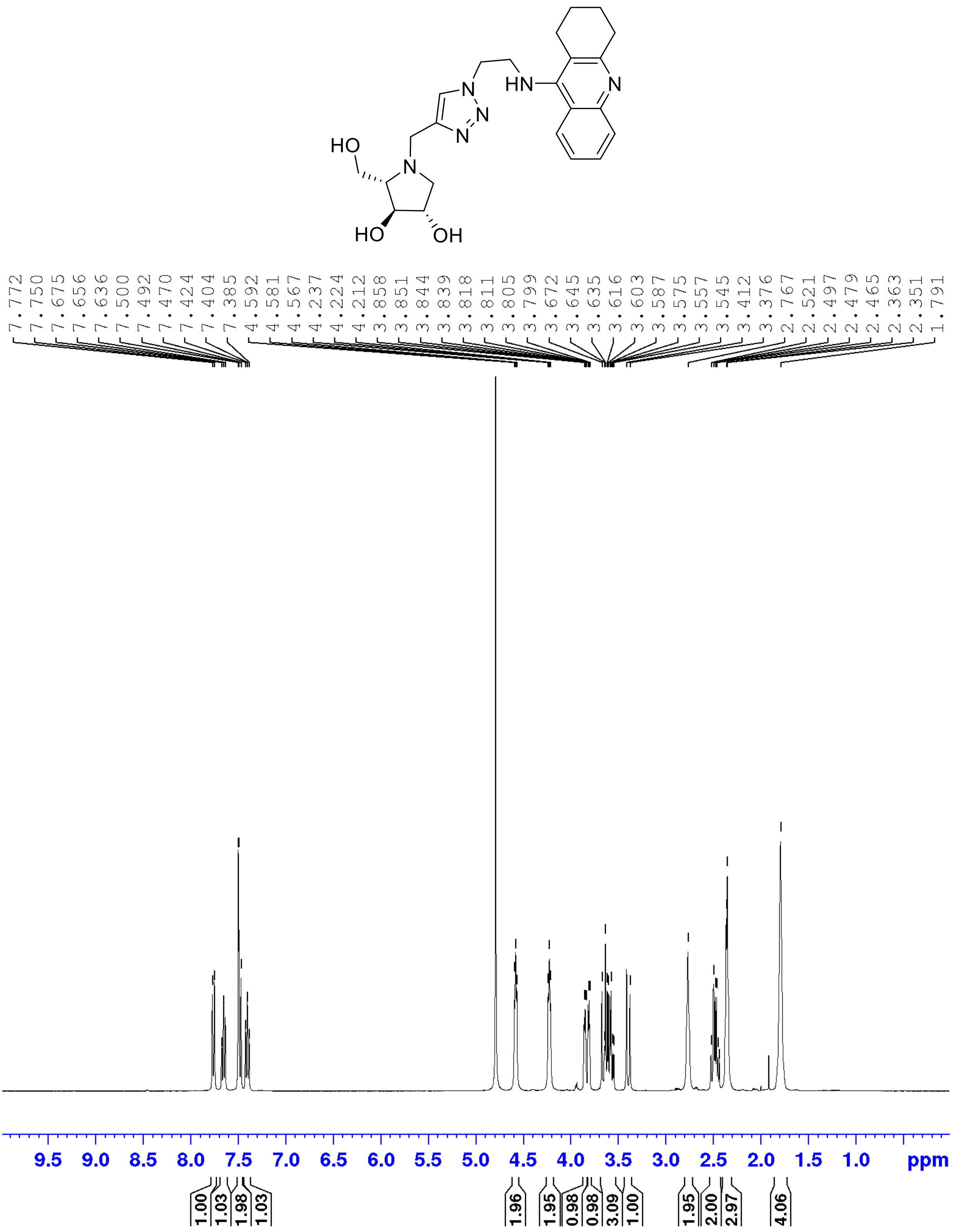

^{13}C-NMR spectrum of compound **9b** (D_2O, 100.61 MHz)

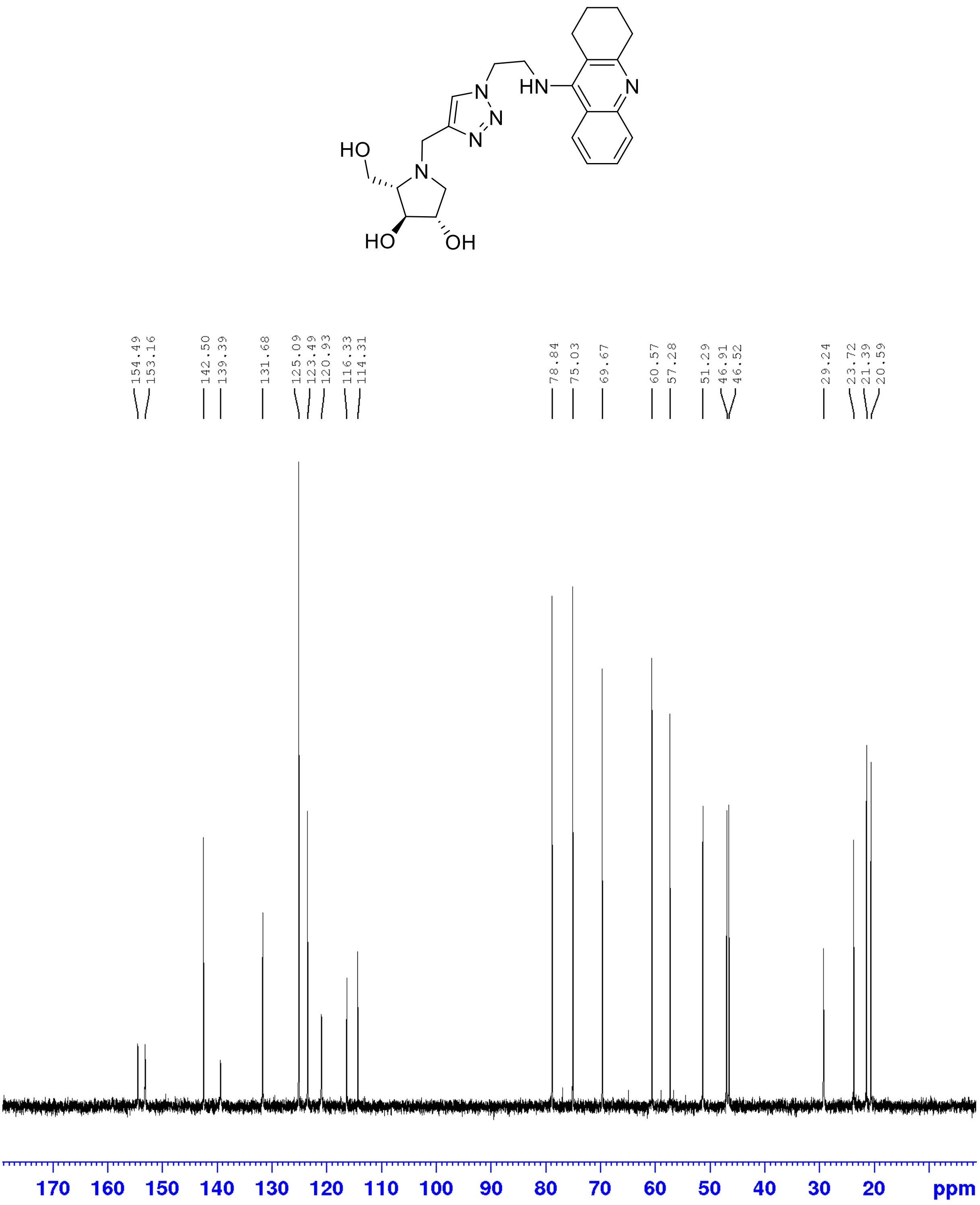

^{1}H-NMR spectrum of compound **10a** (CD_3OD, 400.13 MHz)

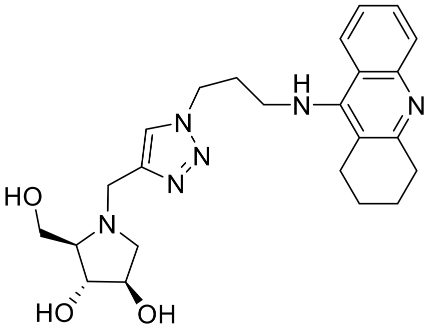

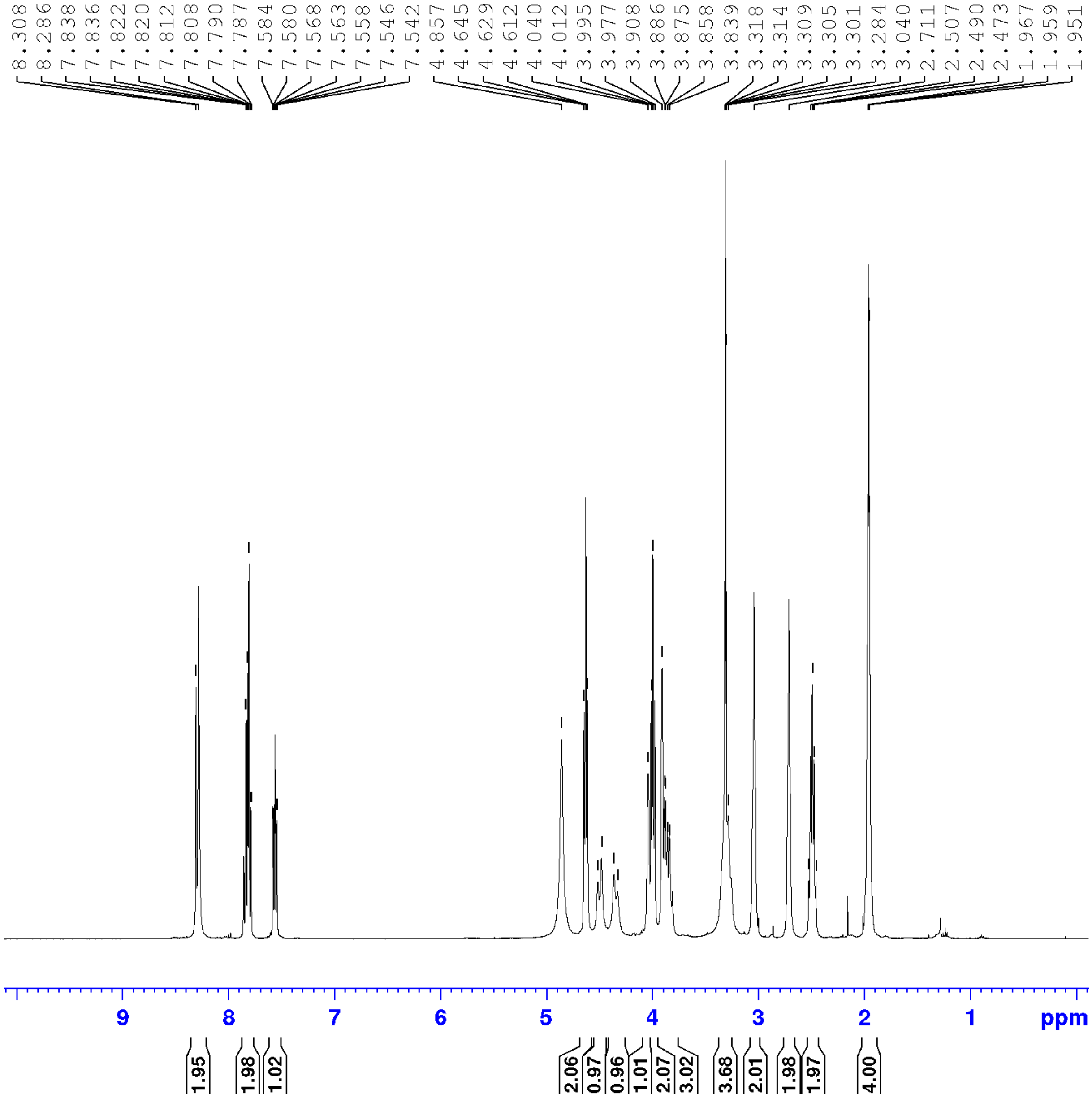

^{13}C-NMR spectrum of compound **10a** (CD_3OD, 100.61 MHz)

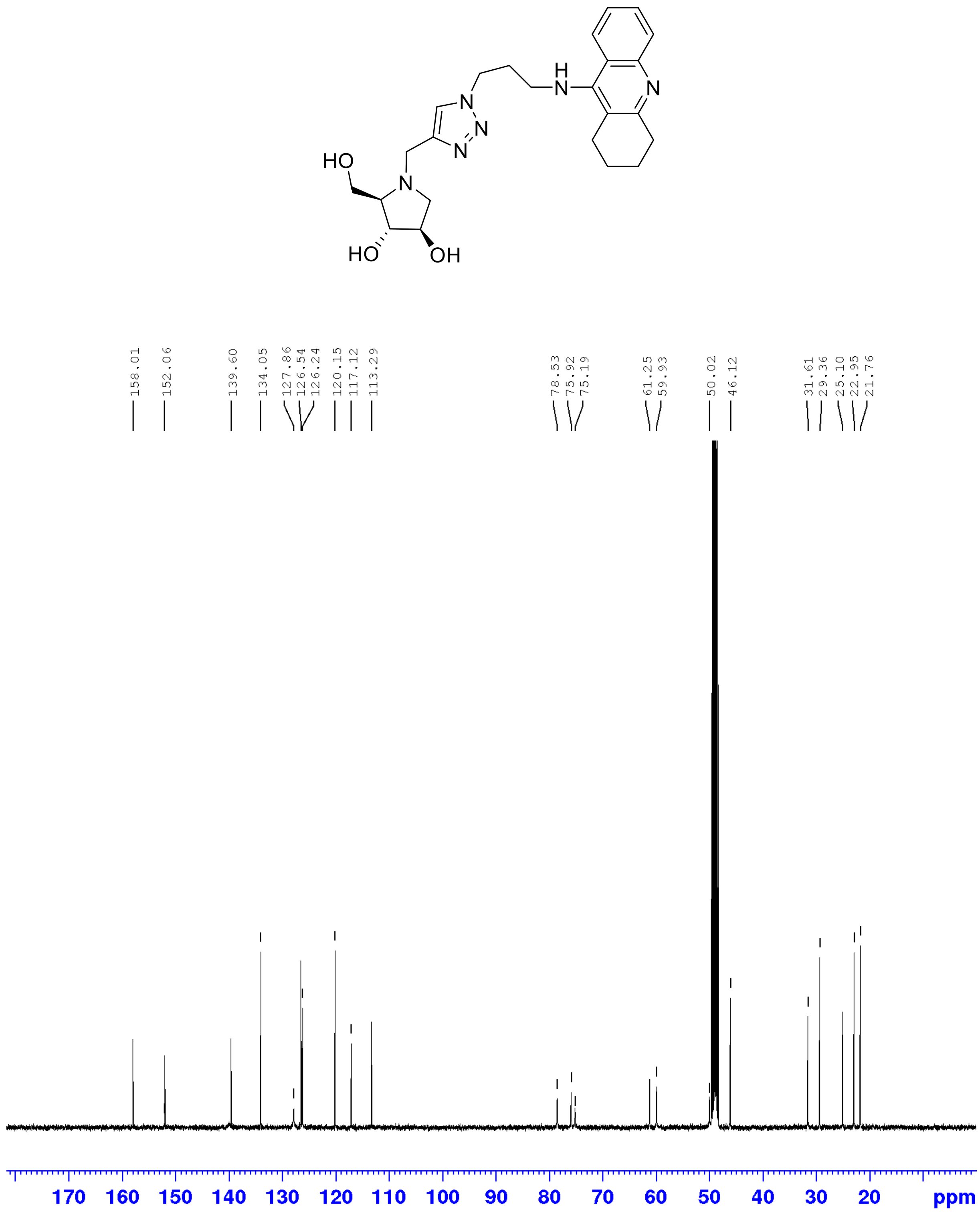

^{1}H-NMR spectrum of compound **10b** (CD$_3$OD, 400.13 MHz)

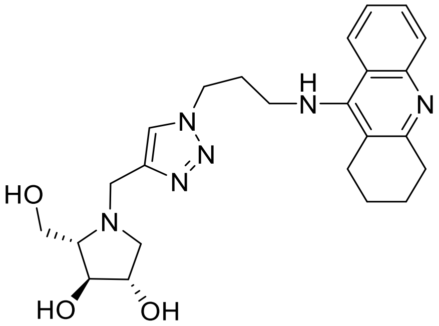

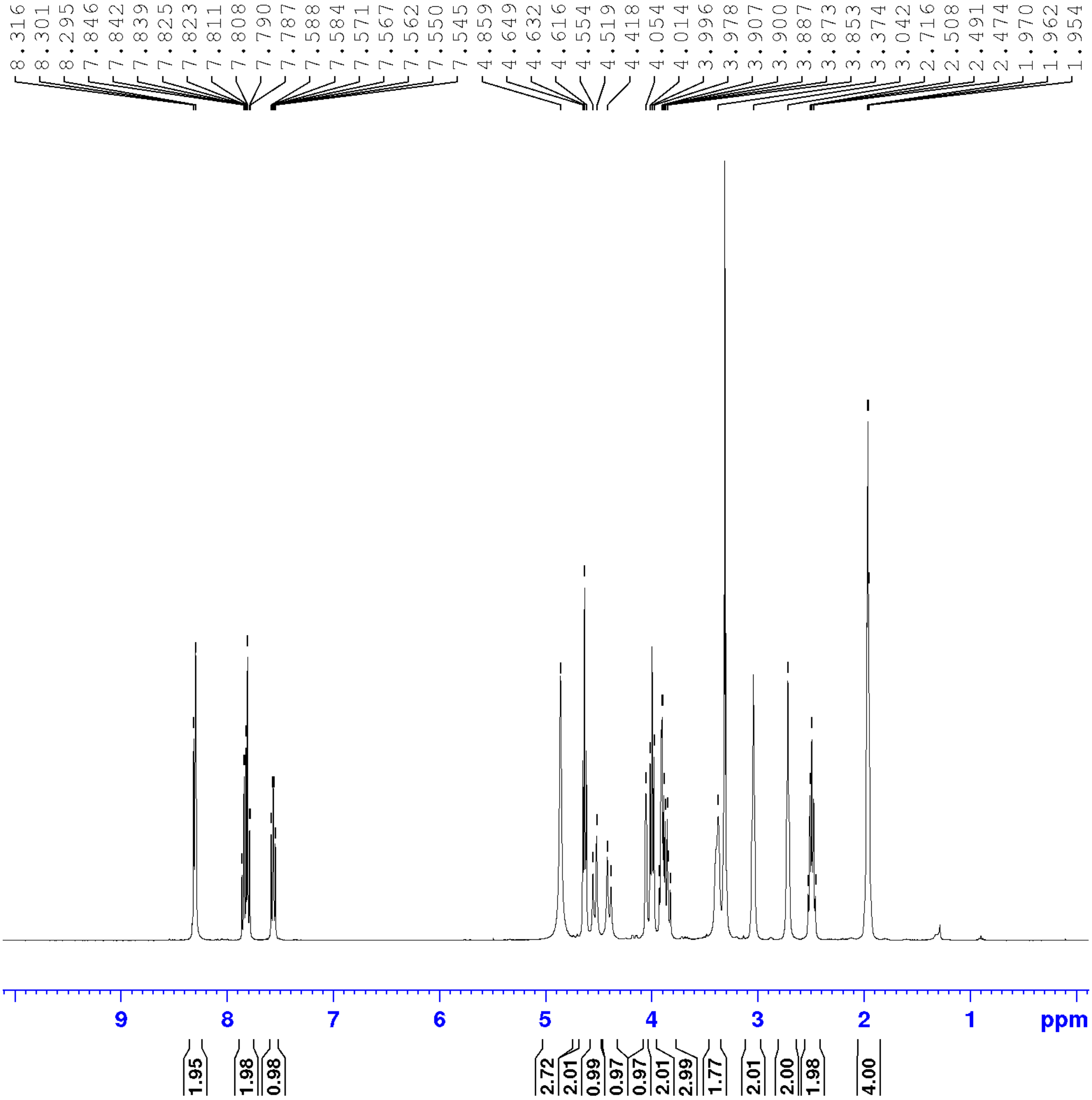

^{13}C-NMR spectrum of compound **10b** (CD_3OD, 100.61 MHz)

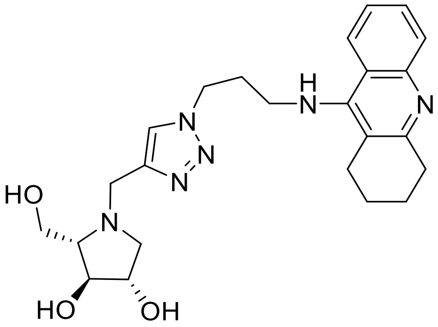

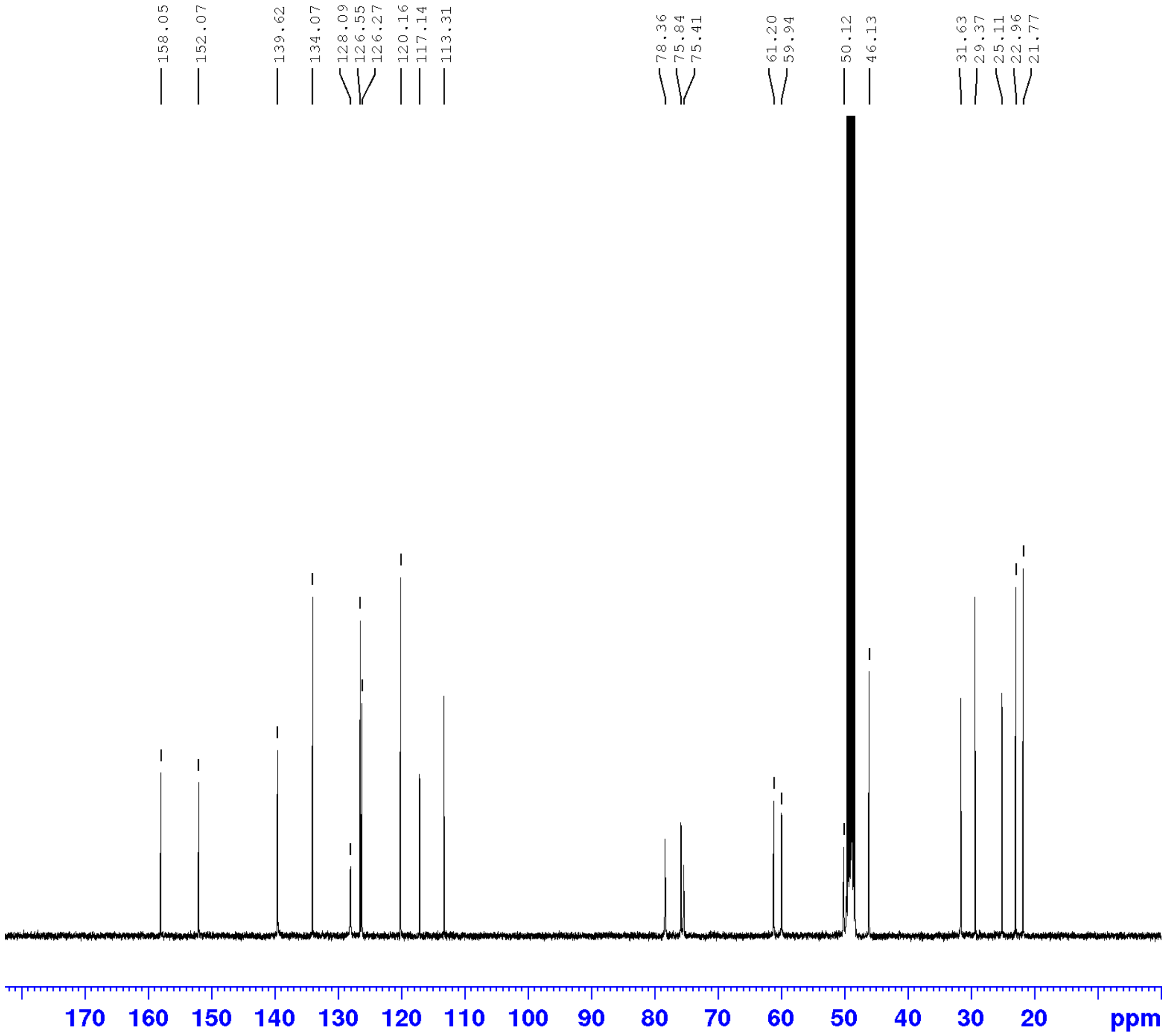

^{1}H-NMR spectrum of compound **11a** (CD_3OD, 400.13 MHz)

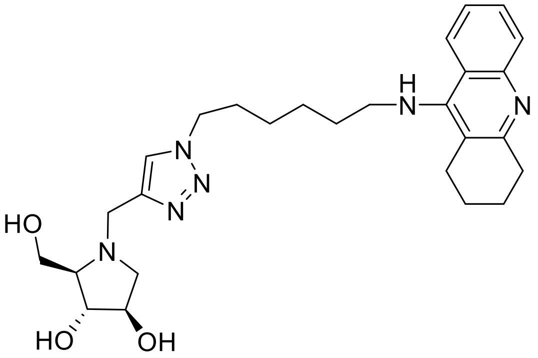

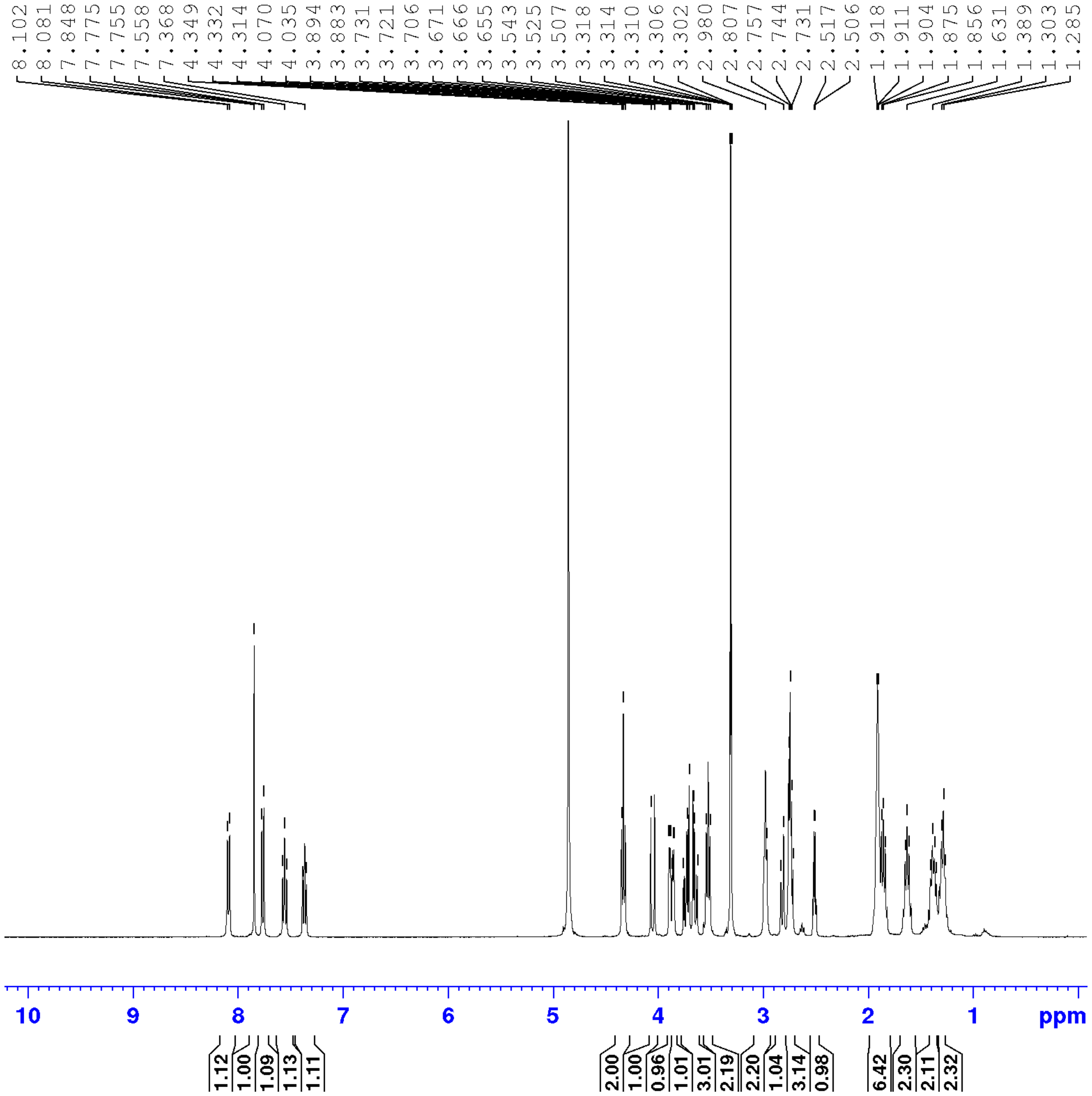

^{13}C-NMR spectrum of compound **11a** (CD_3OD, 100.61 MHz)

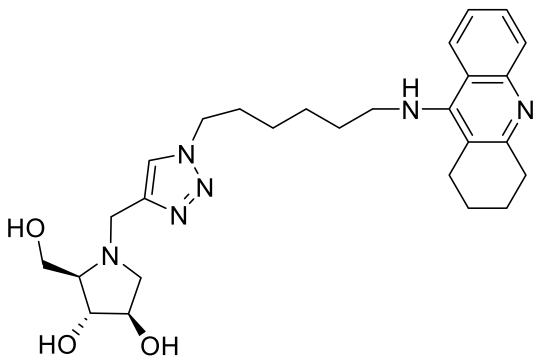
HO
HO
OH
N
N
N
N
H
N
N

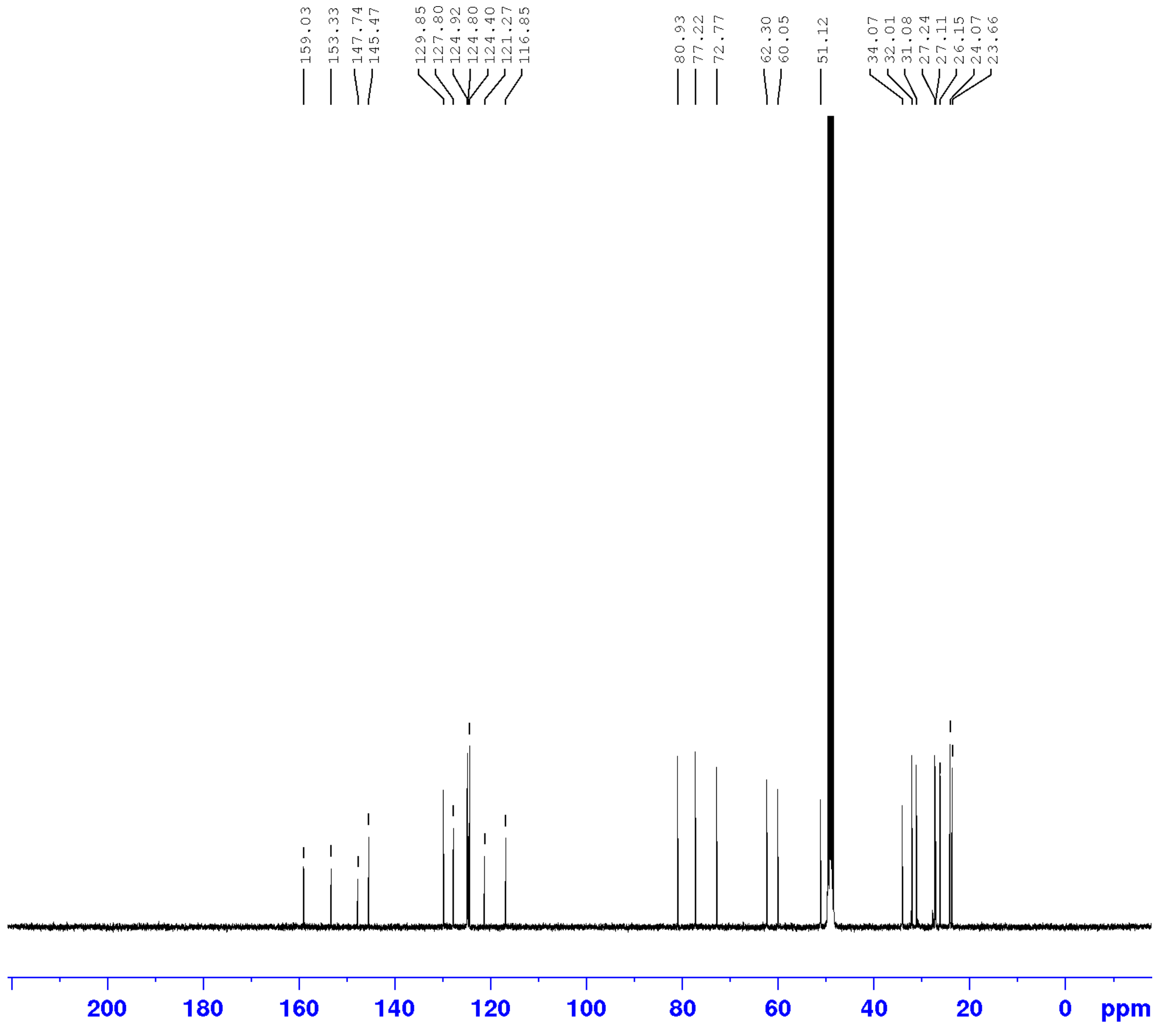
159.03
153.33
147.74
145.47
129.85
127.80
124.92
124.80
124.40
121.27
116.85
80.93
77.22
72.77
62.30
60.05
51.12
34.07
32.01
31.08
27.24
27.11
26.15
24.07
23.66
200
180
160
140
120
100
80
60
40
20
0
ppm

^{1}H-NMR spectrum of compound **11b** (CD_3OD, 400.13 MHz)

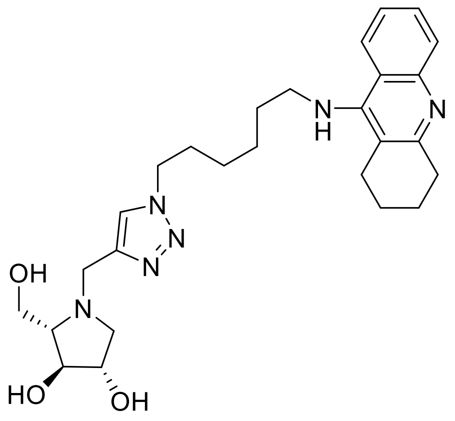

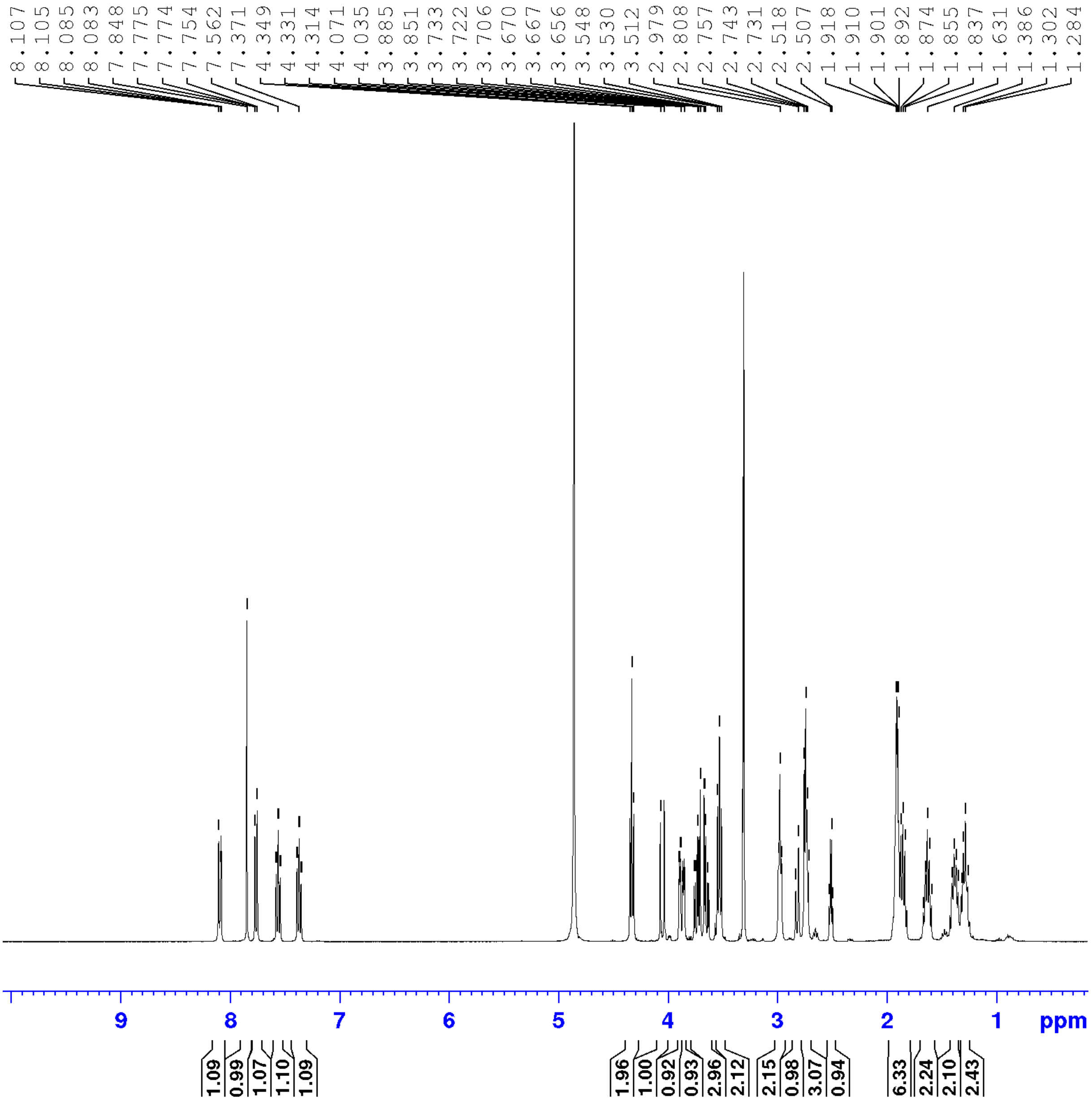

^{13}C-NMR spectrum of compound **11b** (CD_3OD, 100.61 MHz)

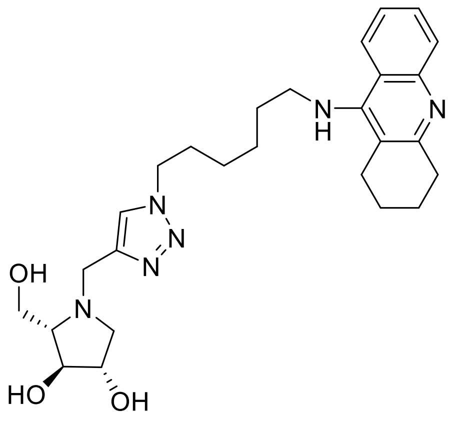

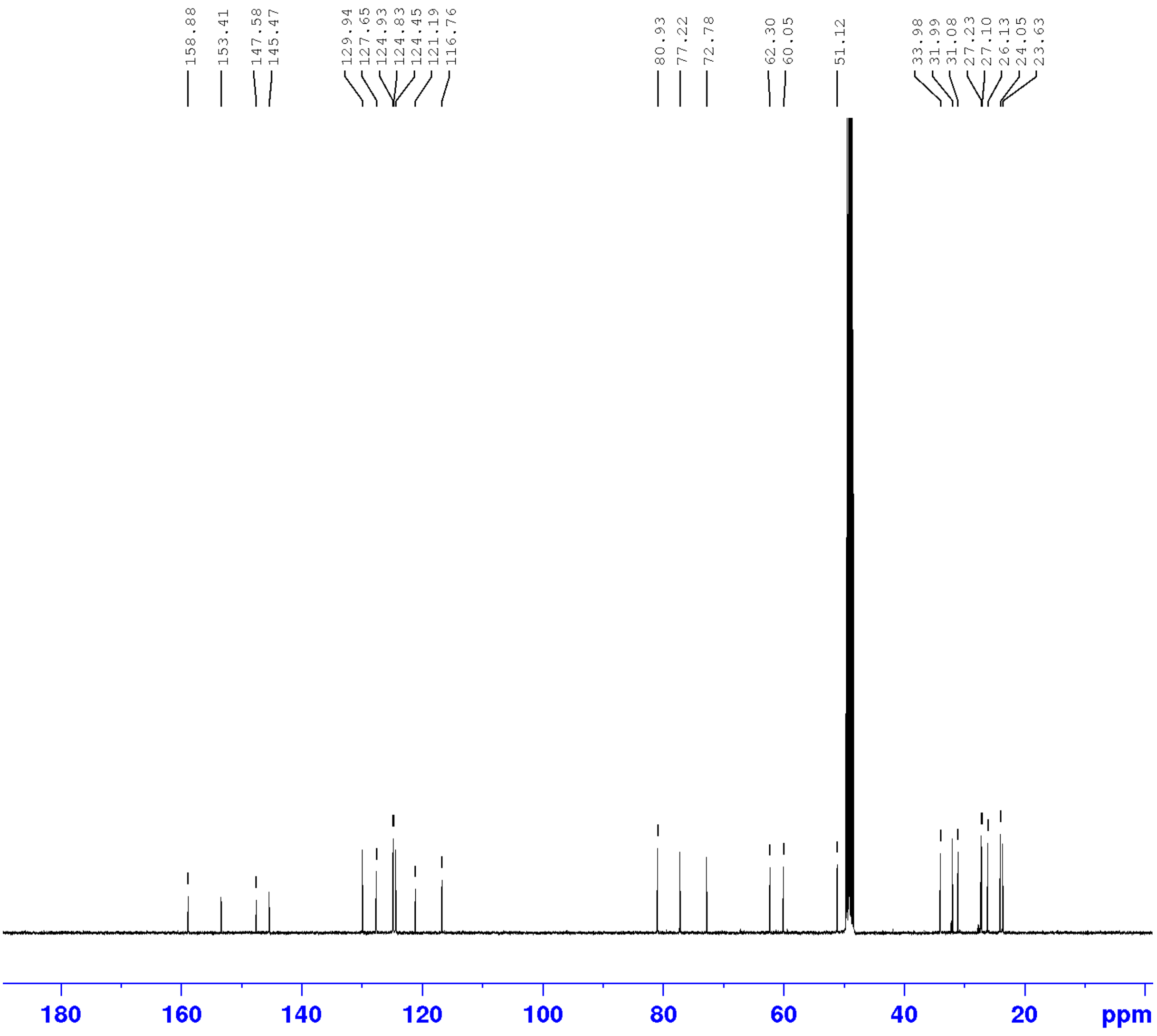

Supplement: Supplemental Material [file IENZ_A_2150762_SM0655.pdf]
